# Modeling Binding Selectivity of Xylene Isomers in Resorcin[4]arene-Based Organo- and Metallo-Cavitands

**DOI:** 10.1021/acs.joc.5c00471

**Published:** 2025-07-01

**Authors:** Gantulga Norjmaa, Yang Yu, Julius Rebek, Fahmi Himo

**Affiliations:** † Department of Chemistry, Arrhenius Laboratory, 7675 Stockholm University, SE-10691 Stockholm, Sweden; ‡ Center for Supramolecular Chemistry and Catalysis and Department of Chemistry, College of Science, 34747 Shanghai University, Shanghai 200444, P. R. China; § The Skaggs Institute for Chemical Biology and Department of Chemistry, 4356 The Scripps Research Institute, 10550 North Torrey Pines Road, La Jolla, California 92037, United States

## Abstract

Binding of xylene isomers to two resorcin[4]­arene-based water-soluble cavitands, one fully organic and one with palladium bridges, is investigated by means of a combination of molecular dynamics simulations and quantum chemical calculations. Experimentally, the metallo-cavitand binds all three isomers but shows a preference for *p*-xylene, while the organo-cavitand prefers *o*-xylene and shows no affinity for *p*-xylene. The cavitands are first characterized and compared in aqueous solution in the absence of guests using classical molecular dynamics simulations. This is followed by a study of the dynamics of the various host–guest complexes. Finally, density functional theory is used to calculate the relative binding free energies. The molecular dynamics simulations show that both host and guest exhibit extensive motions in the complexed state, and the density functional theory calculations yield accurate results on the relative binding free energies.

## Graphical abstract

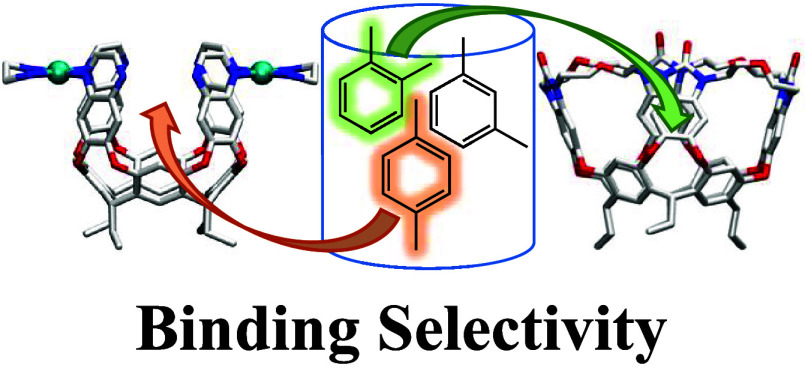

## 1 Introduction

Cavitands are open-ended container hosts developed by Cram and Dalcanale.^1,2^ The compounds are prepared from shallow, bowl-shaped resorcin[4]­arenes by the addition of aromatic panels that bridge adjacent resorcinols (Figure 1). The panels can assume a vase-like conformation with a space inside receptive to guest molecules, or the walls can be flipped outward in an unreceptive kite conformation.^3^ The vase-like shapes can be stabilized by introducing hydrogen bonds^4^ or rigid, covalent bridges^5^ between adjacent walls or opposite walls.^6^

**Figure 1:**
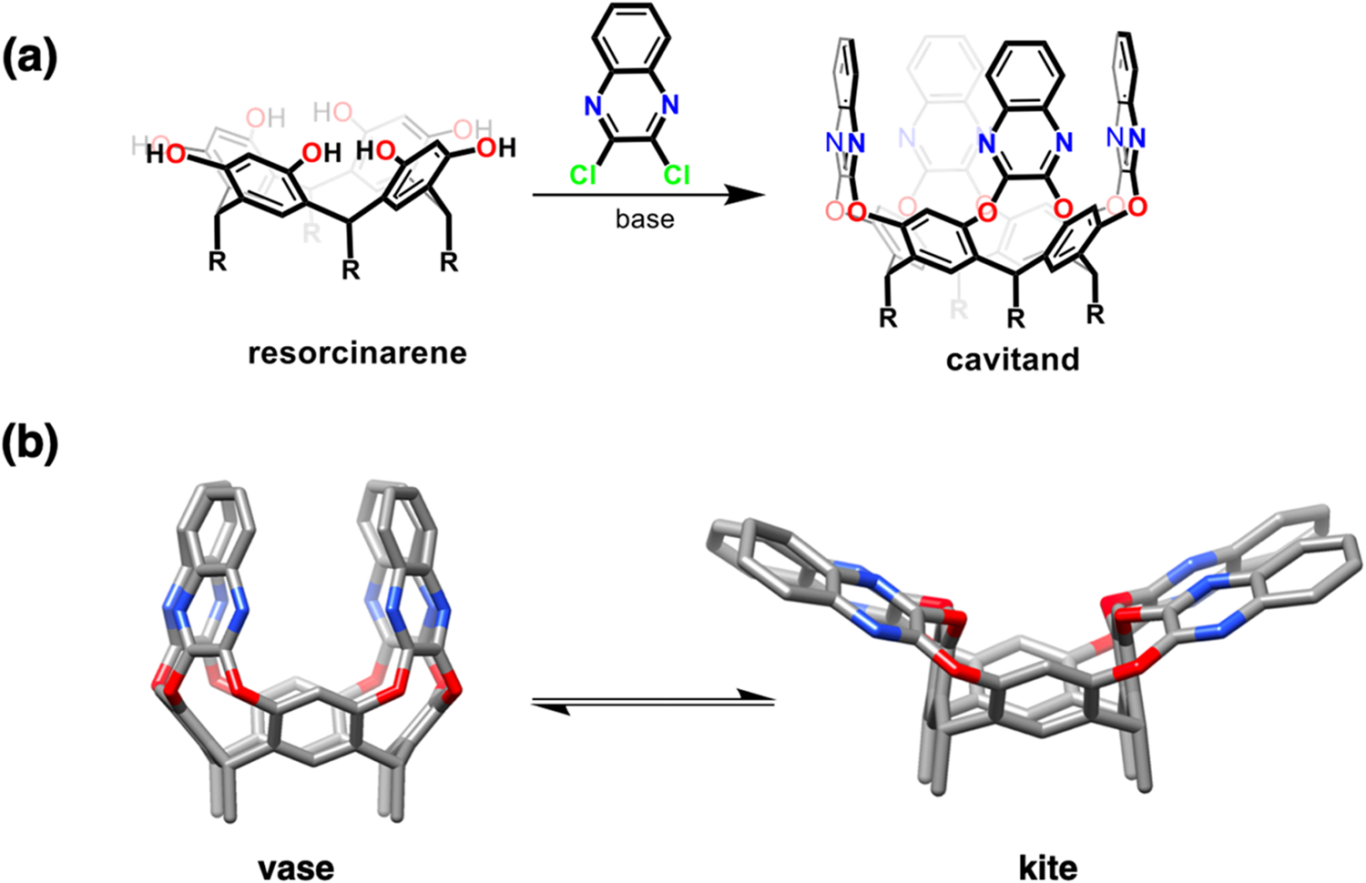
(a) Schematic representation of the synthesis of a cavitand and (b) molecular representation for the equilibrium between vase and kite cavitand conformations.

In recent work, Rebek, Yu, and co-workers recruited metal–ligand interactions to stabilize the vase conformation. They prepared a water-soluble cavitand (Figure 2)^7^ that was an isomer of an earlier compound and positioned the nitrogens of the quinoxaline panels near the upper rim of the cavitand’s walls. The new cavitand bound two atoms of Pd^II^ when exposed to the metal ion in water, and bound small organic compounds in its cavity. In addition, a cavitand whose vase conformation was stabilized by a flexible bridge between the adjacent walls was also prepared (Figure 2).^8^ In the present paper, these cavitands will be referred to as metallo-cavitand (**MCav**) and organo-cavitand (**OCav**), respectively.

**Figure 2:**
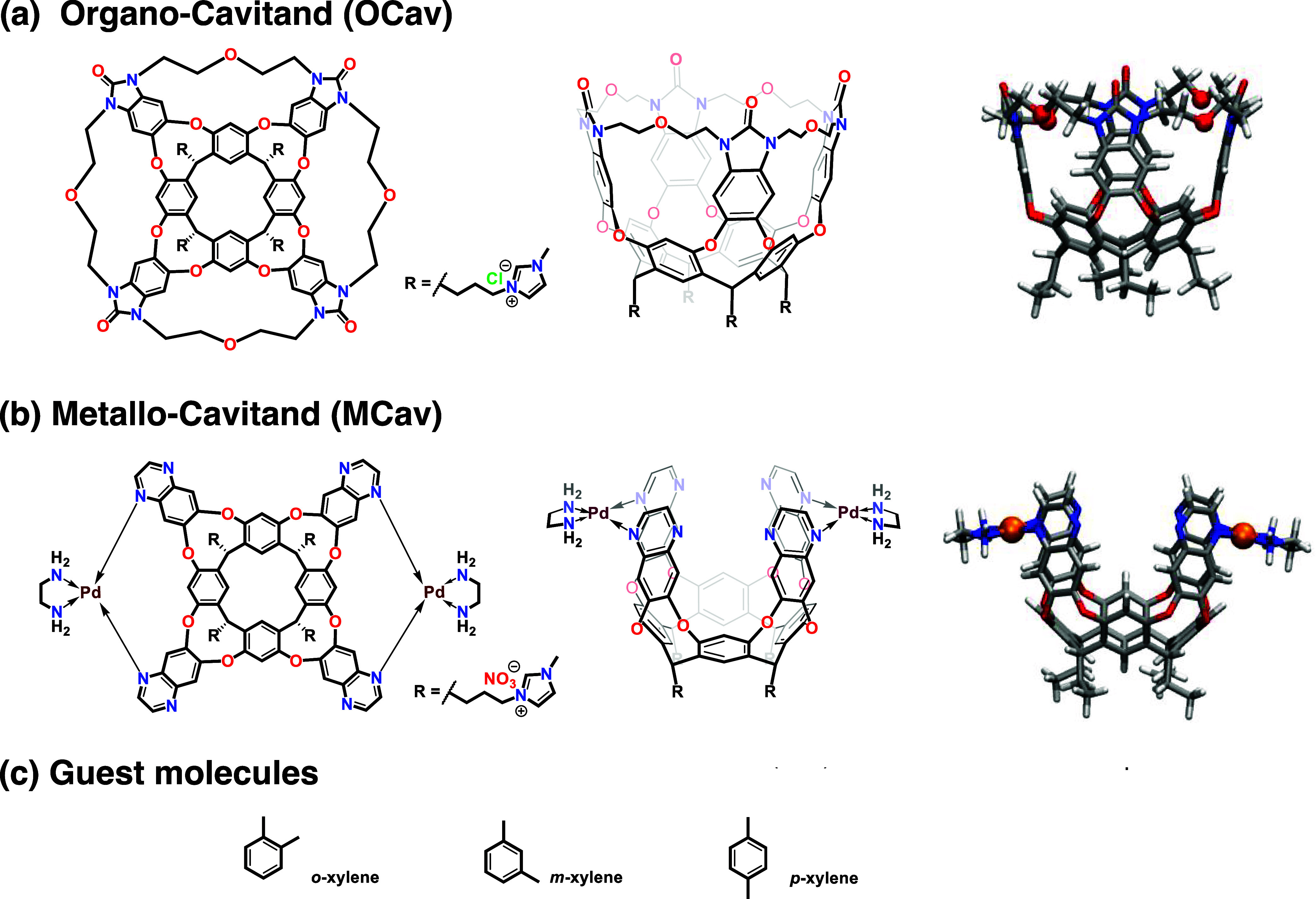
Schematic and molecular representations of (a) organo-cavitand and (b) metallo-cavitand hosts. (c) Xylene isomers are evaluated as guest molecules in the present work.

Unexpected selectivities in binding xylene isomers were encountered in both cavitands using NMR studies,^8,9^ as the xylenes have the same molecular weight, size, hydrophobicity, and surface area, and both the **MCav** and **OCav** can assume shapes that have enough space to accommodate any of the xylenes. The **MCav** bound all three isomers but showed a preference for *p*-xylene and other *p*-substituted toluene derivatives, perhaps reflecting the narrowed opening at its upper rim.^9^ The **OCav** bound both *m*- and *o*-xylenes but preferred the latter, and it showed no affinity for *p*-xylene at all.^8^

Given the industrial importance of *p*-xylene as a precursor to terephthalic acid and its derivatives in the polymer industry, and the close boiling points of the xylenes that make their separation by distillation difficult and energy demanding,^10^ materials that aid xylene purification are of interest. A recent and influential publication by Sholl and Lively^11^ has triggered a cottage industry of low-energy approaches to xylene separation, and basic understanding of the binding and selectivity in various host systems is desirable.

These separation methods include the use of a number of supramolecular systems, such as metal organic frameworks (MOFs), covalent organic frameworks (COFs), molecular pillar­[*n*]­arene crystals, and cucurbit[7]­uril macrocycles.^12–18^

In the present work, we focus on the two resorcin[4]­arene-based cavitands developed by Rebek, Yu and co-workers (Figure 2). These receptors work by simple extraction or in U-tube transport, and are water-soluble. We employ a combination of molecular dynamics (MD) simulations and density functional theory (DFT) calculations to study the structures and dynamics of the cavitands, with and without the xylene guests, and to calculate their relative free energies to investigate the binding and selectivity of xylene. A similar computational approach to investigate the binding and methylation of amines in an introverted methyl ester cavitand was also used very recently.^19^

## 2 Computational Details

The DFT calculations were done using the B3LYP-D3­(BJ) functional^20–25^ as implemented in the Gaussian 16 software.^26^ Geometry optimizations were performed including the SMD continuum solvent model (with water solvent),^27^ using the LANL2DZ basis set^28,29^ for palladium and the 6–31G­(d,p) basis set for the other atoms. To obtain better energies, single-point calculations were performed on the basis of the optimized geometries using the same solvation model and the 6–311+G­(2d,2p) basis set for all atoms except palladium, for which the LANL2DZ basis was kept. At the same level of theory as the geometry optimizations, free energy corrections were evaluated at 298.15 K using the quasi-rigid-rotor-harmonic-oscillator approach with a cutoff of 100 cm^–1^.^30^

The MD simulations were performed using the pmemd program from the Amber 16 package with the general Amber force field.^31,32^ For the xylenes and the organo-cavitand, the AM1-BCC charges were used,^33^ while for the metallo-cavitand, the RESP charges were used.^34^ The MCPB.py program was used to derive nonstandard parameters for the metal centers (Pd^2+^) of the metallo-cavitand, and the antechamber program was employed to assign atom types and derive atomic charges.^35,36^ The simulation box with an edge of 60 Å was treated under periodic boundary conditions and contains the cavitand host, the xylene guest, and ca. 5000 water molecules (TIP3P) explicitly.^37^ The simulation box with the metallo-cavitand included an additional four chloride counterions to keep the solution neutral. The simulations were performed at constant temperature (298.15 K, using a Langevin thermostat) and pressure (1 bar, using a Monte Carlo barostat).^38^ A cutoff of 9 Å was used for nonbonded interactions. The PME method was used for long-range electrostatics interactions.^39^ After an equilibration period including 5000 minimization steps, 60 ps heating to 298.15 K, and 60 ps NPT equilibration, an NPT production run of 200 ns was performed. For the characterization of cavitands without xylene guests, a production run of 600 ns was performed after the same equilibration procedure. A time step of 2 fs was used in all simulations. The analysis tool process_mdout.perl from the Amber 16 package was used to analyze the MD simulations (see Supporting Information, Figures S1–S8). The UCSF Chimera program was used for clustering 1000 MD structures and obtaining the most populated geometries for each host–guest complex.^40^ The UCSF Chimera program was also used to produce the molecular graphic images in Figures 1, 6, and 7.^40^

## 3 Results and Discussion

In the following sections, we first characterize the two cavitands (**OCav** and **MCav**) in the absence of guests and compare their geometries and dynamics. Next, we discuss the dynamics of the host–guest complexes, before presenting the results obtained by the DFT calculations on the binding energies of the different xylene isomers to each cavitand.

### 3.1 Characterization of Cavitands

We started by analyzing the structures of the two cavitands in aqueous solution using classical MD simulations with explicit solvent molecules. For each cavitand, we performed a simulation of 600 ns with a simulation box that contains ca. 5000 water molecules. We define the distances between the opposite walls of the cavitands as **d1** and **d2**, which are the distances between the centers of mass of the benzene ring of each wall as shown in Figure 3.

**Figure 3:**
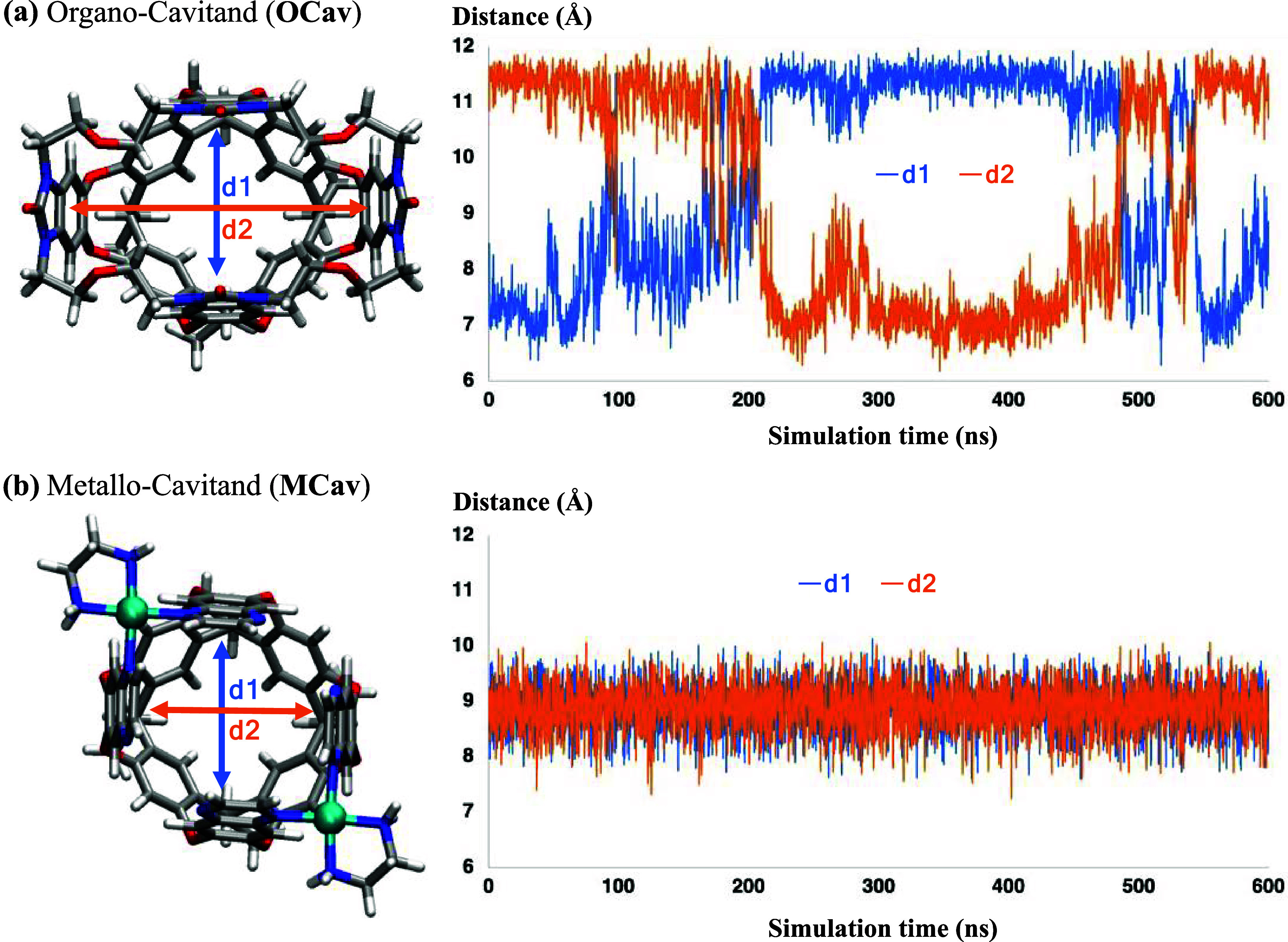
Distances between the walls of (a) organo-cavitand and (b) metallo-cavitand during molecular dynamics simulations.

The simulations show notable differences in shape between the two cavitands. For **OCav**, the **d1** and **d2** distances vary between 6–9 Å for the short one, and 10–12 Å for the long one. This shows that two of the walls are significantly closer than the other two walls, resulting in a rectangular shape at the rim (Figure 3). It is interesting to note that a switch of the short and long distances was observed several times during the simulation.

When the carbonyl oxygen atoms at the top of the walls are selected (instead of the benzene rings) to define the cavitand dimensions, the short distance is even shorter and the long distance is even longer, showing that the cavity of this cavitand is narrower at the top than in the middle (see Supporting Information, Figures S9 and S10). We note furthermore that one of the methylene groups of each diethyl ether bridging group between the walls is located in the interior of the cavitand, resulting in less space at the sides of the rim compared to the middle of the rim.

For **MCav**, the **d1** and **d2** distances were similar during the simulation, ranging between 7–10 Å and showing a rhombic shape at the rim (Figure 3). When the nitrogen atoms that coordinate to the palladium ions are selected to define the distances between the walls, both distances become shorter, indicating that also **MCav** has less space at the top compared to its middle (see Supporting Information, Figures S9 and S10).

On the basis of simulations, we can estimate the dimensions of the interior space of the cavitands. For **OCav**, distances of 7.3 ± 0.5 and 11.4 ± 0.3 Å are obtained on average for **d1** and **d2**, respectively, when considering the simulation period 250–450 ns in which no switch between **d1** and **d2** occurred (Figure 3). Taking into account the van der Waals radii of the carbon atoms on both ends of each distance (2 × 1.5 Å), we can assume approximate dimensions of 4.3 Å and 8.4 Å for the interior of **OCav** (Figure 4). Similarly, the depth of the cavitand can be estimated to be 6.3 Å from the distance between the oxygen atom at the top and the carbon atom at the bottom of the cavity, **d3**, minus the van der Waals radii, as illustrated in Figure 4.

**Figure 4:**
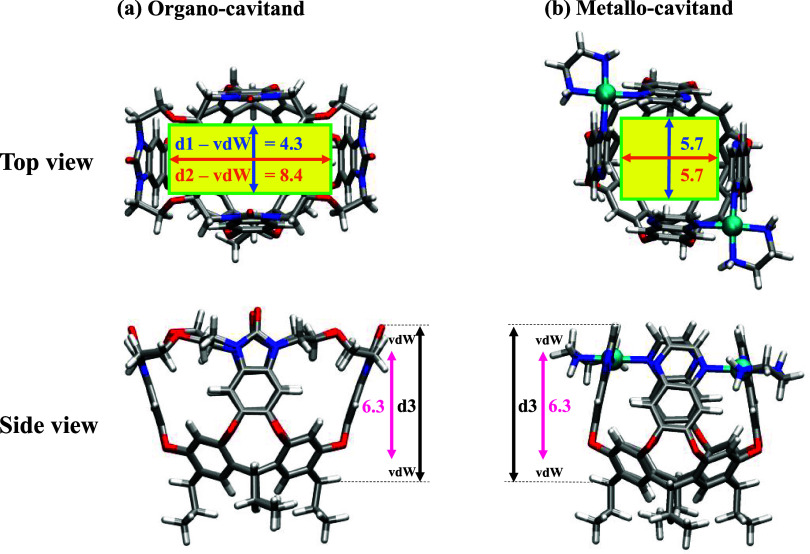
Approximate dimensions of the interior space of the cavitands. Distances are given in angstroms.

For the **MCav**, an average distance of 8.7 ± 0.4 Å is obtained from the simulations for both **d1** and **d2**. Accordingly, we may also assume that approximate dimension is 5.7 Å, after removing the van der Waals radii at both ends of each distance. The depth of this cavitand is also estimated to be 6.3 Å (Figure 4). Interestingly, the dimensions obtained from geometry optimizations of the empty cavitands (without any explicit solvent molecules) using DFT calculations are quite similar to the ones discussed here (see Supporting Information, Figure S11).

From the MD simulations, we also estimate the average volumes of the interior space of the two cavitands, which were found to be 226 ± 21 Å^3^ for **OCav** and 206 ± 13 Å^3^ for **MCav**. These values are very similar to the volumes estimated on the basis of the averaged dimensions discussed above: 8.4 × 4.3 × 6.3 = 228 Å^3^ for **OCav**, and 5.7 × 5.7 × 6.3 = 219 Å^3^ for **MCav**. These estimates show that, although the shapes of the cavities are quite different, the overall volumes are comparable for the two cavitands.

To further characterize the cavitands in solution, we analyze the number of solvent molecules in each cavitand during the simulations. For **OCav**, the number of water molecules fluctuates between 0–7, while for **MCav** the number is between 0–5 (Figures 5 and S12), reflecting the flexibility of the cavitands. On average, **OCav** and **MCav** contain 2.2 and 1.9 solvent molecules, respectively. The fact that these two values are similar is consistent with their average volumes being very similar, as discussed above. Furthermore, the simulations show that solvent molecules can be completely excluded from the interior space of both cavitands for a significant fraction of the time (Figure S12) by two opposite walls moving closer to each other, a very unusual feature.

**Figure 5:**
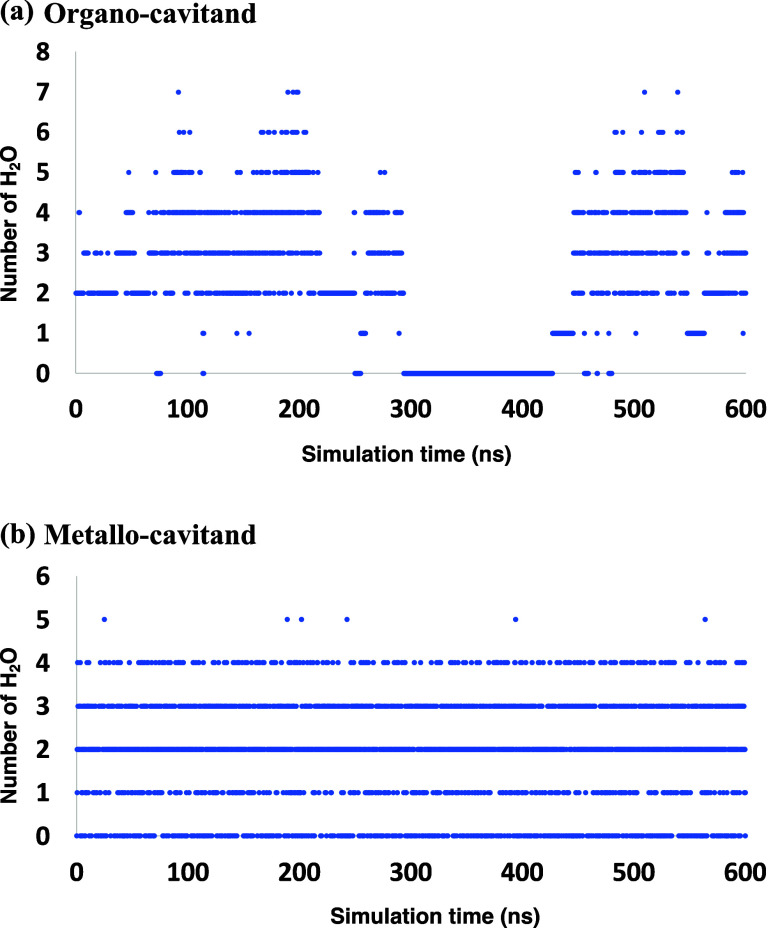
Number of water molecules inside the cavitands during MD simulations.

In summary, the MD simulations show that both cavitands are dynamic and quite flexible. They can open and close their rims and vary their internal volumes to accommodate a number of solvent molecules.

### 3.2 Characterization of Host–Guest Complexes

Next, we focused on the binding of the different xylene isomers, performing 200 ns MD simulations to examine the dynamics of each host–guest complex. The starting structures for these simulations involved the guest molecules already bound to the cavitands.

The simulations show first that for **MCav**, xylene is the only guest in the cavitand and no water molecules can fit inside along with any of the isomers. For **OCav**, a water molecule was observed along with *m*-xylene and *p*-xylene in negligible 0.2 and 0.4% lengths of the time, respectively, while for *o*-xylene, no water could be observed inside the cavitand (see Supporting Information, Figures S13 and S14).

One striking observation from all MD simulations with **OCav** is that both guest and host *undergo extensive motion in the complexed state*. For example, as displayed in Figure 6, the fluctuations of the dimensions of the cavitand (the **d1** and **d2** distances described above) for *o*-xylene inside **OCav** (**o-xylene⊂OCav**) show how the walls are mobile and how the rectangular shape can flip by interchanging the short and long distances between the walls, in a similar fashion as was the case in the absence of the xylene guest (Figure 3). Here, we note that during the simulations the methyl groups of the o-xylene guest can point in any direction inside the cavitand (Figure 6). The guest can also undergo rotational movements, around the axis perpendicular to the benzene plane and around an axis parallel with the vertical axis of the cavitand. No horizontal flipping of the guest could be observed in the simulation, however, as the plane of the xylene cannot be perpendicular to the cavitand walls.

**Figure 6:**
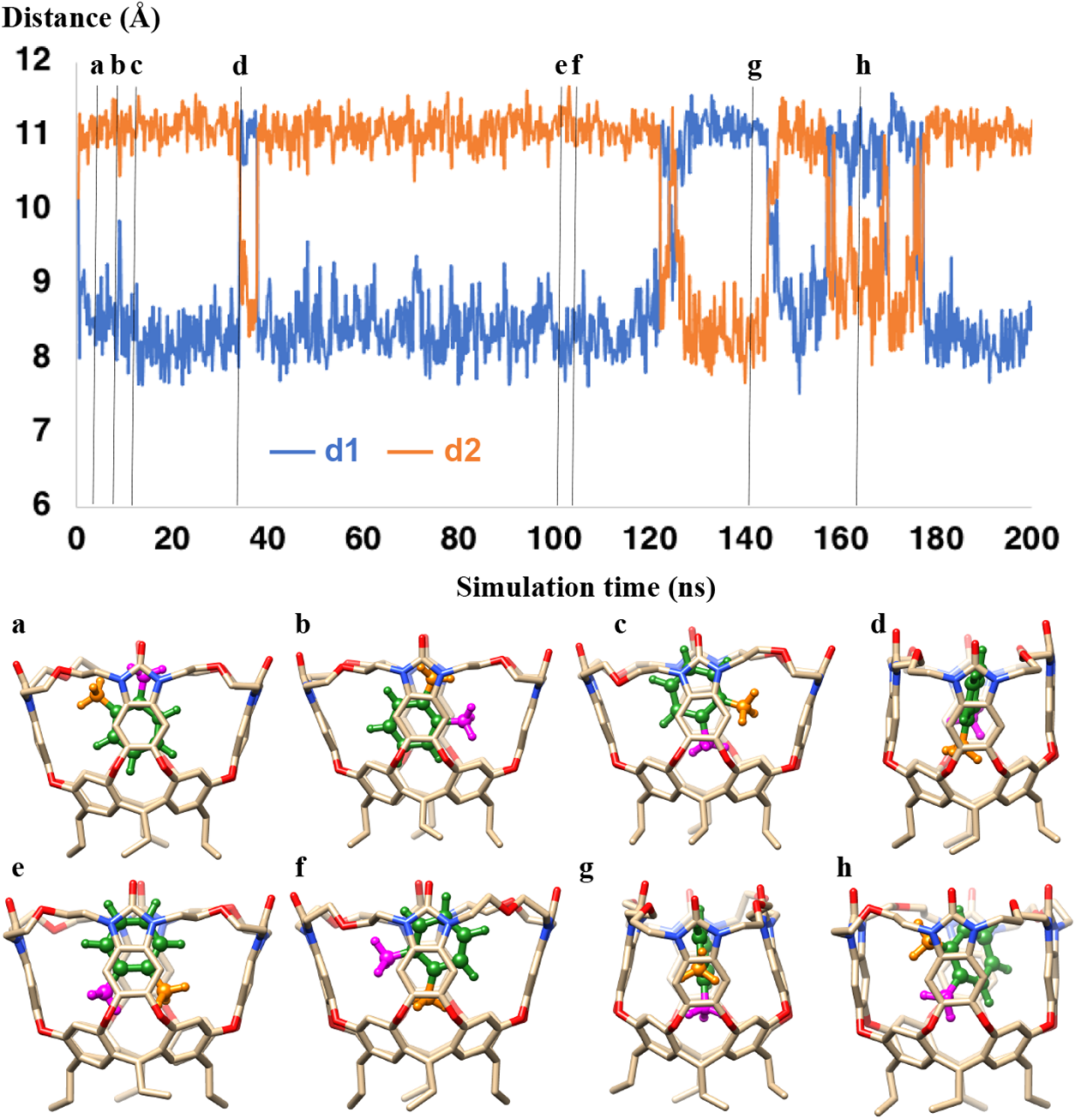
Molecular dynamics simulations of **o-xylene⊂OCav**. The upper panel shows the movement of the walls of the cavitand as described by the **d1** and **d2** distances. Geometries of representative snapshots are shown in the lower panel. The two methyl groups of the xylene are colored differently for better tracking of the motion of the guest inside the cavitand.

Similar motions can be observed for both cavitand and guest in the case of *m*-xylene inside **OCav**, **m-xylene⊂OCav** (see Supporting Information, Figure S15). However, for this guest no snapshot in the simulation could be observed in which both methyl groups point toward the bottom of the cavitand at the same time. In the case of *p*-xylene inside **OCav** (*p* **-xylene⊂OCav**), rotations about the same axes could be observed, and the xylene can be oriented both vertically and horizontally inside the cavitand (see Supporting Information, Figure S16).

For **MCav**, a similar analysis shows less flexibility as compared to **OCav**, displaying an analogous behavior as in the absence of guests. Interestingly, in contrast to **OCav**, in the simulations with **MCav** full rotation around the axis perpendicular to the benzene plane could not be observed for any of the guests. For *o*-xylene inside **MCav** (**o-xylene⊂MCav**), the simulation was started with both methyl groups pointing toward the bottom of the cavitand and it was observed that the guest remained in this orientation (Figure 7), showing only some restricted tilting and rotational movements, unlike the case of **o-xylene⊂OCav**. The benzene plane of the guest remains oriented along the diagonal defined by the two palladium atoms. In the cases of *m*-xylene inside **MCav** (**m-xylene⊂MCav**) and *p*-xylene inside **MCav** (*p* **-xylene⊂MCav**), on the other hand, the plane of the xylene was found during the simulations to orient along both diagonals between the cavitand walls (see Supporting Information, Figures S17 and S18).

**Figure 7:**
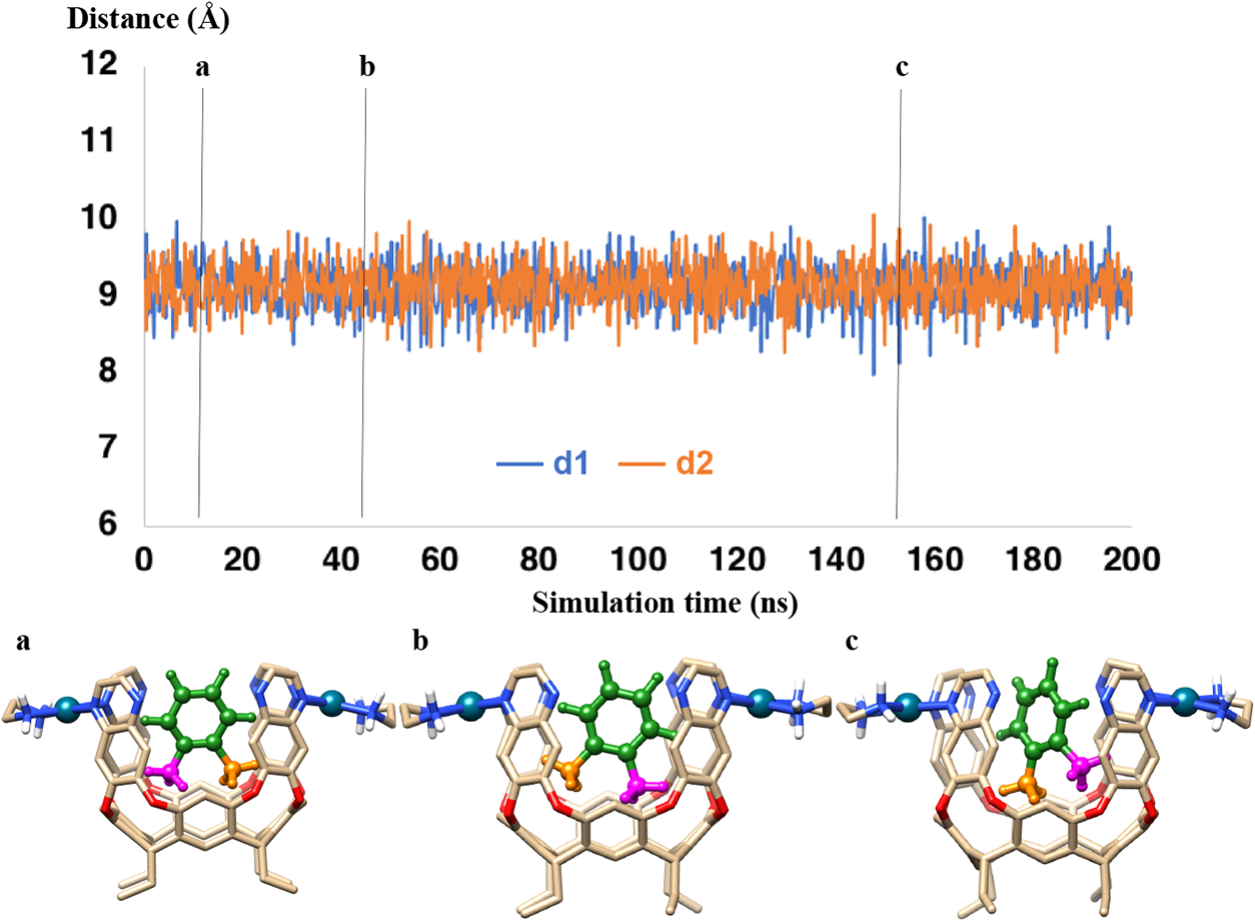
Molecular dynamics simulations of **o-xylene⊂MCav**. The upper panel shows the movement of the walls of the cavitand as described by the **d1** and **d2** distances. Geometries of representative snapshots are shown in the lower panel.

### 3.3 Calculation of Binding Free Energies

Next, different binding modes for each xylene guest were considered for the DFT geometry optimizations by a combination of the most populated structures generated from the classical MD simulations and other possible initial structures constructed manually.

In order to investigate the binding selectivity determined by the competitive binding experiments, we calculated the relative Gibbs binding energies of the xylene isomers inside the cavitands using the following equilibrium

A⊂Cav+B⇌B⊂Cav+A

Very gratifyingly, the experimental trend in the binding energies for the organo-cavitand is well-reproduced by the calculations, with *o*-xylene being the best binder, followed by *m*-xylene (+2.3 kcal/mol) and *p*-xylene (+4.5 kcal/mol). As discussed in the Introduction, the latter was experimentally not taken up by the organo-cavitand.^8^ The optimized structures of the lowest-energy binding modes of each xylene isomer are shown in Figure 8, while other binding modes with higher energies are given in the Supporting Information (Figure S19). In the lowest-energy binding mode of *o*-xylene, one of the methyl groups points toward the bottom of the cavity, while the other one points toward one of the walls (Figure 8a). In contrast, for both *m*-xylene and *p*-xylene, the guest binds with one methyl pointing toward the opening of the cavitand, as shown in Figure 8. Interestingly, for *p*-xylene, another binding mode with both methyl groups pointing toward the walls was found to have the same energy (see Supporting Information, Figures S20 and S21).

**Figure 8:**
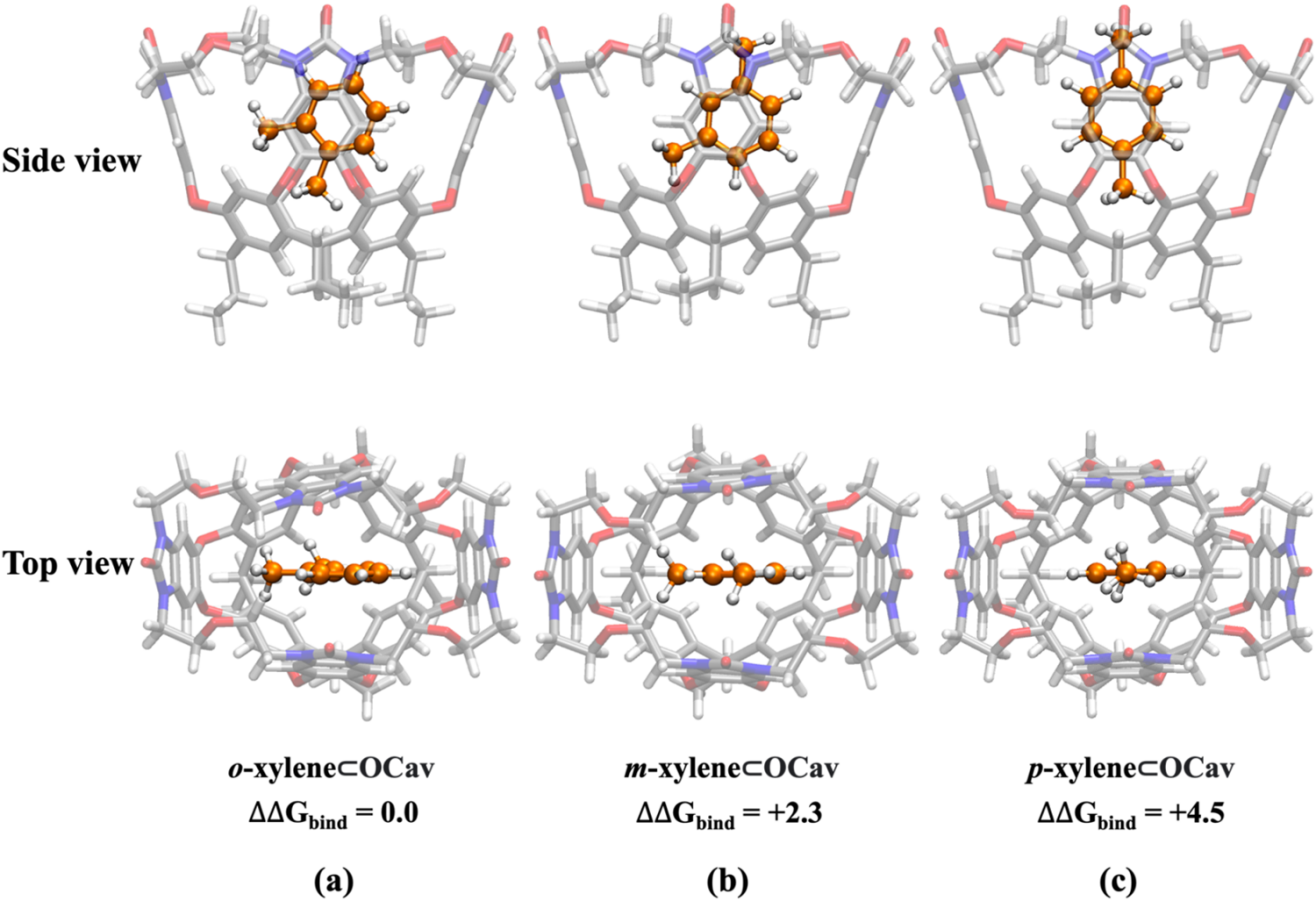
Optimized geometries of the lowest-energy binding modes of xylene isomers in the organo-cavitand. Relative Gibbs binding energies are given in kcal/mol.

The experimental binding trend is also well-reproduced for the metallo-cavitand, with *p*-xylene being the best binder, followed by *o*-xylene (+1.4 kcal/mol) and *m*-xylene (+3.3 kcal/mol).^9^ The lowest-energy binding mode of each xylene isomer is shown in Figure 9, and other binding modes with higher energies are given in the Supporting Information (Figures S22–S24). The xylenes bind with the benzene ring along one of the diagonals between the walls in the lowest-energy binding modes. Note that the two diagonals are not the same, because one of them is defined by the two palladium bridges, while the other one is along the openings between the walls. The *p*-xylene binds with the benzene ring along the diagonal with the openings. In contrast, both *o*-xylene and *m*-xylene bind with the benzene ring along the other diagonal. However, binding of *p*-xylene in the same diagonal as *o*- and *m*-xylene is calculated to be only 0.7 kcal/mol higher (see Supporting Information, Figure S24). Both *p*- and *m*-xylene bind with one methyl group pointing up to toward the opening of the cavitand, while *o*-xylene binds with both methyl groups pointing down toward the bottom of the cavity.

**Figure 9:**
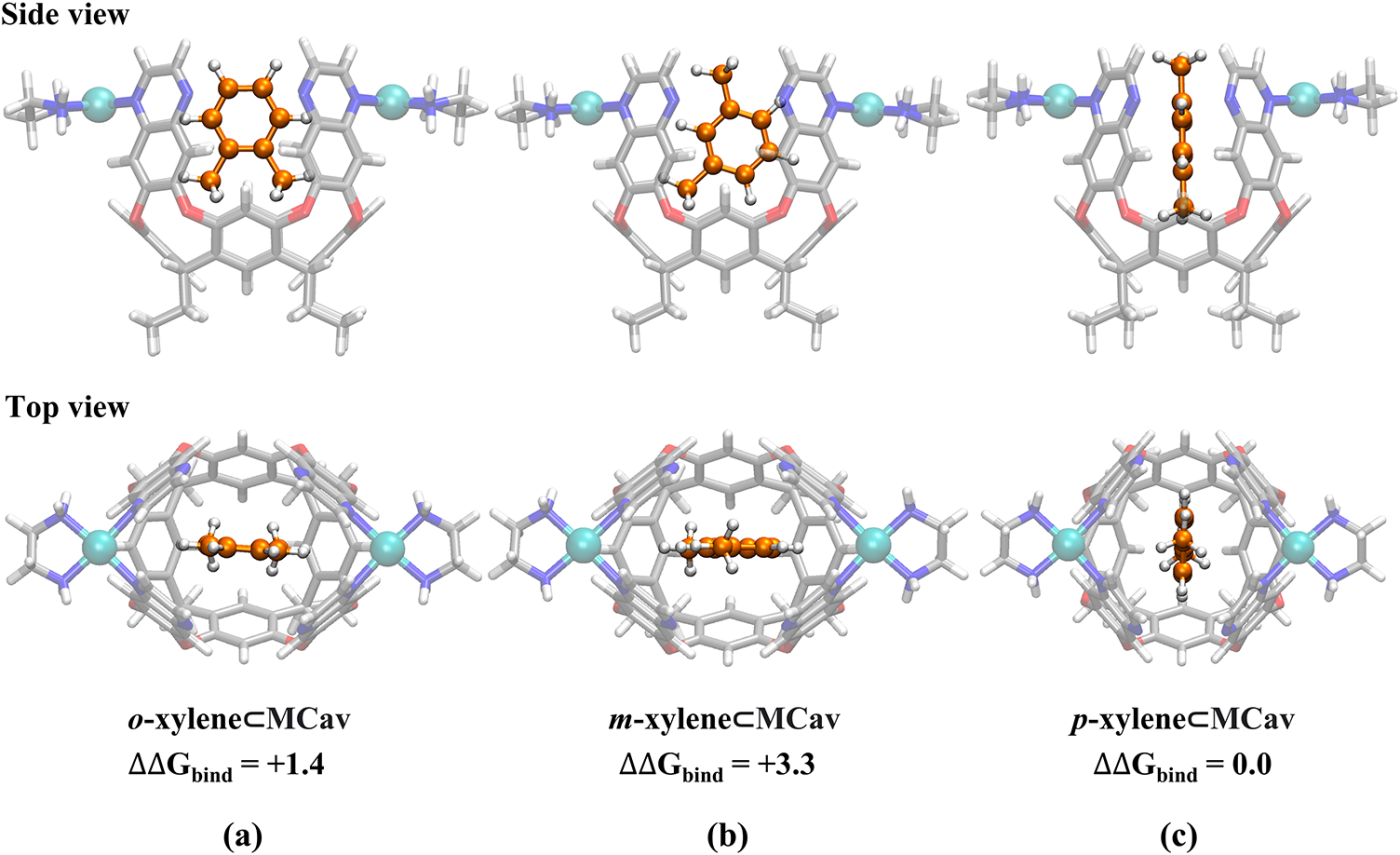
Optimized geometries of the lowest-energy binding mode of xylene isomers in the metallo-cavitand. Relative Gibbs binding energies are given in kcal/mol.

These results show thus that the adopted computational protocol, with MD simulations followed geometry optimizations and energy calculations using DFT, is capable of reproducing the observed experimental selectivity trends very well. In a recent study on binding of xylenes in cucurbit[7]­uril, DFT calculations were also used to model binding modes for the three isomers; these calculations also reproduced binding trends in line with the experimental findings.^16^

## 4 Conclusions

A combination of molecular dynamics simulations and density functional theory calculations were used to investigate the binding of xylene isomers to two water-soluble resorcin[4]­arene-based cavitands, **OCav** and **MCav** (Figure 2). We first characterized the two cavitands in explicit water solvent using classical molecular dynamics simulations, which showed that both cavitands are flexible and can open and close the rim, varying their internal volumes to accommodate different numbers of solvent molecules.

Next, MD simulations were employed to analyze the dynamics of the host–guest complexes. These simulations showed that the cavitands are as dynamic and flexible as in the absence of the guests. These studies also demonstrated that the xylene guests are able to rotate in the complexed state, maintaining attractive contacts with the cavitand.

Finally, we used density functional theory to optimize the geometries of the host–guest complexes and to evaluate their relative binding free energies. The DFT calculations yielded very good agreement with the experimental observations in terms of trends in binding free energies.

The results demonstrate that the adopted computational protocol, although rather simple involving classical MD simulations for conformational sampling followed by standard DFT geometry optimizations and energy calculations, can indeed be a very powerful tool for the study of binding and selectivity in host–guest systems.

Recognition is driven by weak, intermolecular forces acting between molecules of complementary size, shape and chemical surface. The present work shows that both guest and host undergo extensive motion in the complexed state. This is at odds with the reigning paradigm of “preorganization” in molecular recognition. The concepts of “host” and “guest” invoke stationary and mobile entities, respectively, and the search for guest selectivity and high affinity has historically been interpreted as building rigid structures such as spherands and carcerands. Here, we have mobile xylene guests dancing in accommodating cavitands. And yet there is selectivity!

## Supplementary Material



## Data Availability

The data underlying this study are available in the published article and its Supporting Information.

## Notes

Analysis of MD simulations, optimized geometries, empty cavitands, and various host–guest complexes, calculated absolute energies and energy corrections, and Cartesian coordinates of optimized geometries (PDF)

## ∥

Departament de Química and Centro de Innovación en Química Avanzada (ORFEO–CINQA), Universitat Autònoma de Barcelona, 08193 Cerdanyola del Valles, Barcelona, Catalonia, Spai

## References

[1] Moran J. R., Karbach S., Cram D. J. (1982). Cavitands: Synthetic molecular vessels. J. Am. Chem. Soc..

[2] Dalcanale E., Soncini P., Bacchilega G., Ugozzoli F. (1989). Selective Complexation
of Neutral Molecules in Organic Solvents. Host-Guest Complexes and
Cavitates between Cavitands and Aromatic Compounds. J. Chem. Soc., Chem. Commun..

[3] Cram D. J., Choi H. J., Bryant J. A., Knobler C. B. (1992). Host-guest Complexation
Solvophobic and Entropic Driving Forces for Forming Velcraplexes,
Which Are 4-fold, Lock-Key Dimers in Organic Media. J. Am. Chem. Soc..

[4] Rudkevich D. M., Hilmersson G., Rebek J. (1997). Intramolecular Hydrogen Bonding Controls
the Exchange Rates of Guests in a Cavitand. J. Am. Chem. Soc..

[5] Gibb C. L. D., Gibb B. C. (2004). Well Defined, Organic
Nano-Environments in Water: The
Hydrophobic Effect Drives a Capsular Assembly. J. Am. Chem. Soc..

[6] Fankhauser D., Kolarski D., Grüning W. R., Diederich F. (2014). Resorcin­[4]­arene-Based
Molecular Baskets and Water-Soluble Container Molecules: Synthesis
and ^1^H NMR Host-Guest Complexation Studies. Eur. J. Org. Chem..

[7] Rahman F.-U., Li Y.-S., Petsalakis I. D., Theodorakopoulos G., Rebek J., Yu Y. (2019). Recognition with Metallo Cavitands. Proc. Natl. Acad. Sci. U.S.A..

[8] Yang J.-M., Chen Y.-Q., Yu Y., Ballester P., Rebek J. (2021). Rigidified Cavitand Hosts in Water:
Bent Guests, Shape Selectivity,
and Encapsulation. J. Am. Chem. Soc..

[9] Rahman F.-U., Yang J.-M., Wan Y.-H., Zhang H.-B., Petsalakis I. D., Theodorakopoulos G., Rebek J., Yu Y. (2020). Binding Selectivity
and Separation of *p*-Functionalized Toluenes with
a Metallo-Cavitand in Water. Chem. Commun..

[10] Yang Y., Bai P., Guo X. (2017). Separation
of Xylene Isomers: A Review of Recent Advances
in Materials. Ind. Eng. Chem. Res..

[11] Sholl D. S., Lively R. P. (2016). Seven Chemical Separations
to Change the World. Nature.

[12] Gonzalez M. I., Kapelewski M. T., Bloch E. D., Milner P. J., Reed D. A., Hudson M. R., Mason J. A., Barin G., Brown C. M., Long J. R. (2018). Separation
of Xylene Isomers through Multiple Metal
Site Interactions in Metal-Organic Frameworks. J. Am. Chem. Soc..

[13] Jie K., Liu M., Zhou Y., Little M. A., Pulido A., Chong S. Y., Stephenson A., Hughes A. R., Sakakibara F., Ogoshi T., Blanc F., Day G. M., Huang F., Cooper A. I. (2018). Near-Ideal Xylene Selectivity in Adaptive Molecular
Pillar­[n]­arene Crystals. J. Am. Chem. Soc..

[14] Huang J., Han X., Yang S., Cao Y., Yuan C., Liu Y., Wang J., Cui Y. (2019). Microporous
3D Covalent Organic Frameworks
for Liquid Chromatographic Separation of Xylene Isomers and Ethylbenzene. J. Am. Chem. Soc..

[15] Wang S.-Q., Mukherjee S., Patyk-Kaźmierczak E., Darwish S., Bajpai A., Yang Q.-Y., Zaworotko M. J. (2019). Highly
Selective, High-Capacity Separation of o-Xylene from C8 Aromatics
by a Switching Adsorbent Layered Material. Angew.
Chem., Int. Ed..

[16] Zhang G., Emwas A.-H., Shahul Hameed U. F., Arold S. T., Yang P., Chen A., Xiang J.-F., Khashab N. M. (2020). Shape-Induced Selective
Separation of Ortho-Substituted Benzene Isomers Enabled by Cucurbit[7]­uril
Host Macrocycles. Chem.

[17] Qiao Z., Yan Y., Tang Y., Liang H., Jiang J. (2021). Metal–Organic
Frameworks for Xylene Separation: From Computational Screening to
Machine Learning. J. Phys. Chem. C.

[18] Xu M., Tang W.-Q., Meng S.-S., Gu Z.-Y. (2025). Metal–Organic
Frameworks for the Separation of Xylene Isomers. Chem. Soc. Rev..

[19] Norjmaa G., Rebek J., Himo F. (2024). Modeling Amine Methylation in Methyl
Ester Cavitand. Chem.—Eur. J..

[20] Becke A. D. (1993). Density-Functional
Thermochemistry. III. The Role of Exact Exchange. J. Chem. Phys..

[21] Becke A. D. (1988). Density-Functional
Exchange-Energy Approximation with Correct Asymptotic Behavior. Phys. Rev. A.

[22] Lee C., Yang W., Parr R. G. (1988). Development of the Colle-Salvetti
Correlation-Energy Formula into a Functional of the Electron Density. Phys. Rev. B.

[23] Grimme S., Antony J., Ehrlich S., Krieg H. (2010). A Consistent and Accurate
Ab Initio Parametrization of Density Functional Dispersion Correction
(DFT-D) for the 94 Elements H-Pu. J. Chem. Phys..

[24] Grimme S., Ehrlich S., Goerigk L. (2011). Effect of the Damping Function in
Dispersion Corrected Density Functional Theory. J. Comput. Chem..

[25] Becke A. D., Johnson E. R. (2005). A Density-Functional Model of the Dispersion Interaction. J. Chem. Phys..

[26] Frisch, M. J. ; Trucks, G. W. ; Schlegel, H. B. ; Scuseria, G. E. ; Robb, M. A. ; Cheeseman, J. R. ; Scalmani, G. ; Barone, V. ; Petersson, G. A. ; Nakatsuji, H. ; Li, X. ; Caricato, M. ; Marenich, A. V. ; Bloino, J. ; Janesko, B. G. ; Gomperts, R. ; Mennucci, B. ; Hratchian, H. P. ; Ortiz, J. V. ; Izmaylov, A. F. ; Sonnenberg, J. L. ; Williams-Young, D. ; Ding, F. ; Lipparini, F. ; Egidi, F. ; Goings, J. ; Peng, B. ; Petrone, A. ; Henderson, T. ; Ranasinghe, D. ; Zakrzewski, V. G. ; Gao, J. ; Rega, N. ; Zheng, G. ; Liang, W. ; Hada, M. ; Ehara, M. ; Toyota, K. ; Fukuda, R. ; Hasegawa, J. ; Ishida, M. ; Nakajima, T. ; Honda, Y. ; Kitao, O. ; Nakai, H. ; Vreven, T. ; Throssell, K. ; Montgomery, J. A., Jr. ; Peralta, J. E. ; Ogliaro, F. ; Bearpark, M. J. ; Heyd, J. J. ; Brothers, E. N. ; Kudin, K. N. ; Staroverov, V. N. ; Keith, T. A. ; Kobayashi, R. ; Normand, J. ; Raghavachari, K. ; Rendell, A. P. ; Burant, J. C. ; Iyengar, S. S. ; Tomasi, J. ; Cossi, M. ; Millam, J. M. ; Klene, M. ; Adamo, C. ; Cammi, R. ; Ochterski, J. W. ; Martin, R. L. ; Morokuma, K. ; Farkas, O. ; Foresman, J. B. ; Fox, D. J. Gaussian 16, revision 01,C; Gaussian, Inc.: Wallingford CT, 2016.

[27] Marenich A. V., Cramer C. J., Truhlar D. G. (2009). Universal Solvation Model Based on
Solute Electron Density and on a Continuum Model of the Solvent Defined
by the Bulk Dielectric Constant and Atomic Surface Tensions. J. Phys. Chem. B.

[28] Hay P. J., Wadt W. R. (1985). *Ab initio* Effective Core Potentials
for Molecular Calculations. Potentials for the Transition Metal Atoms
Sc to Hg. J. Chem. Phys..

[29] Wadt W. R., Hay P. J. (1985). *Ab initio* Effective Core Potentials
for Molecular Calculations. Potentials for Main Group Elements Na
to Bi. J. Chem. Phys..

[30] Grimme S. (2012). Supramolecular
Binding Thermodynamics by Dispersion-Corrected Density Functional
Theory. Chem.—Eur. J..

[31] Case, D. A. ; Betz, R. M. ; Cerutti, D. S. ; Cheatham, T. E., III ; Darden, T. A. ; Duke, R. E. ; Giese, T. J. ; Gohlke, H. ; Goetz, A. W. ; Homeyer, N. ; Izadi, S. ; Janowski, P. ; Kaus, J. ; Kovalenko, A. ; Lee, T. S. ; LeGrand, S. ; Li, P. ; Lin, C. ; Luchko, T. ; Luo, R. ; Madej, B. ; Mermelstein, D. ; Merz, K. M. ; Monard, G. ; Nguyen, H. ; Nguyen, H. T. ; Omelyan, I. ; Onufriev, A. ; Roe, D. R. ; Roitberg, A. ; Sagui, C. ; Simmerling, C. L. ; Botello-Smith, W. M. ; Swails, J. ; Walker, R. C. ; Wang, J. ; Wolf, R. M. ; Wu, X. ; Xiao, L. ; Kollman, P. A. AMBER 2016; University of California: San Francisco, 2016.

[32] Wang J., Wolf R. M., Caldwell J. W., Kollman P. A., Case D. A. (2004). Development
and Testing of a General AMBER Force Field. J. Comput. Chem..

[33] Jakalian A., Jack D. B., Bayly C. I. (2002). Fast, Efficient Generation of High-Quality
Atomic Charges. AM1-BCC Model: II. Parameterization and Validation. J. Comput. Chem..

[34] Bayly C. I., Cieplak P., Cornell W., Kollman P. A. (1993). A Well-Behaved Electrostatic
Potential Based Method Using Charge Restraints for Deriving Atomic
Charges: The RESP Model. J. Phys. Chem. A.

[35] Li P., Merz K. M. (2016). MCPB.py: A Python
Based Metal Center Parameter Builder. J. Chem.
Inf. Model..

[36] Wang J., Wang W., Kollman P. A., Case D. A. (2006). Automatic Atom Type
and Bond Type Perception in Molecular Mechanical Calculations. J. Mol. Graph. Model..

[37] Mark P., Nilsson L. (2001). Structure and Dynamics of the TIP3P,
SPC, and SPC/E
Water Models at 298 K. J. Phys. Chem. A.

[38] Faller R., de Pablo J. J. (2002). Constant Pressure
Hybrid Molecular Dynamics–Monte
Carlo Simulations. J. Chem. Phys..

[39] Essmann U., Perera L., Berkowitz M. L., Darden T., Lee H., Pedersen L. G. (1995). A Smooth Particle
Mesh Ewald Method. J. Chem. Phys..

[40] Pettersen E. F., Goddard T. D., Huang C. C., Couch G. S., Greenblatt D. M., Meng E. C., Ferrin T. E. (2004). UCSF Chimera
- A Visualization System
for Exploratory Research and Analysis. J. Comput.
Chem..

