# Supplementary material for: Modeling Binding Selectivity of Xylene Isomers in Resorcin[4]arene-Based Organo- and Metallo-Cavitands
Source: J Org Chem. 2025 Jul 1;90(27):9327–35. doi: 10.1021/acs.joc.5c00471 (PMC12261323; doi:10.1021/acs.joc.5c00471)
Supplement: Supplementary file 1 [file jo5c00471_si_001.pdf]

Supporting Information

Modeling Binding Selectivity of Xylene Isomers in Resorcin[4]arene-Based Organo- and Metallo-Cavitands

Gantulga Norjmaa,[1] Yang Yu,[2] Julius Rebek Jr.,[2,3] Fahmi Himo*,[1]

[1] Department of Chemistry, Arrhenius Laboratory, Stockholm University, SE-106 91 Stockholm, Sweden.
[2] Center for Supramolecular Chemistry and Catalysis and Department of Chemistry, College of Science, Shanghai University, Shanghai 200444, P. R. China.
[3] The Skaggs Institute for Chemical Biology and Department of Chemistry, The Scripps Research Institute, 10550 North Torrey Pines Road, La Jolla, California 92037, United States.

Contents

1. Analysis of MD simulations. S2
2. Shape of cavity inside cavitands. S10
3. Dimensions of the interior space of empty cavitands. S11
4. Number of solvent molecules in cavitands. S12
5. Number of solvent molecules in cavitand-guest complexes. S13
6. MD simulations of cavitand-guest complexes. S14
7. Optimized geometries of cavitand-guest complexes. S18
8. Absolute energies and energy corrections. S24
9. Cartesian coordinates of optimized geometries. S25

1. Analysis of MD simulations.

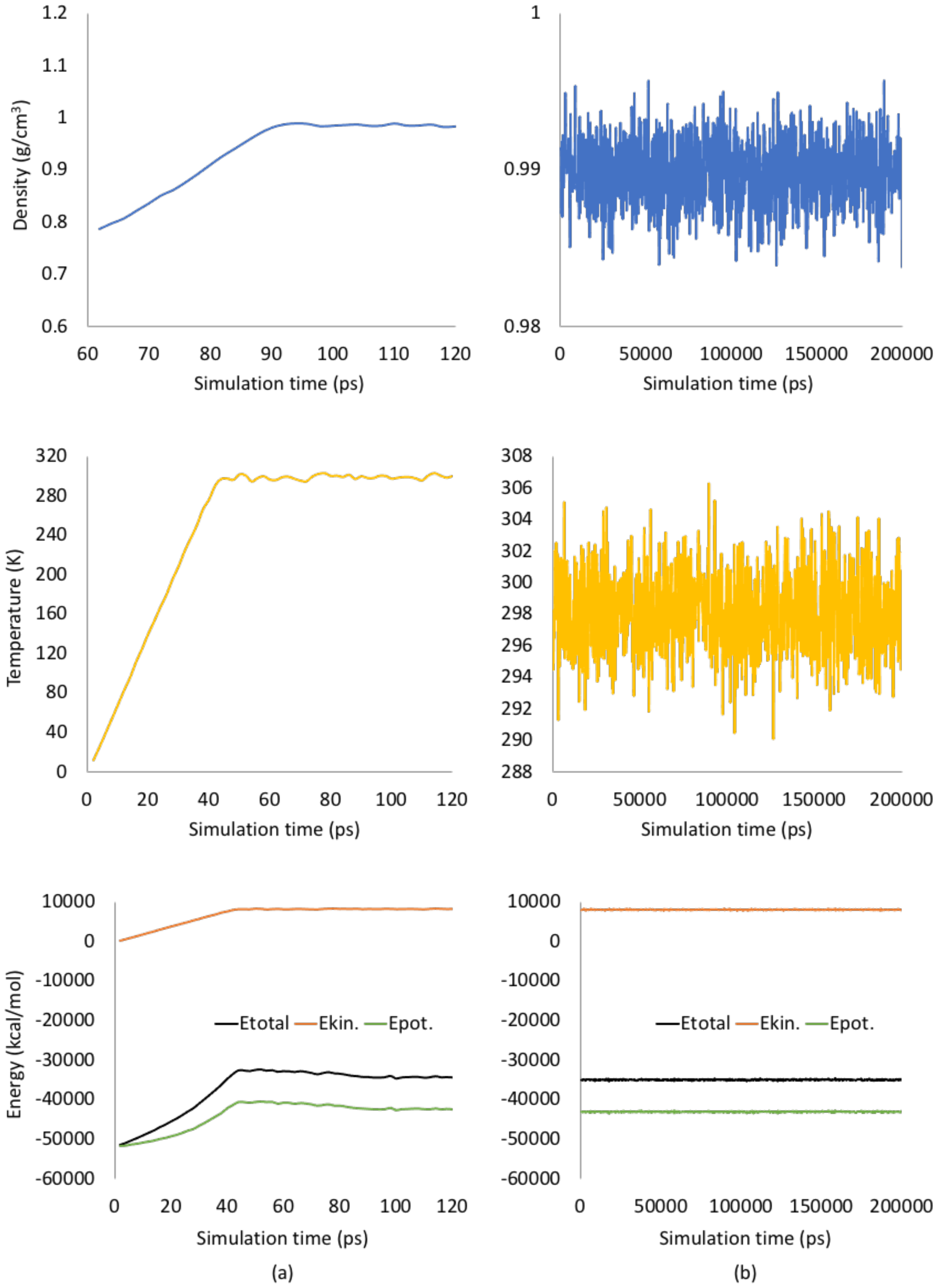

Figure S1. *Analysis of the MD simulation of **o-xylene⊂OCav** in explicit solvent. (a) equilibration period including heating to 298.15 K and (b) NPT production run of 200 ns.*

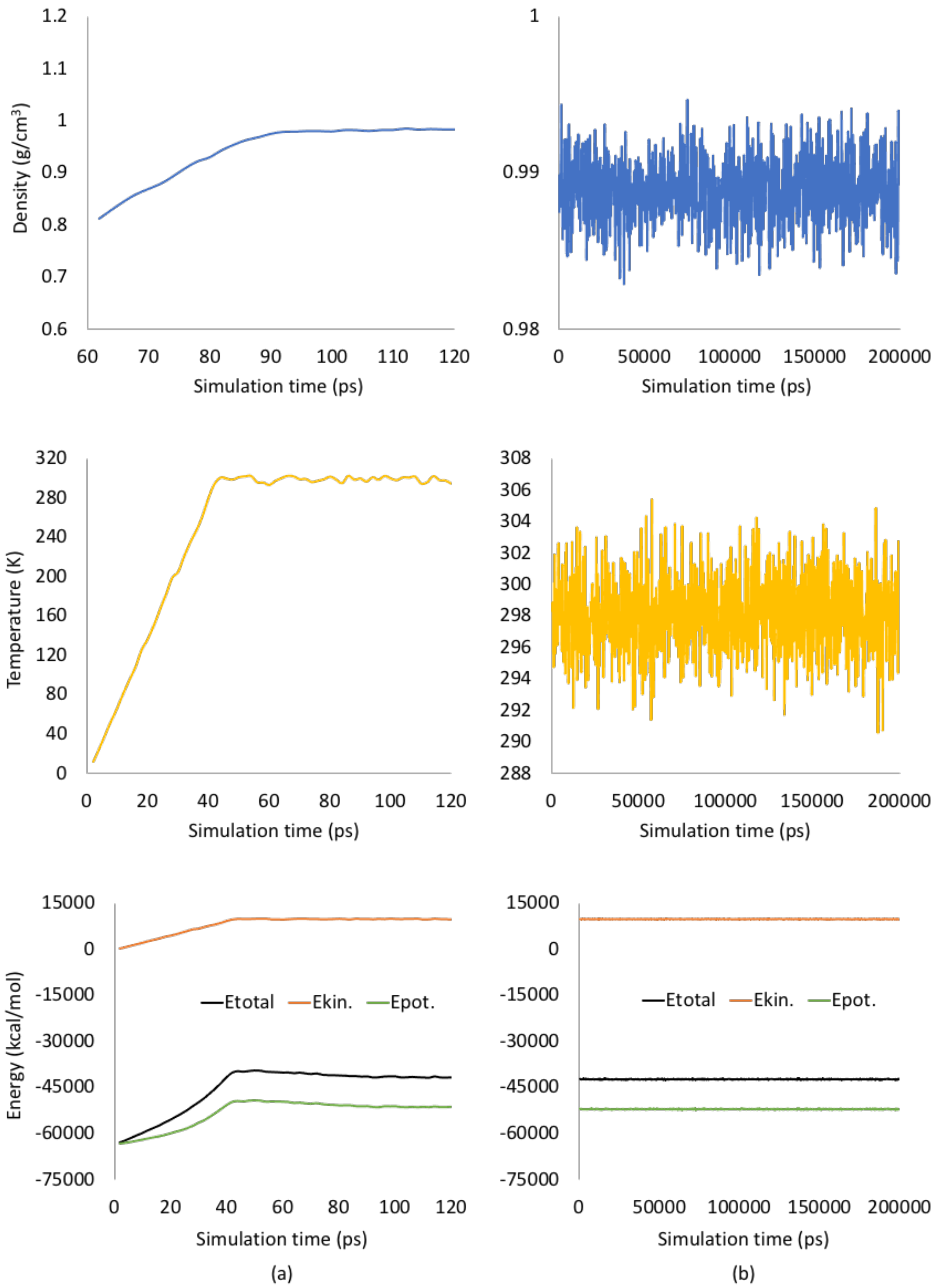

Figure S2. *Analysis of the MD simulation of* ***m-xylene⊂OCav*** *in explicit solvent. (a) equilibration period including heating to 298.15 K and (b) NPT production run of 200 ns.*

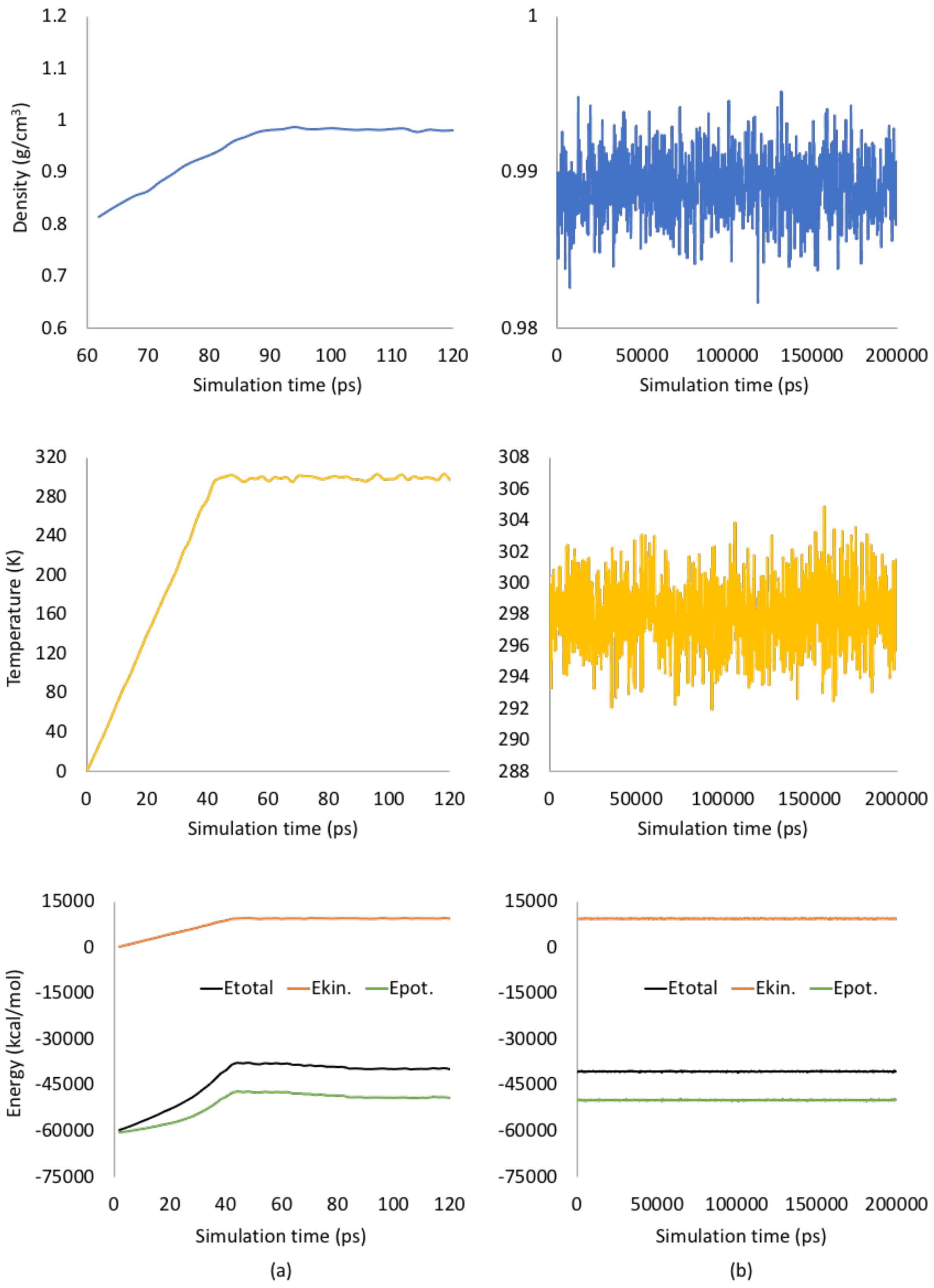

Figure S3. *Analysis of the MD simulation of* ***p-xylene⊂OCav*** *in explicit solvent. (a) equilibration period including heating to 298.15 K and (b) NPT production run of 200 ns.*

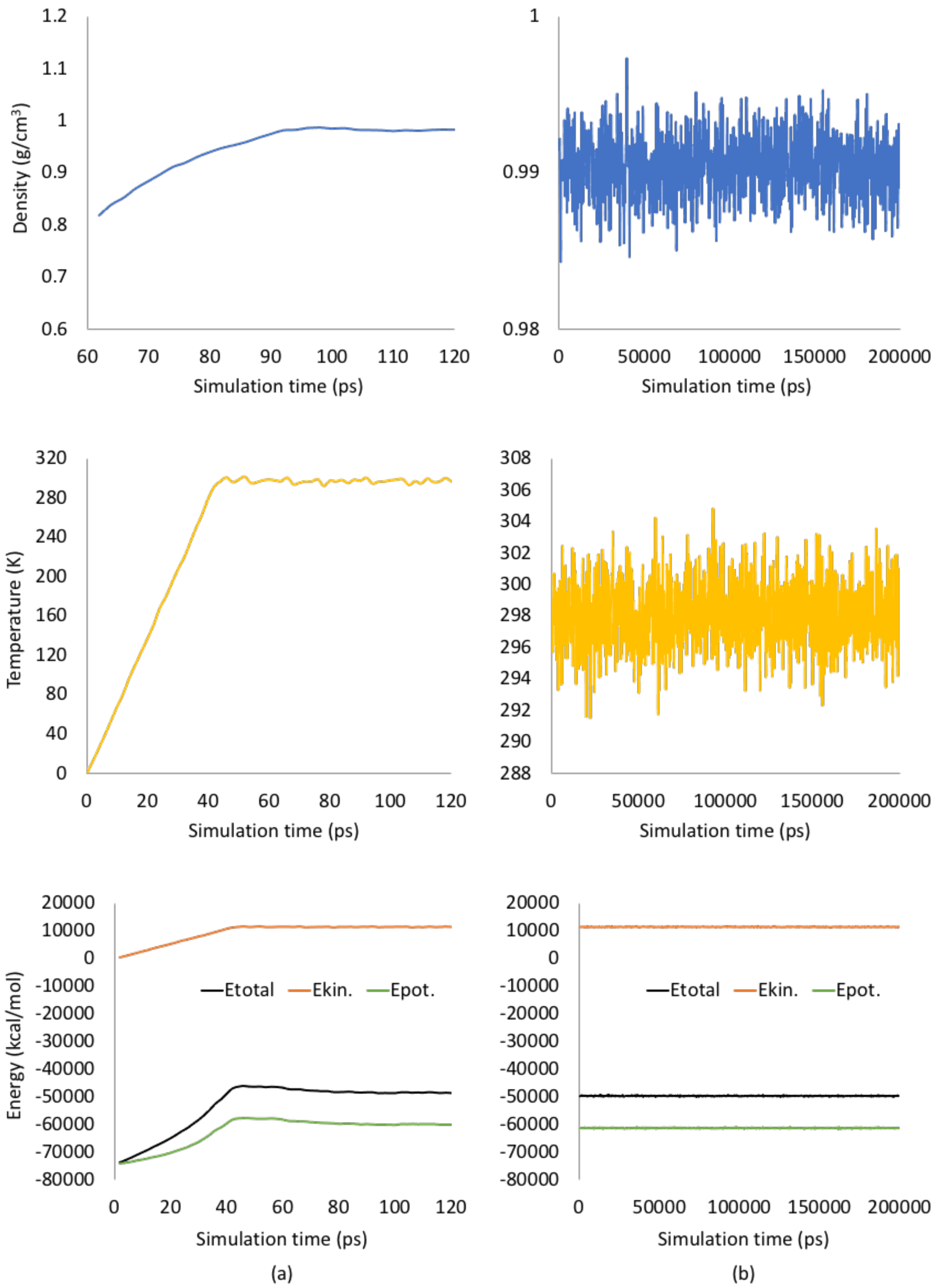

***Figure S4**. Analysis of the MD simulation of **o-xylene⊂MCav** in explicit solvent. (a) equilibration period including heating to 298.15 K and (b) NPT production run of 200 ns.*

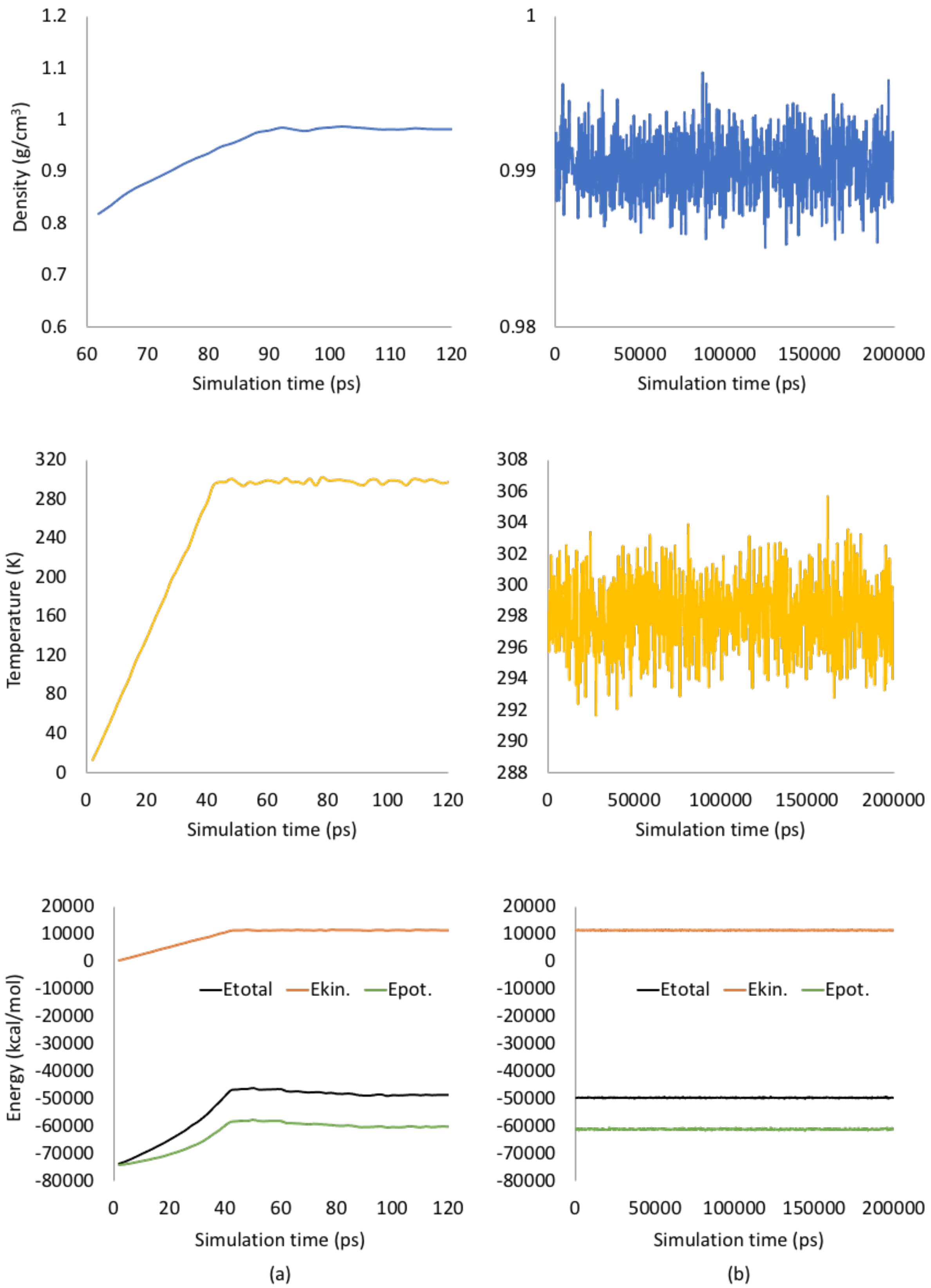

Figure S5. *Analysis of the MD simulation of* ***m-xylene⊂MCav*** *in explicit solvent. (a) equilibration period including heating to 298.15 K and (b) NPT production run of 200 ns.*

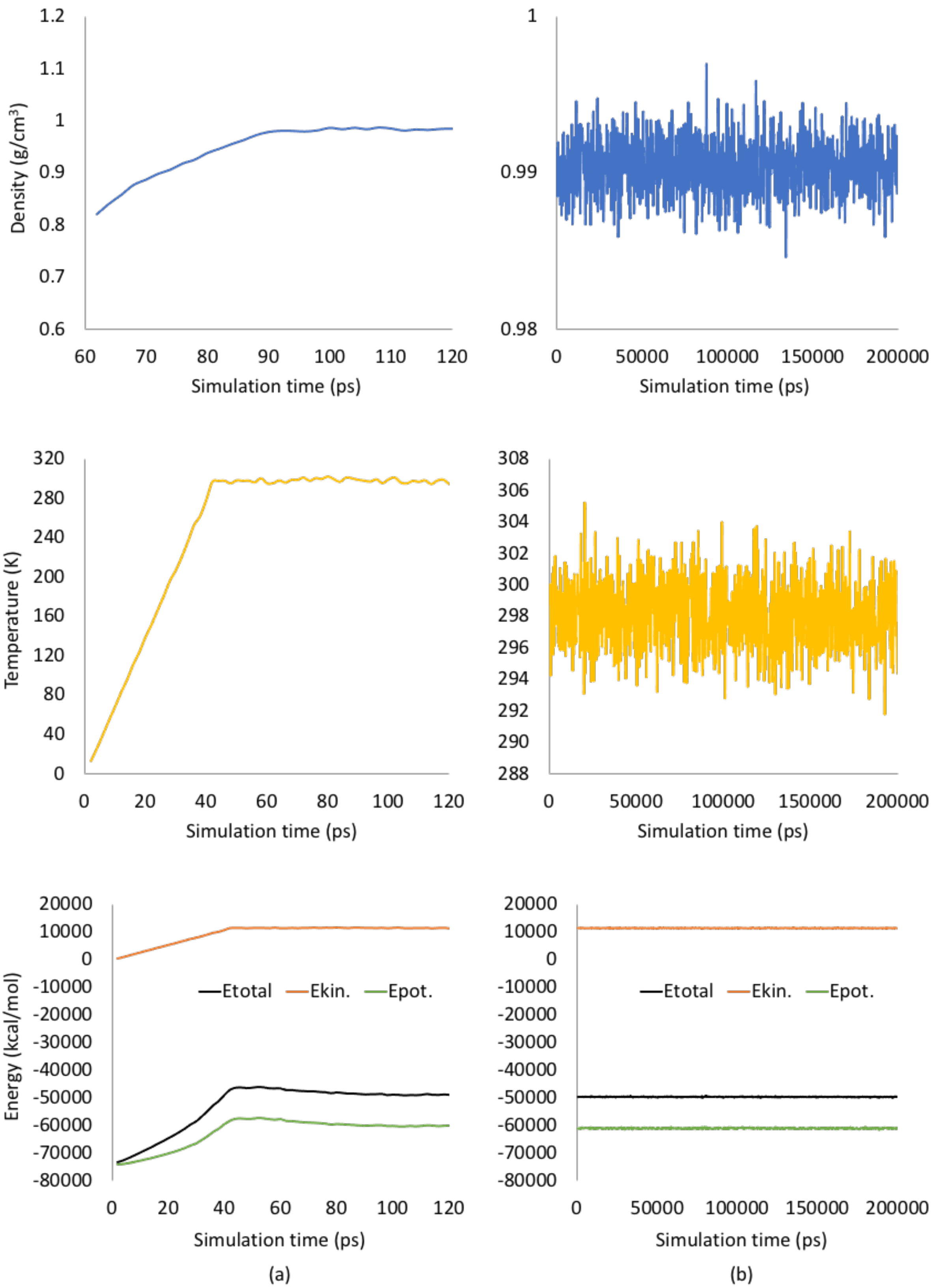

Figure S6. *Analysis of the MD simulation of* ***p-xylene⊂MCav*** *in explicit solvent. (a) equilibration period including heating to 298.15 K and (b) NPT production run of 200 ns.*

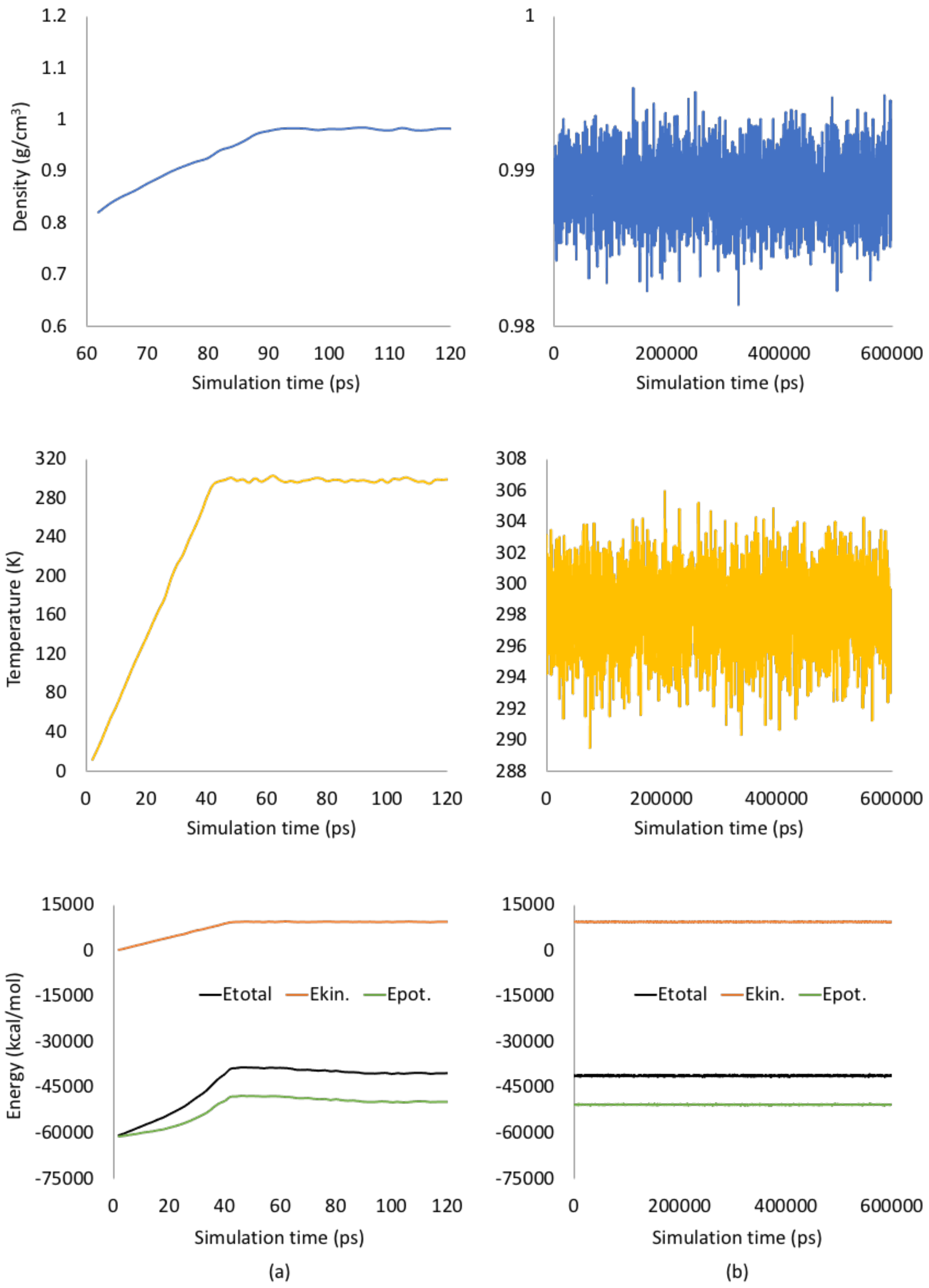

Figure S7*. Analysis of the MD simulation of* ***OCav*** *in explicit solvent. (a) equilibration period including heating to 298.15 K and (b) NPT production run of 600 ns.*

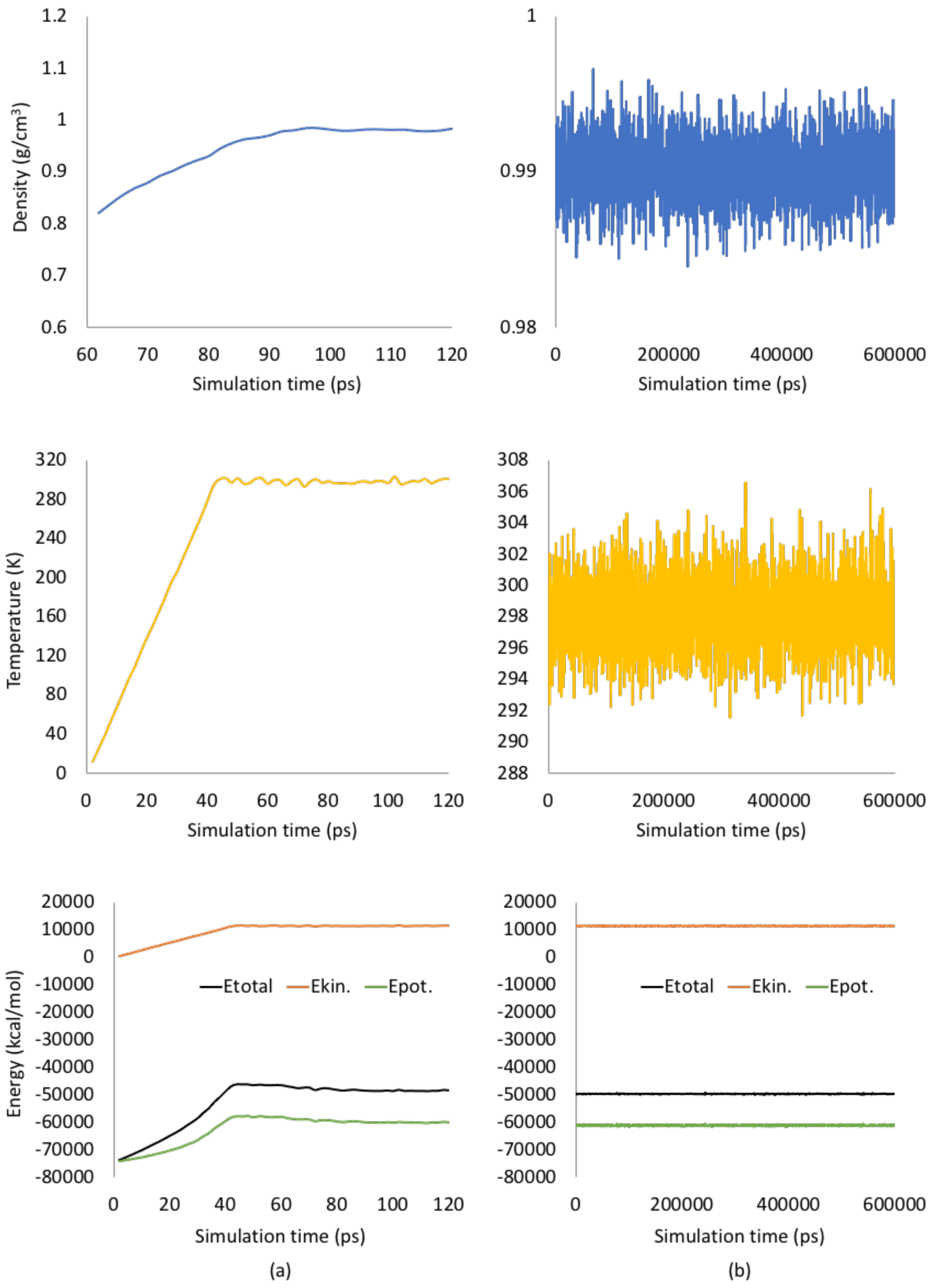

Figure S8. *Analysis of the MD simulation of* ***MCav*** *in explicit solvent. (a) equilibration period including heating to 298.15 K and (b) NPT production run of 600 ns.*

2. Shape of cavity inside cavitands.

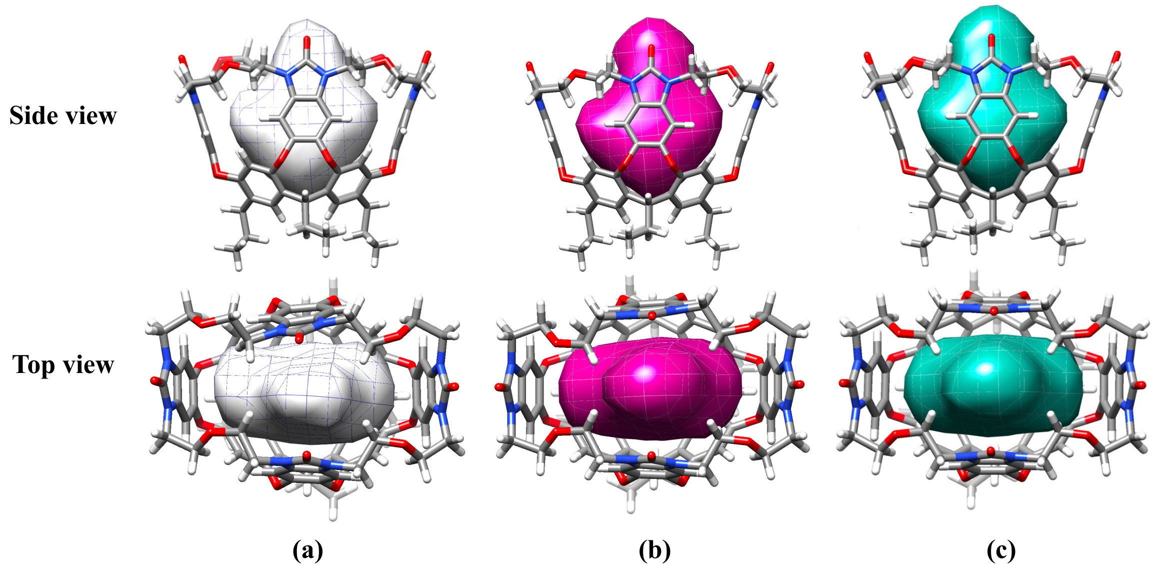

Figure S9. *Cavity inside the* ***OCav*** *calculated based on the cavitand geometry extracted from (a)* ***o-xylene⊂OCav****, (b)* ***m-xylene⊂OCav****, and (c)* ***p-xylene⊂OCav****.*

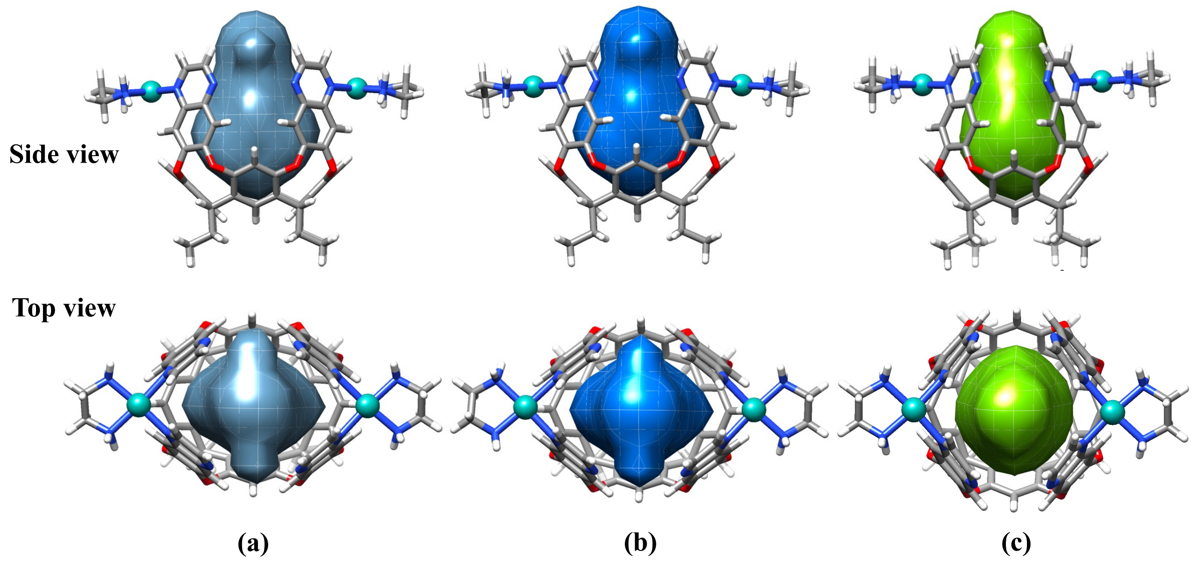

Figure S10. *Cavity inside the* ***MCav*** *calculated based on the cavitand geometry extracted from (a)* ***o-xylene⊂MCav****, (b)* ***m-xylene⊂MCav****, and (c)* ***p-xylene⊂MCav****.*

3. Dimensions of the interior space of empty cavitands.

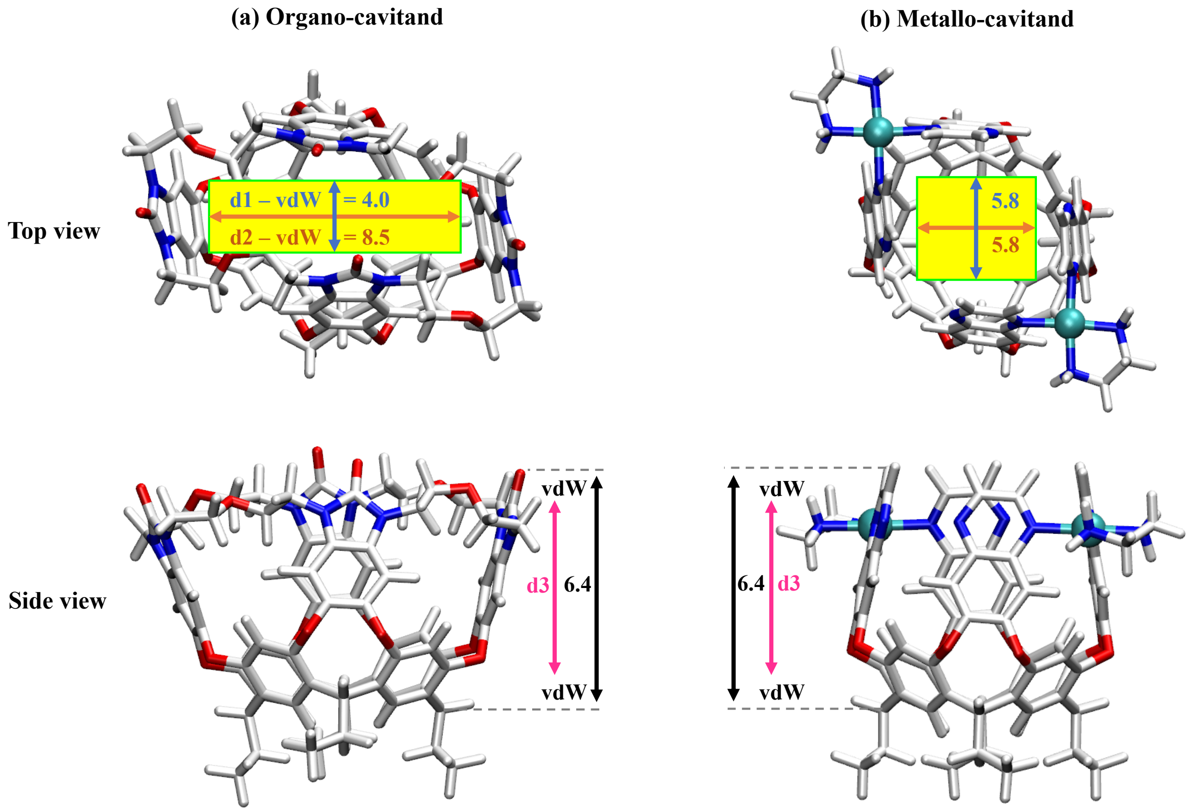

Figure S11. *Approximate dimensions of the interior space of the DFT-optimized empty cavitands (without any explicit solvent molecules). Distances are given in angstrom.*

4. Number of solvent molecules in cavitands.

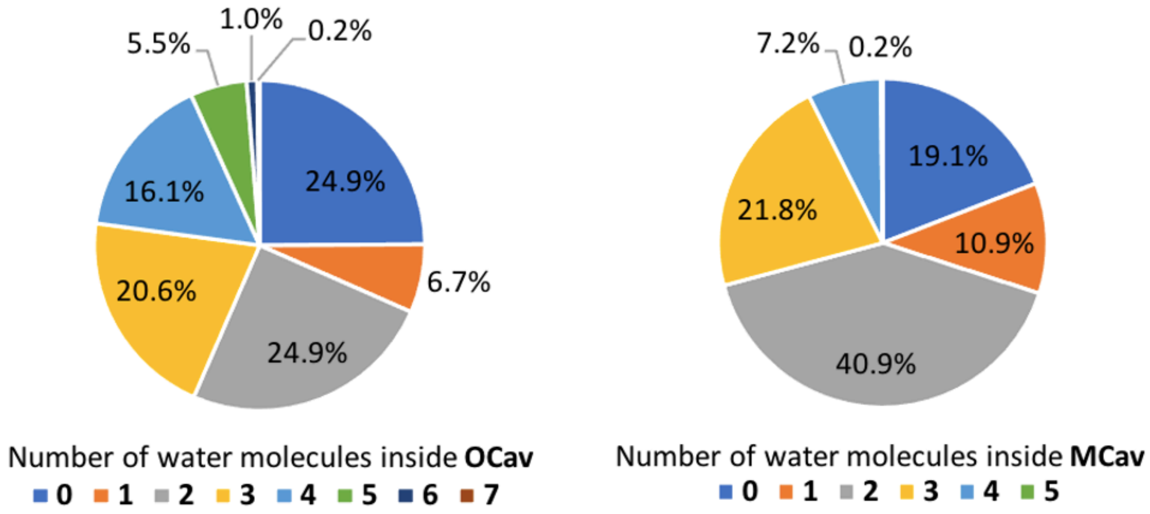

(a) (b)

Figure S12. *Percentage of the number of water molecules inside (a)* ***OCav*** *and (b)* ***MCav*** *during the MD simulation.*

5. Number of solvent molecules in cavitand-guest complexes.

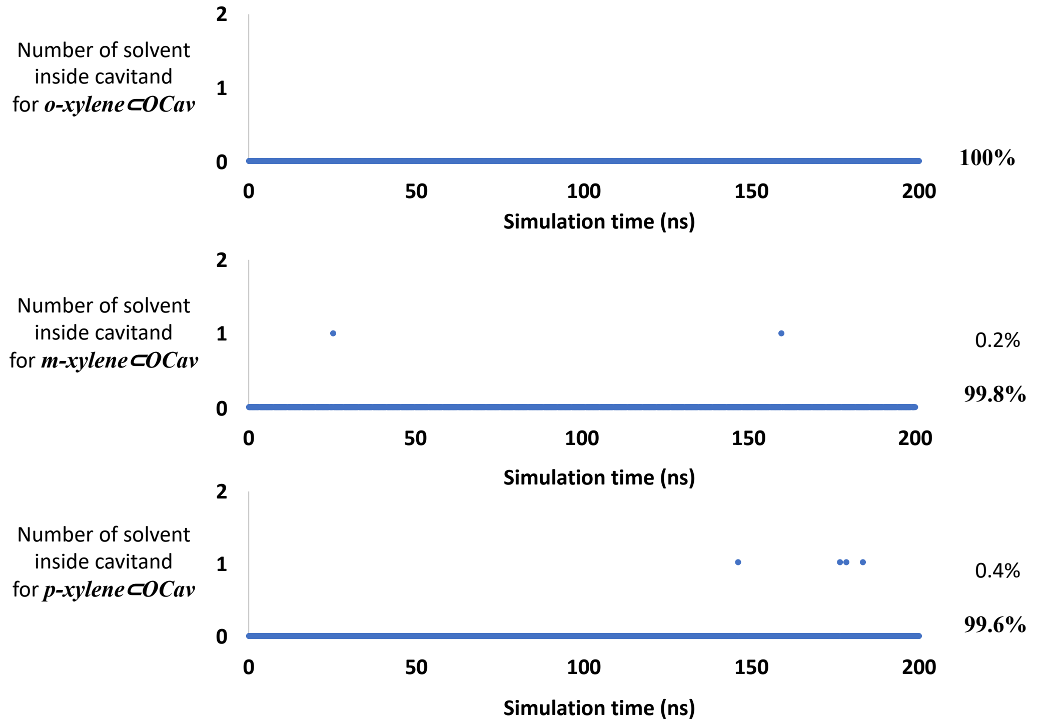

Figure S13. *Number of solvent molecules in* ***OCav*** *during the MD simulations of the cavitand-guest complexes.*

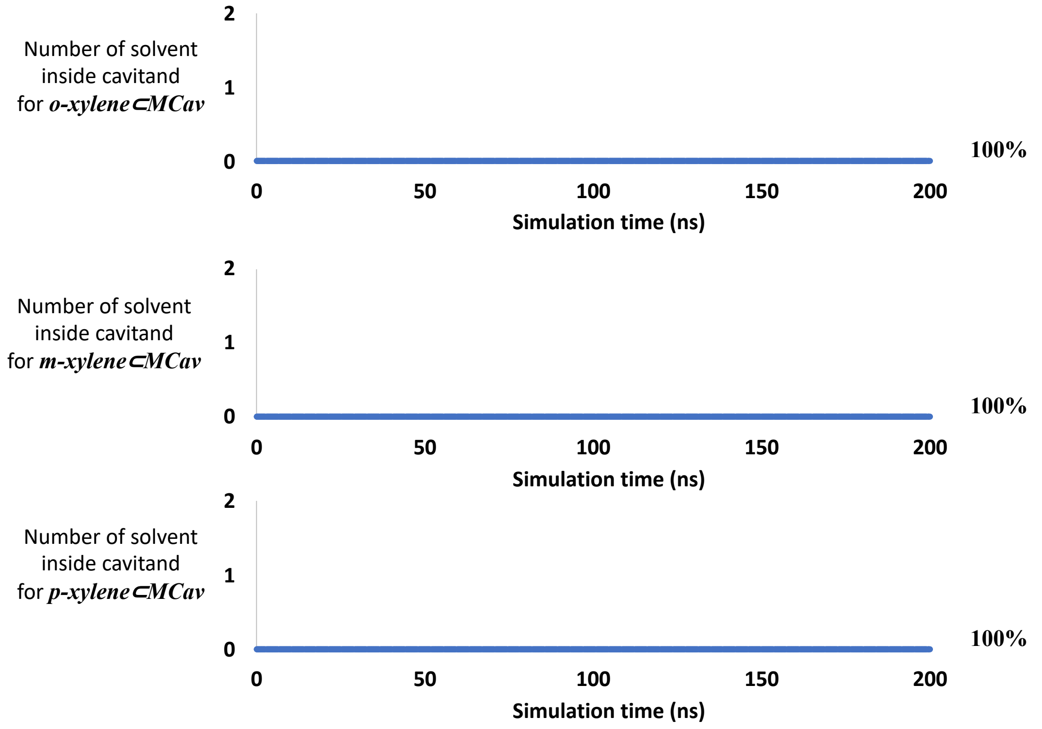

Figure S14. *Number of solvent molecules in* ***MCav*** *during the MD simulations of the cavitand-guest complexes.*

6. MD simulations of cavitand-guest complexes.

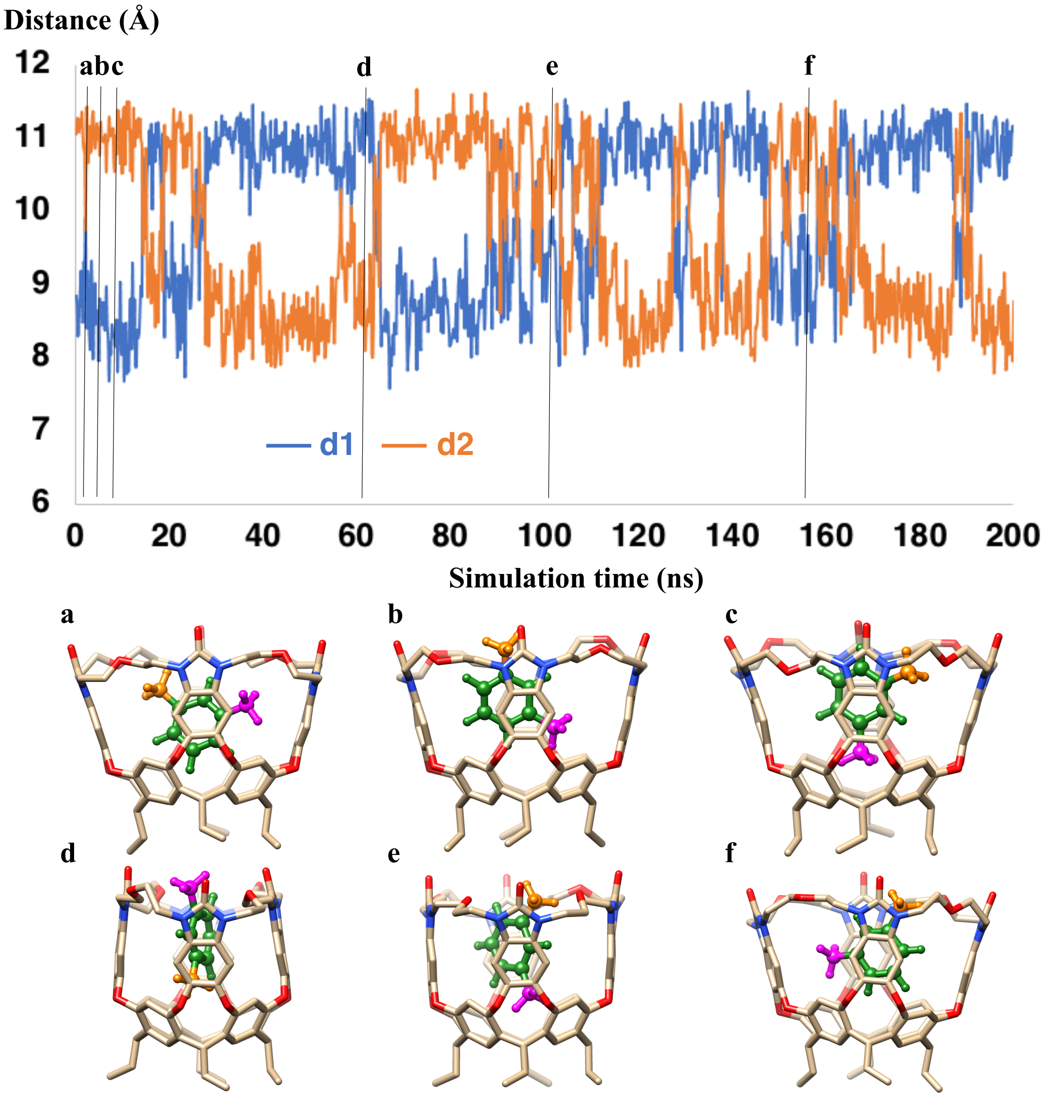

Figure S15. *Molecular dynamics simulations of **m-xylene⊂OCav**. Upper panel shows the flexibility of the **OCav** described by the short and long distances between the cavitand walls, **d1** and **d2**, and bottom panel shows geometries of representative MD snapshots.*

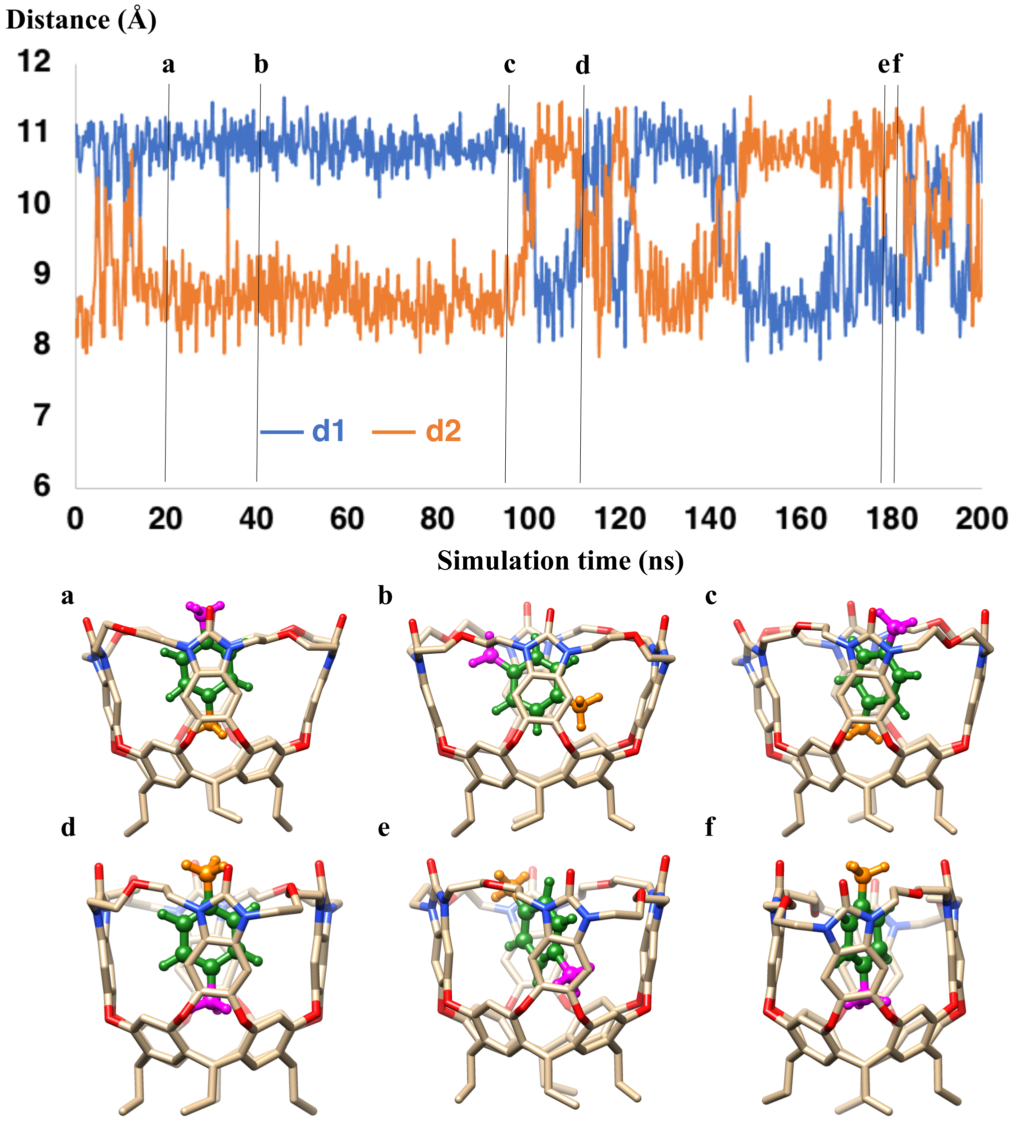

Figure S16. *Molecular dynamics simulations of* ***p-xylene⊂OCav****. Upper panel shows the flexibility of the* ***OCav*** *described by the short and long distances between the cavitand walls,* ***d1*** *and* ***d2****, and bottom panel shows geometries of representative MD snapshots.*

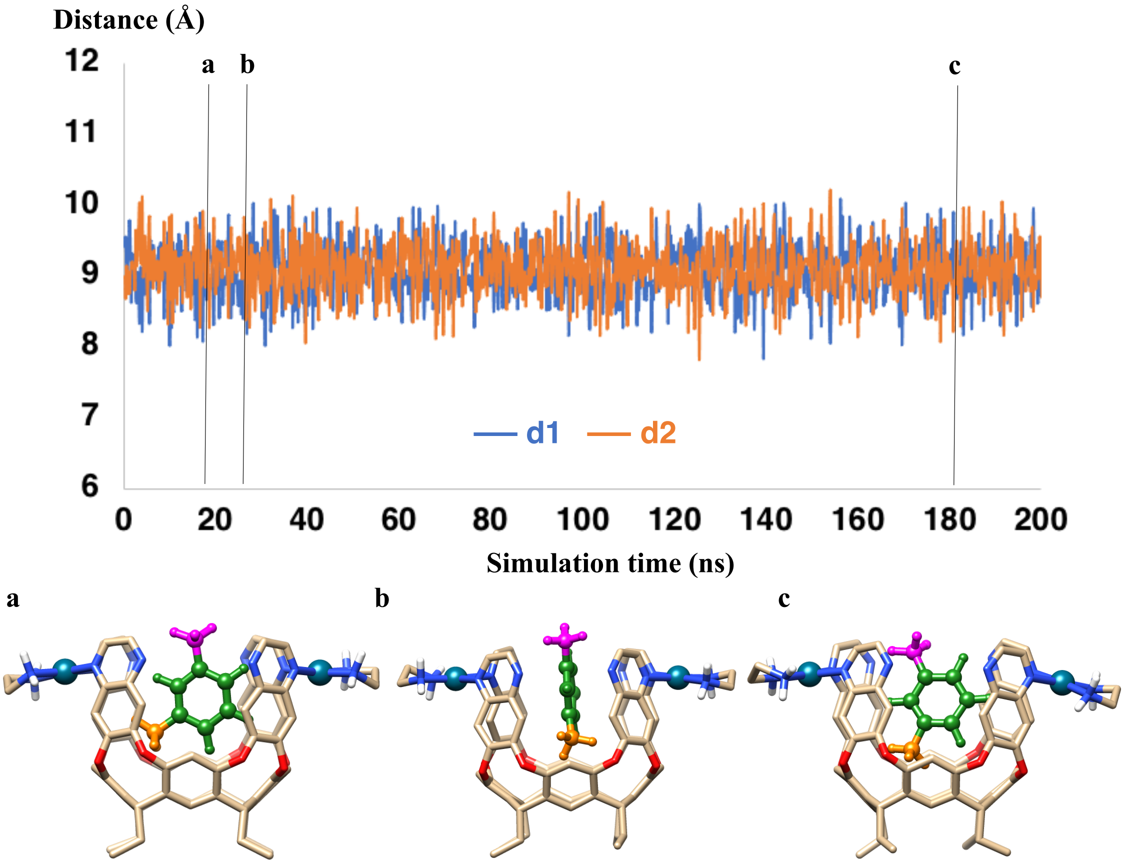

Figure S17. *Molecular dynamics simulations of* ***m-xylene⊂MCav****. Upper panel shows the flexibility of the* ***MCav*** *described by the short and long distances between the cavitand walls,* ***d1*** *and* ***d2****, and bottom panel shows geometries of representative MD snapshots.*

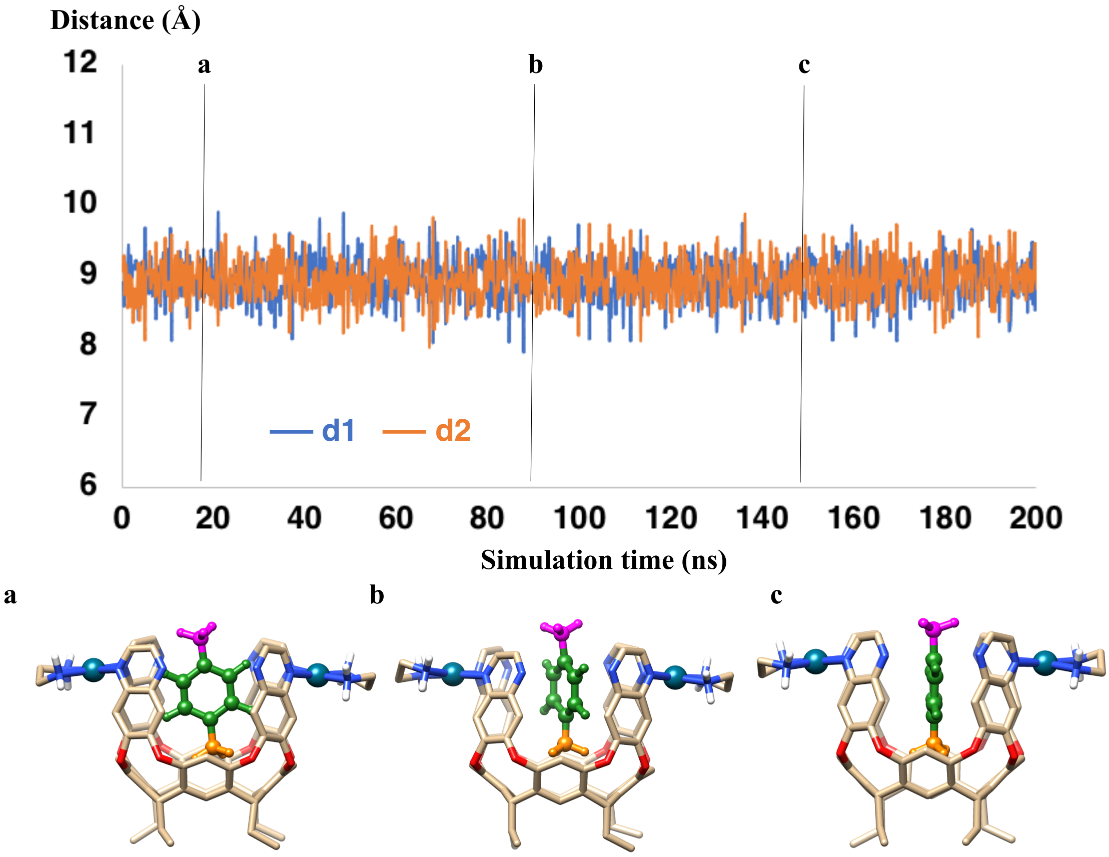

Figure S18. *Molecular dynamics simulations of* ***p-xylene⊂MCav****. Upper panel shows the flexibility of the* ***MCav*** *described by the short and long distances between the cavitand walls,* ***d1*** *and* ***d2****, and bottom panel shows geometries of representative MD snapshots.*

7. Optimized geometries of cavitand-guest complexes.

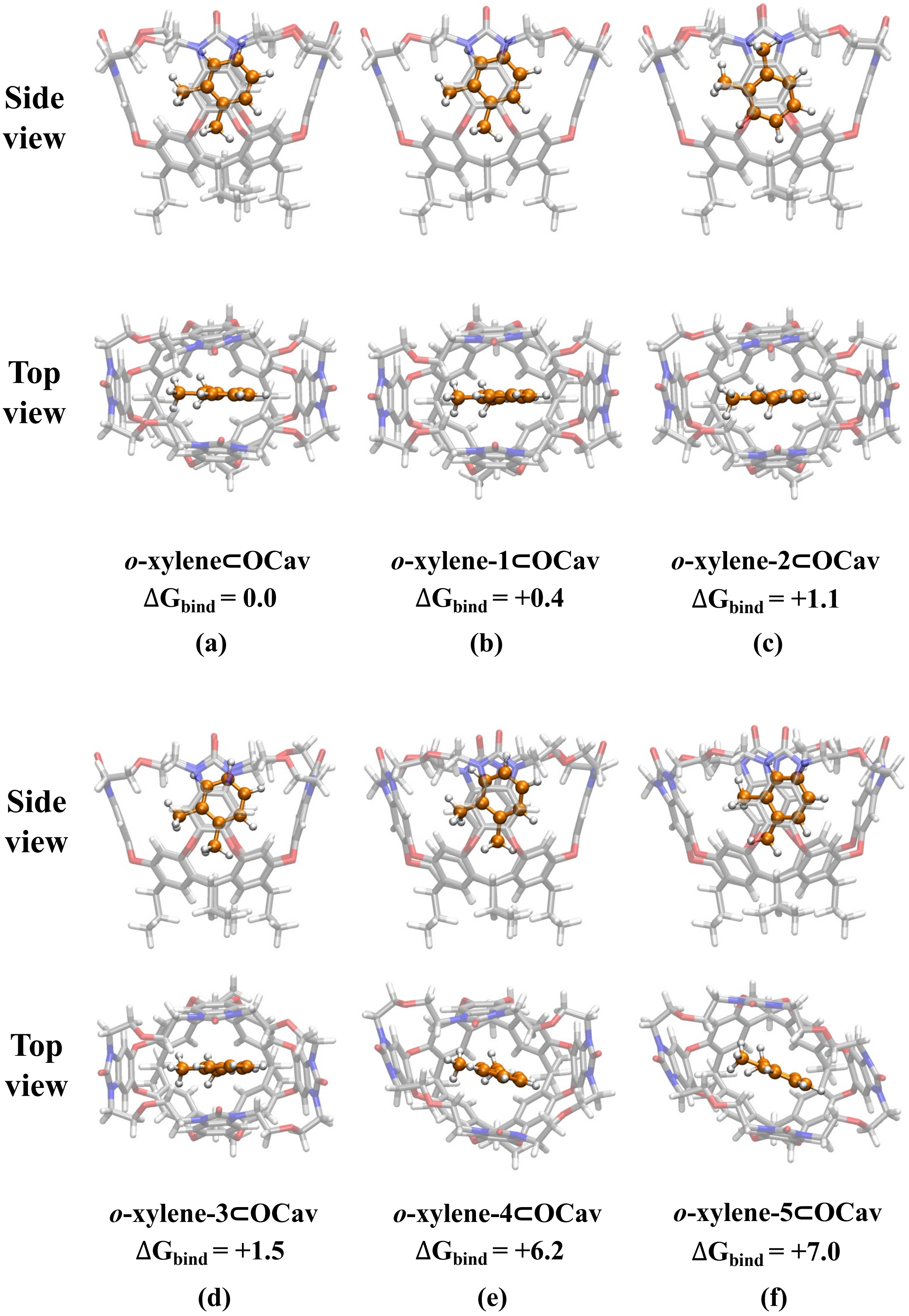

Figure S19. *DFT-optimized geometries of* ***o-xylene⊂OCav*** *complex. Relative energies are given in kcal/mol.*

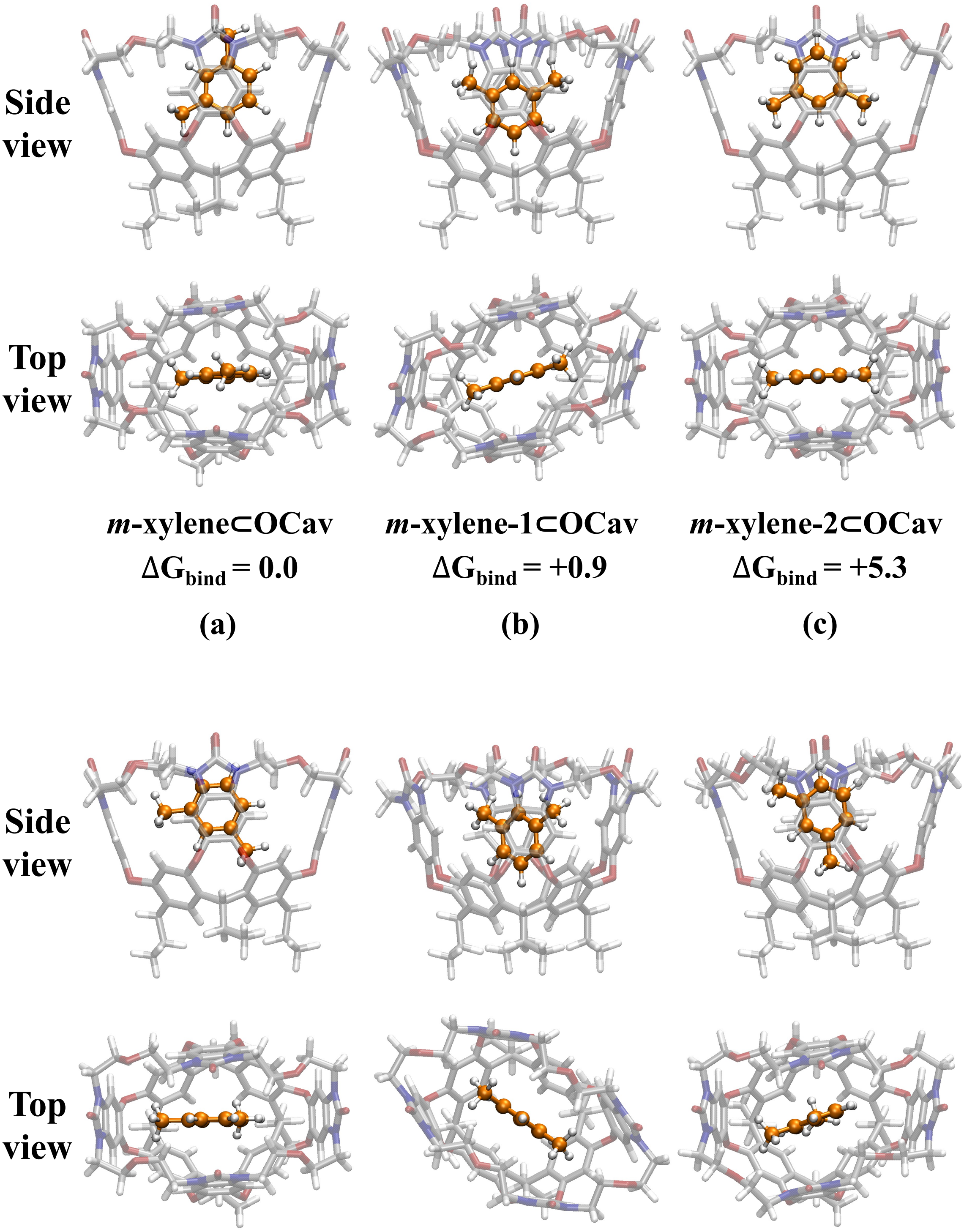

Figure S20. *DFT-optimized geometries of* ***m-xylene⊂OCav*** *complex. Relative energies are given in kcal/mol.*

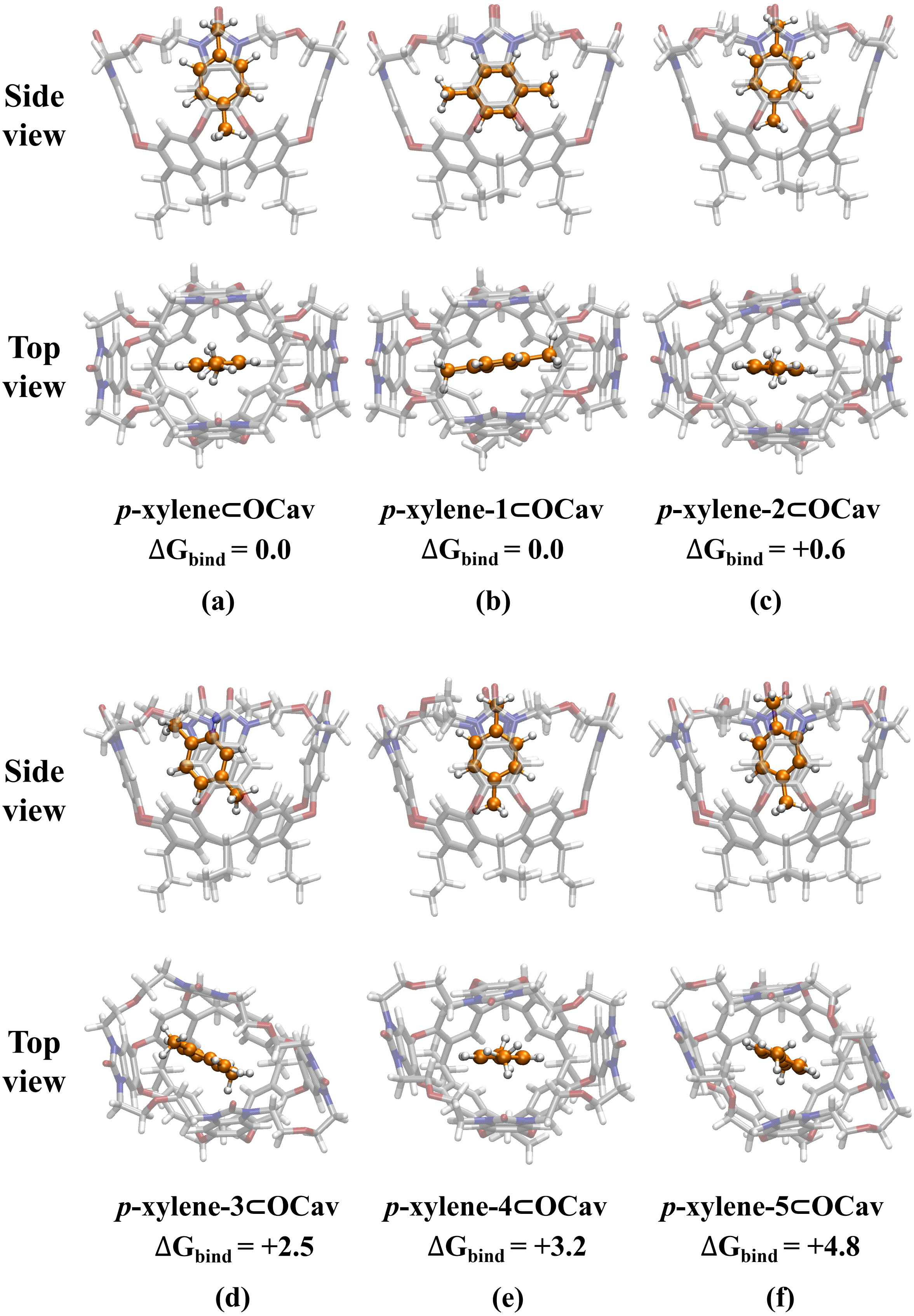

Figure S21. *DFT-optimized geometries of* ***p-xylene⊂OCav*** *complex. Relative energies are given in kcal/mol.*

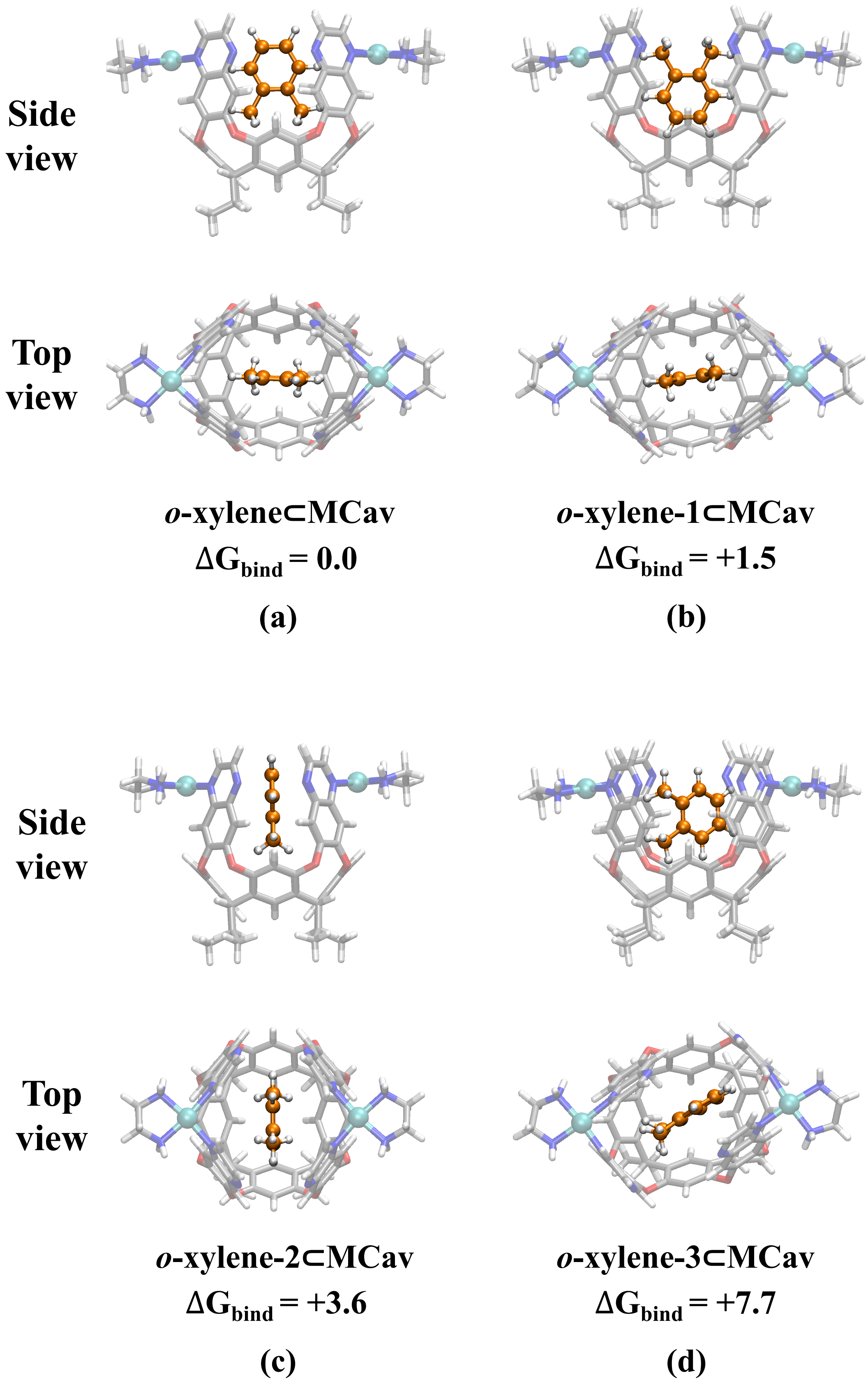

Figure S22. *DFT-optimized geometries of **o-xylene⊂MCav** complex. Relative energies are given in kcal/mol.*

Side view

Top view

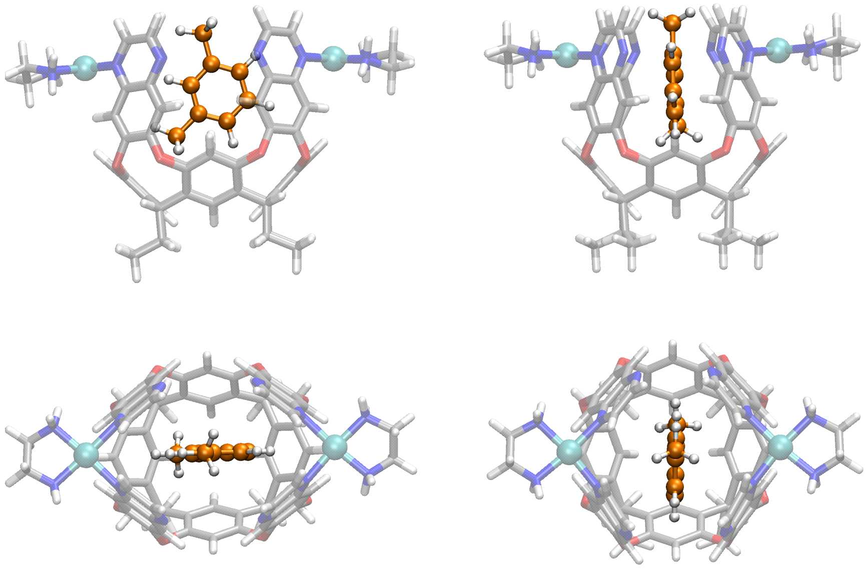

***m*-xylene⊂MCav**
ΔG_{bind} = 0.0
(a)

***m*-xylene-1⊂MCav**
ΔG_{bind} = +1.4
(b)

Side view

Top view

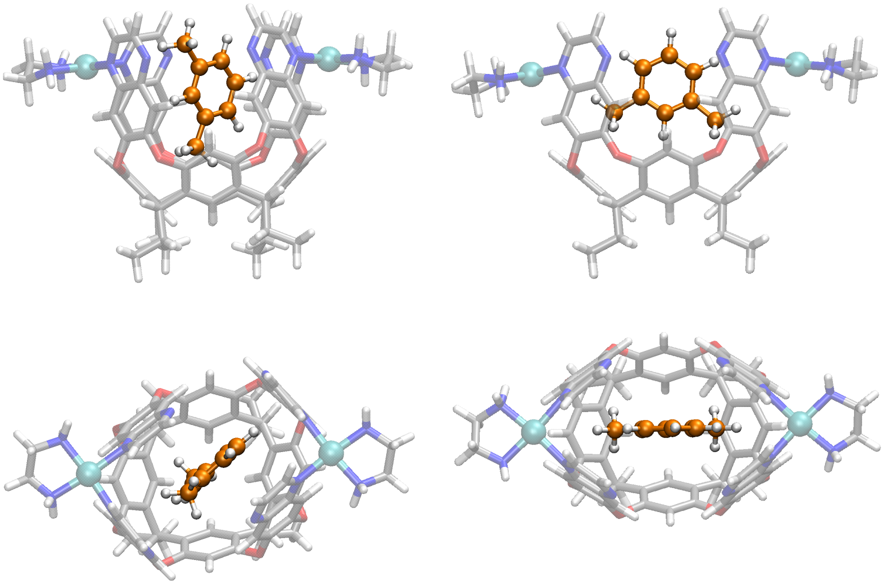

***m*-xylene-2⊂MCav**
ΔG_{bind} = +2.0
(c)

***m*-xylene-3⊂MCav**
ΔG_{bind} = +17.9
(d)

Figure S23. *DFT-optimized geometries of **m-xylene⊂MCav** complex. Relative energies are given in kcal/mol.*

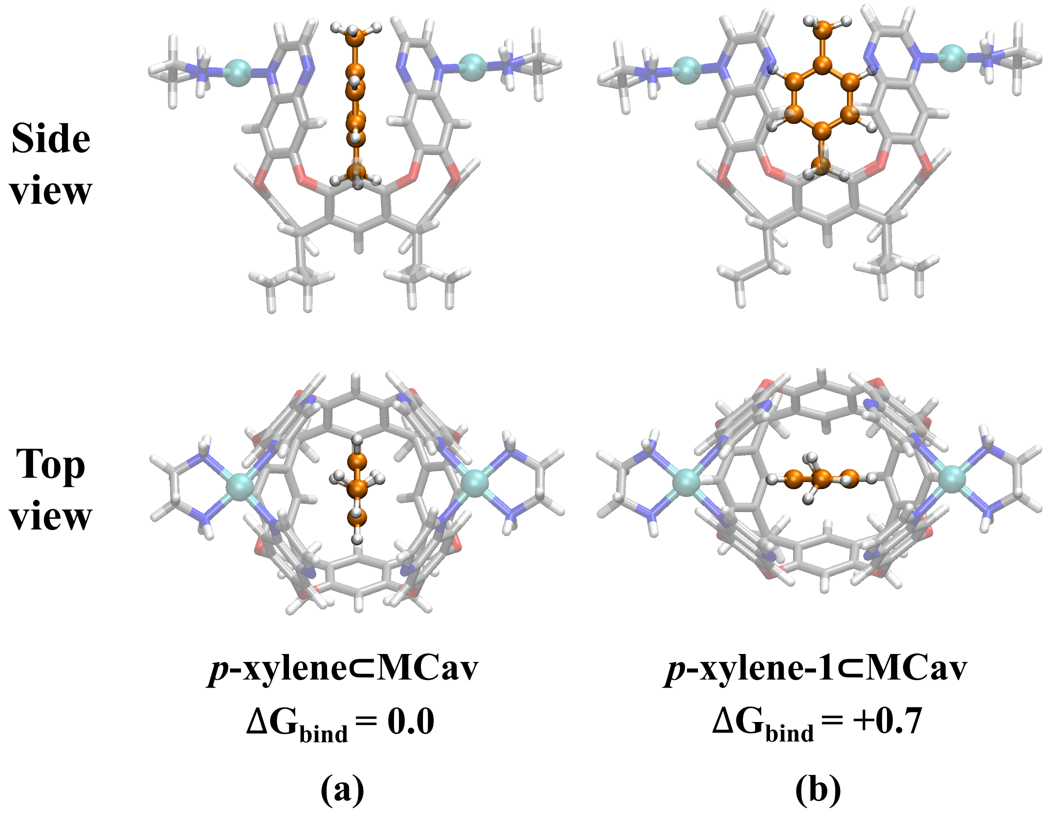

Figure S24. *DFT-optimized geometries of* ***p-xylene⊂MCav*** *complex. Relative energies are given in kcal/mol.*

8. Absolute energies and energy corrections.

The calculated energies of all compounds are given in Table S1. The geometry optimizations were performed with the SMD continuum solvent model (water as solvent).

Table S1. *Calculated energies and energy corrections (in atomic units).*

	B3LYP-D3(BJ)/ 6-311+G(2d,2p)	***Thermal correction to Gibbs free energy (quasi-RRHO, 298.15 K)***	***Total Gibbs free energy***
***p*-xylene⊂OCav**	-5046.499151	1.478863	-5045.020288
***p*-xylene-1⊂OCav**	-5046.501607	1.481284	-5045.020323
***p*-xylene-2⊂OCav**	-5046.498569	1.479316	-5045.019253
***p*-xylene-3⊂OCav**	-5046.496288	1.479926	-5045.016362
***p*-xylene-4⊂OCav**	-5046.495531	1.480282	-5045.015249
***p*-xylene-5⊂OCav**	-5046.493034	1.480407	-5045.012627
***p*-xylene**	-311.008447	0.123679	-310.884768
***o*-xylene⊂OCav**	-5046.508619	1.481744	-5045.026875
***o*-xylene-1⊂OCav**	-5046.507094	1.480911	-5045.026183
***o*-xylene-2⊂OCav**	-5046.506456	1.481310	-5045.025146
***o*-xylene-3⊂OCav**	-5046.504951	1.480421	-5045.024530
***o*-xylene-4⊂OCav**	-5046.495190	1.478271	-5045.016919
***o*-xylene-5⊂OCav**	-5046.493657	1.477864	-5045.015793
***o*-xylene**	-311.009532	0.125343	-310.884189
***m*-xylene⊂OCav**	-5046.502666	1.478395	-5045.024271
***m*-xylene-1⊂OCav**	-5046.504842	1.482009	-5045.022833
***m*-xylene-2⊂OCav**	-5046.496856	1.481067	-5045.015789
***m*-xylene-3⊂OCav**	-5046.497158	1.481457	-5045.015701
***m*-xylene-4⊂OCav**	-5046.491963	1.476728	-5045.015235
***m*-xylene-5⊂OCav**	-5046.491358	1.476990	-5045.014368
***m*-xylene**	-311.008764	0.123535	-310.885229
***p*-xylene⊂MCav**	-4606.752831	1.335101	-4605.417730
***p*-xylene-1⊂MCav**	-4606.753774	1.337115	-4605.416659
***o*-xylene⊂MCav**	-4606.753653	1.338797	-4605.414856
***o*-xylene-1⊂MCav**	-4606.748986	1.336575	-4605.412411
***o*-xylene-2⊂MCav**	-4606.746946	1.337800	-4605.409146
***o*-xylene-3⊂MCav**	-4606.739718	1.337066	-4605.402652
***m*-xylene⊂MCav**	-4606.748633	1.335767	-4605.412866
***m*-xylene-1⊂MCav**	-4606.746610	1.336012	-4605.410598
***m*-xylene-2⊂MCav**	-4606.742688	1.333002	-4605.409686
***m*-xylene-3⊂MCav**	-4606.722523	1.338166	-4605.384357

9. Cartesian coordinates of optimized geometries.

***p*-xylene⊂OCav**

C	1.30277	-0.61896	0.01052
C	1.29766	-2.01462	-0.00598
C	0.10153	-2.73939	-0.01450
C	-1.09786	-2.01183	-0.00307
C	-1.09901	-0.61916	0.01455
C	0.10426	0.10106	0.01657
H	2.24817	-0.08056	0.00881
H	-2.04100	-2.54228	-0.01494
H	2.23789	-2.54890	-0.02170
C	0.10701	-4.24784	-0.03070
H	-0.69750	-4.64447	-0.65480
H	1.05291	-4.63377	-0.41951
H	-0.02681	-4.66835	0.97099
C	1.74950	4.15229	1.76889
C	1.23153	3.43435	2.85194
C	1.94117	2.30273	3.26922
C	3.16610	1.96281	2.70405
C	3.67647	2.74317	1.67238
C	2.96178	3.82988	1.15075
H	1.18635	4.99902	1.39419
H	3.73730	1.13157	3.09663
C	3.45636	4.56739	-0.09250
H	4.54025	4.44989	-0.12477
C	-0.02830	3.87068	3.58396
H	0.01970	3.41388	4.57328
C	2.88627	3.83643	-1.30462
C	3.56822	2.74805	-1.86592
C	1.64295	4.16323	-1.85492
C	2.99878	1.96580	-2.86390
C	1.06202	3.44137	-2.90368
H	1.10328	5.01195	-1.45224
C	1.74203	2.30483	-3.35541
H	3.54574	1.13373	-3.28775
C	-1.28856	3.32619	2.92706
C	-1.92322	3.99538	1.87415
C	-1.87339	2.13758	3.37665
C	-3.13159	3.56615	1.31888
H	-1.45663	4.88908	1.47726
C	-3.09352	1.69130	2.87602
C	-3.72079	2.42047	1.87284
H	-3.56976	0.81568	3.29768
C	-0.23833	3.87433	-3.56271
H	-0.24418	3.41998	-4.55437
C	-3.76171	4.25713	0.11424
C	-1.46037	3.32742	-2.83928
C	-2.02657	3.98754	-1.74364
C	-2.08569	2.15332	-3.27298
C	-3.21268	3.57094	-1.13390
H	-1.52736	4.86923	-1.35893
C	-3.28615	1.72099	-2.71768
C	-3.85114	2.44767	-1.67599
H	-3.79524	0.85557	-3.12151
H	-4.83239	4.05225	0.14816
O	1.47147	1.56025	4.36005
O	-1.28086	1.43559	4.43323
O	4.97945	2.51499	1.22035
O	4.89485	2.52289	-1.48832
O	1.20320	1.55448	-4.40817
O	-1.55211	1.44664	-4.35801
O	-5.13363	2.12006	-1.22665
O	-5.02115	2.08616	1.48612
C	0.84205	0.34810	4.07867
C	-0.56085	0.28523	4.11026
C	1.61524	-0.80127	3.88165
C	-1.23531	-0.92888	3.93830
C	0.94190	-2.00197	3.71551
H	2.69637	-0.74740	3.89505
C	-0.46446	-2.06514	3.74029
H	-2.31532	-0.97416	3.99721
C	-5.26187	0.88770	0.82569
C	-5.58138	-0.26248	1.55250
C	-5.32939	0.90764	-0.57627
C	-5.95642	-1.38697	0.82865
H	-5.56940	-0.24665	2.63484
C	-5.72348	-0.21944	-1.30261
C	-6.03115	-1.36394	-0.57895
H	-5.81253	-0.17302	-2.38071
C	5.27331	1.33149	0.55398
C	5.74063	0.22168	1.26401
C	5.23039	1.33614	-0.84901
C	6.14742	-0.87994	0.52202
H	5.80896	0.25086	2.34414
C	5.65378	0.23256	-1.59438
C	6.10519	-0.87361	-0.88684
H	5.65727	0.26940	-2.67634
C	0.57972	0.35131	-4.07710
C	1.35294	-0.79950	-3.88520
C	-0.82338	0.29573	-4.05394
C	0.67973	-1.99233	-3.66944
H	2.43298	-0.75546	-3.94256
C	-1.49781	-0.91116	-3.83504
C	-0.72689	-2.04806	-3.64292

H	-2.57962	-0.94986	-3.85233	C	-2.42340	-3.93260	-3.53629
N	-1.07796	-3.38604	-3.48518	O	4.70673	-3.78979	-2.58069
N	1.14248	-3.29765	-3.53080	C	-6.43564	-3.14977	2.60092
N	6.63057	-2.08763	-1.31562	C	-5.06580	-3.27285	3.24557
N	1.40582	-3.30800	3.58853	H	-4.58266	-2.29049	3.30054
N	-6.37017	-2.65084	1.23655	C	-2.90986	-3.75929	2.35930
N	6.69481	-2.09976	0.90586	C	-2.15594	-3.95824	3.67531
N	-0.81237	-3.40826	3.62449	C	6.91712	-2.56591	2.26512
N	-6.49636	-2.61106	-0.98216	C	5.62022	-2.82068	3.01418
C	-0.30812	5.40130	-3.76771	C	3.47379	-3.50982	2.24696
H	-0.32527	5.92134	-2.80439	C	2.79485	-3.73233	3.59906
H	0.61716	5.71103	-4.26611	H	-2.69026	-3.47107	4.49564
C	-3.58703	5.78442	0.11479	H	-2.06895	-5.02075	3.90700
H	-3.95644	6.16087	1.07523	H	-2.46094	-4.36980	1.57231
H	-2.52826	6.05901	0.06759	H	-2.85876	-2.70719	2.05486
C	-4.33836	6.45922	-1.03206	H	-5.19242	-3.64209	4.27083
H	-5.40630	6.21643	-0.99910	H	-7.06017	-2.48275	3.20319
H	-4.23867	7.54771	-0.97754	H	-6.92283	-4.12462	2.55221
H	-3.95385	6.13711	-2.00507	O	4.86278	-3.80718	2.31246
C	-0.07633	5.39714	3.79688	O	-4.26685	-4.16998	2.47424
H	0.87721	5.69767	4.24529	H	3.04068	-4.17686	1.49718
H	-0.14090	5.92130	2.83786	H	3.32450	-2.47363	1.92008
C	3.15906	6.07530	-0.08496	H	5.86045	-3.16189	4.02865
H	3.49740	6.48549	-1.04296	H	5.04315	-1.89295	3.10043
H	2.08150	6.26257	-0.03761	H	7.49928	-3.48529	2.18843
C	-1.23091	5.83158	4.69844	H	7.51227	-1.82661	2.80990
H	-1.18123	5.32705	5.66988	H	2.81598	-4.79030	3.86477
H	-1.20023	6.91082	4.87796	H	3.31356	-3.17182	4.38180
H	-2.20036	5.59505	4.24934	H	7.30173	-1.79662	-3.27345
C	3.85320	6.80245	1.06590	H	7.34530	-3.45957	-2.66489
H	3.66533	7.87957	1.01814	H	5.56481	-3.14154	-4.36931
H	3.49670	6.44353	2.03645	H	3.21777	-2.44504	-2.05331
H	4.93734	6.64748	1.03141	H	2.98156	-4.14022	-1.57597
C	-1.51350	5.82994	-4.60334	H	2.51383	-4.79758	-3.89145
H	-1.50319	6.91064	-4.77613	H	2.97359	-3.19704	-4.49250
H	-1.51090	5.33271	-5.57985	H	-2.33740	-4.99691	-3.76038
H	-2.45572	5.58024	-4.10560	H	-2.95404	-3.45020	-4.36245
C	6.95284	-2.87284	-0.21770	C	-3.19457	-3.72931	-2.23137
C	0.06742	-4.16610	-3.40757	H	-2.75742	-4.33390	-1.43342
C	-6.65867	-3.43104	0.12564	H	-3.15770	-2.67587	-1.92947
C	0.33273	-4.18187	3.51167	C	-5.36538	-3.23190	-3.08935
O	-7.02435	-4.61041	0.12569	H	-4.85937	-2.26402	-3.17770
O	0.38675	-5.41078	3.38975	H	-5.57000	-3.59870	-4.10247
O	7.41361	-4.01840	-0.23701	C	-6.68318	-3.07087	-2.34879
O	0.11918	-5.39376	-3.28337	H	-7.20447	-4.02714	-2.28723
C	6.75371	-2.54342	-2.69090	H	-7.32264	-2.36429	-2.88637
C	5.40353	-2.80101	-3.33922	O	-4.54422	-4.15465	-2.37290
H	4.81931	-1.87461	-3.37897	C	0.09808	1.60049	-0.00297
C	3.33223	-3.48622	-2.37761	H	-0.32968	1.97168	-0.93670
C	2.52355	-3.73325	-3.65182	H	-0.50736	2.00355	0.81097

H	1.10752	2.00159	0.08844
H	-2.04401	-0.07905	0.01704

***p*-xylene-1⊂OCav**

C	-2.04367	3.86465	-1.65399
C	-1.51882	3.15266	-2.73343
C	-2.21744	2.01682	-3.15745
C	-3.46300	1.69024	-2.62217
C	-3.98554	2.46983	-1.59028
C	-3.26467	3.54534	-1.05635
H	-1.48904	4.71685	-1.27861
H	-4.02232	0.85374	-3.02224
C	-3.74164	4.27524	0.19452
H	-4.81858	4.12702	0.27032
C	-0.29423	3.64326	-3.48537
H	-0.31695	3.14721	-4.45595
C	-3.09109	3.54531	1.36230
C	-3.71828	2.43128	1.93711
C	-1.82187	3.88788	1.83393
C	-3.06169	1.62713	2.86728
C	-1.16584	3.15929	2.82973
H	-1.33522	4.76862	1.43234
C	-1.77863	1.98662	3.28185
H	-3.55067	0.76223	3.29805
C	1.01482	3.23477	-2.83438
C	1.62557	4.00497	-1.84088
C	1.69900	2.10084	-3.28524
C	2.91313	3.74002	-1.36743
H	1.08545	4.85277	-1.43701
C	3.00209	1.82317	-2.87167
C	3.60775	2.66680	-1.94151
H	3.54424	0.99103	-3.30322
C	0.11352	3.65429	3.48269
H	0.16724	3.15840	4.45206
C	3.51681	4.50901	-0.19923
C	1.36791	3.24947	2.73071
C	1.84838	3.99741	1.65420
C	2.13476	2.15567	3.15015
C	3.08554	3.75276	1.05304
H	1.24047	4.81224	1.27836
C	3.39757	1.90799	2.61329
C	3.87048	2.72157	1.58350
H	4.00817	1.10617	3.00996
H	4.60079	4.42624	-0.27457
O	-1.70953	1.26322	-4.22705
O	1.11079	1.31985	-4.28919
O	-5.29304	2.25545	-1.12857
O	-5.06989	2.22289	1.63224
O	-1.14312	1.24402	4.28611
O	1.67518	1.37011	4.21890
O	5.18828	2.58892	1.12125
O	4.96989	2.54151	-1.63796
C	-0.96979	0.11997	-3.90953
C	0.43760	0.15425	-3.91965
C	-1.65863	-1.08126	-3.69596
C	1.18781	-1.00622	-3.69464
C	-0.90835	-2.22721	-3.47703
H	-2.74133	-1.09631	-3.71894
C	0.49872	-2.18754	-3.46898
H	2.26970	-0.96969	-3.72707
C	5.43360	1.41184	-0.97402
C	5.83216	0.29579	-1.71430
C	5.55151	1.43875	0.42671
C	6.30422	-0.80119	-1.00856
H	5.74984	0.29908	-2.79375
C	6.07510	0.34984	1.12849
C	6.42757	-0.77351	0.39339
H	6.16949	0.39104	2.20631
C	-5.58478	1.08598	-0.43209
C	-6.04124	-0.03356	-1.13271
C	-5.46396	1.06682	0.96865
C	-6.32231	-1.17653	-0.39692
H	-6.13954	0.00133	-2.21047
C	-5.79064	-0.07182	1.70961
C	-6.19466	-1.19627	1.00476
H	-5.70661	-0.06308	2.78883
C	-0.39599	0.12483	3.91537
C	-1.07053	-1.08112	3.69067
C	1.01061	0.18089	3.90424
C	-0.30823	-2.21678	3.46608
H	-2.15247	-1.11285	3.72427
C	1.77432	-0.97509	3.69488
C	1.09843	-2.16626	3.47706
H	2.85563	-0.92170	3.72057
N	1.55159	-3.46977	3.28857
N	-0.66866	-3.54533	3.25433
N	-6.54064	-2.47620	1.42990
N	-1.27612	-3.55707	-3.28421
N	6.73186	-2.05618	-1.43308
N	-6.74733	-2.44459	-0.78551
N	0.94399	-3.48990	-3.25829
N	6.93019	-2.01239	0.78277
C	0.08419	5.17274	3.76077
H	1.04831	5.44457	4.20488
H	0.00861	5.73628	2.82518
C	3.16822	6.00537	-0.18267
H	3.46567	6.42733	-1.14916
H	2.08710	6.15733	-0.10340
C	3.86973	6.75203	0.95104
H	4.95695	6.63176	0.88774

H	3.64638	7.82269	0.91252	H	7.09118	-3.56169	-2.78638
H	3.55014	6.37844	1.92907	O	-4.71280	-4.11937	-1.99316
C	-0.35219	5.16145	-3.75919	O	4.50844	-3.71593	-2.22137
H	-0.35695	5.72359	-2.81982	H	-2.90747	-4.59019	-1.19431
H	0.57126	5.43971	-4.27915	H	-3.11573	-2.86993	-1.57348
C	-3.48382	5.79013	0.17527	H	-5.52079	-3.43427	-3.79590
H	-3.80861	6.19522	1.14025	H	-4.86620	-2.17961	-2.72446
H	-2.41369	6.00722	0.09831	H	-7.33501	-3.89455	-2.12512
C	-1.56091	5.56248	-4.60363	H	-7.37119	-2.24490	-2.76627
H	-2.50107	5.34535	-4.08715	H	-2.57653	-5.14339	-3.54353
H	-1.54179	6.63393	-4.82620	H	-3.18611	-3.56913	-4.06863
H	-1.57134	5.02073	-5.55613	H	-6.84924	-2.38213	3.49469
C	-4.22564	6.49155	-0.96176	H	-6.80121	-4.00246	2.78276
H	-3.88019	6.13850	-1.93886	H	-4.79060	-3.48268	4.16405
H	-5.30372	6.30513	-0.90259	H	-2.68651	-2.64722	1.83766
H	-4.06813	7.57385	-0.92307	H	-2.48595	-4.28985	1.19800
C	-1.04929	5.58045	4.70085	H	-1.93819	-5.16575	3.40838
H	-1.00113	6.65035	4.92671	H	-2.50331	-3.68906	4.20266
H	-2.02951	5.37621	4.25957	H	2.95216	-4.96585	3.56358
H	-0.98899	5.03336	5.64837	H	3.45584	-3.35038	4.07812
C	-6.80576	-3.27624	0.32569	C	3.60654	-3.67414	1.93897
C	0.47244	-4.32533	3.12666	H	3.24940	-4.40567	1.20936
C	7.04638	-2.83778	-0.32849	H	3.35010	-2.67423	1.58143
C	-0.14374	-4.34119	-3.12425	C	5.66095	-2.78422	2.75606
O	7.39682	-4.02083	-0.33664	H	5.04894	-1.87402	2.74073
O	-0.11138	-5.56053	-2.92916	H	5.79683	-3.08265	3.80317
O	-7.07627	-4.48011	0.33430	C	7.02508	-2.50466	2.14822
O	0.51961	-5.54462	2.93487	H	7.62453	-3.41580	2.12028
C	-6.33674	-3.01558	2.76558	H	7.55153	-1.76660	2.75907
C	-4.86572	-3.11254	3.13389	O	5.02135	-3.81965	2.00705
H	-4.39624	-2.12161	3.09651	C	1.45778	-0.60840	0.10048
C	-2.82734	-3.71022	2.05911	C	0.71271	0.57768	0.04378
C	-2.01887	-4.08299	3.30505	C	-0.67576	0.54111	-0.04753
C	2.93640	-3.90851	3.29522	C	-1.35728	-0.68290	-0.10029
O	-4.21370	-3.99190	2.21705	C	-0.61164	-1.86556	-0.05346
C	6.56435	-2.60661	-2.76930	C	0.77589	-1.82903	0.05679
C	5.10304	-2.79770	-3.13917	H	1.22809	1.53350	0.07460
H	4.57054	-1.83899	-3.10389	H	-1.12069	-2.82222	-0.10207
C	3.10713	-3.52257	-2.06361	C	-2.84764	-0.70374	-0.23747
C	2.32551	-3.93853	-3.31222	H	-3.28021	0.16788	0.24559
C	-6.79958	-2.94438	-2.15072	H	-3.14603	-0.66760	-1.28886
C	-5.41481	-3.12932	-2.74724	H	-3.29581	-1.59635	0.20068
C	-3.30977	-3.88558	-1.92685	C	2.94687	-0.54892	0.23955
C	-2.62917	-4.08530	-3.28301	H	3.24015	-0.47524	1.29046
H	2.78367	-3.50734	-4.20644	H	3.44124	-1.42582	-0.17951
H	2.31614	-5.02341	-3.42368	H	3.33530	0.33347	-0.26134
H	2.80206	-4.12659	-1.20556	H	1.33461	-2.75748	0.10899
H	2.89891	-2.47190	-1.83720	H	-1.24020	1.46871	-0.08008
H	5.05295	-3.17362	-4.16874				
H	7.03632	-1.94132	-3.49737				

***p*-xylene-2⊂OCav**

C	-2.02180	3.97294	1.65464
C	-3.18226	3.56663	0.99190
C	-3.85041	2.44459	1.49753
C	-3.34631	1.71740	2.57013
C	-2.17469	2.14617	3.18737
C	-1.51545	3.30994	2.77716
H	-1.49881	4.85112	1.29424
H	-3.88237	0.85758	2.95082
C	-0.31914	3.84616	3.54916
H	-0.36851	3.39599	4.54146
C	-3.67763	4.26584	-0.26966
H	-4.74553	4.06079	-0.35132
C	0.99371	3.38681	2.93556
C	1.61883	2.21945	3.38903
C	1.63955	4.11261	1.93009
C	2.87517	1.84278	2.92561
C	2.89405	3.76213	1.42110
H	1.14938	4.99407	1.53501
C	3.50994	2.62778	1.96803
H	3.37519	0.98133	3.34857
C	-2.99876	3.59468	-1.45763
C	-1.76616	4.03189	-1.94674
C	-3.57192	2.47234	-2.07265
C	-1.09482	3.40511	-3.00285
H	-1.31068	4.90569	-1.49646
C	-2.91261	1.78451	-3.08494
C	-1.67438	2.24379	-3.52905
H	-3.37493	0.92592	-3.55534
C	3.53960	4.53653	0.27183
H	4.61713	4.38865	0.35342
C	0.18723	3.99107	-3.58015
C	3.09364	3.87919	-1.03106
C	1.90944	4.23943	-1.68717
C	3.83015	2.81849	-1.57426
C	1.42666	3.55704	-2.81032
H	1.33336	5.07156	-1.29952
C	3.36597	2.09162	-2.66266
C	2.15240	2.44158	-3.23953
H	3.95389	1.28388	-3.07652
H	0.28891	3.56809	-4.58050
O	-5.10761	2.12544	0.97671
O	-4.88456	2.12423	-1.74104
O	-1.71563	1.45939	4.31802
O	1.03736	1.49708	4.43698
O	4.84038	2.35219	1.63459
O	5.09626	2.53177	-1.05481
O	1.68393	1.69893	-4.32799
O	-1.05089	1.55994	-4.58506
C	-5.27176	0.92297	0.30022
C	-5.14799	0.91965	-1.09869
C	-5.69155	-0.21299	0.99710
C	-5.44291	-0.22218	-1.84944
C	-5.97218	-1.34877	0.25009
H	-5.82204	-0.17806	2.07118
C	-5.85088	-1.35367	-1.15428
H	-5.38944	-0.19275	-2.93022
C	-0.34546	0.41300	-4.20955
C	-1.02024	-0.80737	-4.08208
C	1.04287	0.49912	-4.02033
C	-0.26911	-1.91444	-3.71263
H	-2.08408	-0.87132	-4.27323
C	1.78970	-0.61243	-3.61750
C	1.10829	-1.80623	-3.44561
H	2.85987	-0.54421	-3.47485
C	-1.01182	0.27525	4.10137
C	-1.71515	-0.92608	3.96161
C	0.39233	0.29636	4.14808
C	-0.97183	-2.09311	3.87116
H	-2.79791	-0.93343	3.96030
C	1.13777	-0.88443	4.05217
C	0.43543	-2.07294	3.91344
H	2.21781	-0.86241	4.12644
C	5.13998	1.19202	0.92949
C	5.42769	0.00502	1.61038
C	5.28505	1.28786	-0.46243
C	5.84362	-1.08021	0.84876
H	5.36378	-0.03549	2.68999
C	5.72016	0.20105	-1.22576
C	5.98706	-0.98144	-0.54962
H	5.87210	0.30223	-2.29279
N	6.47587	-2.20382	-0.99975
N	6.25139	-2.36196	1.20844
N	0.86108	-3.39816	3.87726
N	-6.44562	-2.60173	0.62318
N	-0.60200	-3.25907	-3.56427
N	-1.35835	-3.42943	3.81304
N	-6.26301	-2.60813	-1.59207
N	1.55691	-3.07128	-3.08542
C	3.28172	6.05153	0.31393
H	3.74203	6.48965	-0.57867
H	2.21134	6.26985	0.24087
C	0.11947	5.52261	-3.74796
H	0.00952	6.01525	-2.77653
H	1.08459	5.85175	-4.14892
C	-1.00541	5.96764	-4.68128
H	-0.98233	7.05185	-4.82917
H	-0.91098	5.49186	-5.66388
H	-1.98827	5.70636	-4.27758
C	-3.50621	5.79369	-0.24226

H	-2.45159	6.06932	-0.14073
H	-3.83082	6.18248	-1.21389
C	-0.37704	5.37421	3.74519
H	0.53412	5.67559	4.27399
H	-0.35587	5.89087	2.78029
C	-4.31240	6.45141	0.87696
H	-5.37711	6.20777	0.78999
H	-3.97367	6.11612	1.86248
H	-4.21222	7.54068	0.84249
C	-1.60606	5.81940	4.53652
H	-1.59064	6.90072	4.70487
H	-2.53268	5.57707	4.00671
H	-1.64326	5.32662	5.51456
C	3.85340	6.71077	1.56839
H	4.92950	6.52318	1.65449
H	3.70146	7.79433	1.54417
H	3.37521	6.32627	2.47456
C	-0.23561	-4.24156	3.77986
C	6.64588	-3.06409	0.07624
C	0.51082	-3.97611	-3.14950
C	-6.59063	-3.40152	-0.50129
O	0.56480	-5.18698	-2.90652
O	-6.97102	-4.57599	-0.52846
O	-0.21609	-5.47477	3.70038
O	7.08650	-4.21643	0.03698
C	2.23682	-3.86417	3.92106
C	2.92158	-3.69986	2.56329
H	2.46584	-4.37259	1.83336
C	5.09299	-3.05525	3.29569
C	6.40959	-2.87670	2.56115
C	6.94592	-2.48678	-2.34897
O	4.30275	-4.03181	2.61864
C	-1.92001	-3.84172	-3.73445
C	-2.78945	-3.65041	-2.49097
H	-2.39478	-4.24578	-1.66545
C	-4.88327	-3.22285	-3.54630
C	-6.28013	-3.09298	-2.96257
C	-2.72089	-3.92747	3.74174
C	-3.28595	-3.82010	2.32363
C	-5.52524	-3.19628	2.84615
C	-6.75816	-3.04910	1.97145
H	-6.87458	-2.41569	-3.58336
H	-6.77637	-4.06420	-2.94146
H	-4.40163	-2.24011	-3.60144
H	-4.96075	-3.61612	-4.56729
H	-2.78766	-2.59545	-2.18912
H	-2.38752	-3.37241	-4.60389
H	-1.79213	-4.90428	-3.94774
O	-4.66079	-4.17989	2.27658
O	-4.11893	-4.10020	-2.71935

H	-5.00195	-2.23707	2.92941
H	-5.84309	-3.49410	3.85301
H	-3.15005	-2.79908	1.94803
H	-2.75582	-4.50973	1.66244
H	-2.71401	-4.96726	4.07192
H	-3.32924	-3.35005	4.44294
H	-7.26972	-4.00748	1.87113
H	-7.44767	-2.33798	2.43712
H	2.76512	-3.29439	4.69044
H	2.22228	-4.91305	4.22051
H	2.79550	-2.66936	2.21199
H	5.30926	-3.37260	4.32350
H	4.55214	-2.10342	3.34680
H	6.92476	-3.83425	2.47067
H	7.04829	-2.19509	3.13250
H	7.54359	-3.39844	-2.29548
H	7.59799	-1.66773	-2.66782
C	5.83013	-2.65063	-3.36245
H	5.12706	-1.81247	-3.28728
H	6.26258	-2.63360	-4.37235
C	3.83259	-3.89492	-3.65062
H	3.75902	-3.27615	-4.55326
H	3.58374	-4.92448	-3.92191
C	2.87696	-3.38568	-2.57119
H	3.27740	-2.47607	-2.11880
H	2.77201	-4.13281	-1.78232
O	5.16347	-3.89269	-3.13797
C	-1.15446	-1.91978	0.10607
C	-1.07254	-0.52952	0.03625
C	0.17063	0.11427	-0.00829
C	1.32517	-0.67802	0.01423
C	1.23860	-2.06846	0.07450
C	-0.00176	-2.71725	0.13463
C	0.25937	1.60656	-0.13452
H	-0.01992	1.92189	-1.14255
H	-0.41871	2.10886	0.55881
H	1.27297	1.95932	0.05623
H	-1.98416	0.06415	0.00872
H	2.14834	-2.65736	0.06607
H	-2.12833	-2.39472	0.13060
C	-0.08742	-4.21880	0.24230
H	0.57472	-4.71092	-0.47418
H	0.20164	-4.56974	1.23739
H	-1.10468	-4.57246	0.05821
H	2.30125	-0.19898	-0.02924

***p*-xylene-3⊂OCav**

C	1.74012	3.57941	2.66511
C	0.66790	2.95820	3.31063
C	0.82277	1.61701	3.68313

C	2.02508	0.94121	3.48415	H	0.33508	-1.53403	4.02022
C	3.08778	1.61502	2.88432	C	-3.06287	-1.80788	3.63591
C	2.96709	2.94478	2.45399	H	-4.47526	-0.16143	3.61504
H	1.62979	4.61067	2.35478	C	-4.77642	1.84760	-1.01372
H	2.15310	-0.06539	3.86159	C	-5.58681	0.80707	-0.54891
C	4.13116	3.64596	1.75756	C	-4.12663	1.76580	-2.25683
H	5.04898	3.23976	2.18444	C	-5.71547	-0.31492	-1.35583
C	-0.61011	3.69826	3.66785	H	-6.10156	0.90020	0.39922
H	-0.98606	3.24170	4.58423	C	-4.26199	0.63697	-3.06926
C	4.09882	3.23573	0.29207	C	-5.05520	-0.39994	-2.59919
C	4.89159	2.18217	-0.17253	H	-3.80152	0.61327	-4.04809
C	3.20415	3.81732	-0.61613	C	4.74309	-0.01053	2.05263
C	4.73864	1.66800	-1.45538	C	4.43970	-1.33506	2.38426
C	3.03420	3.34204	-1.91982	C	5.61989	0.28276	0.99276
H	2.58923	4.64616	-0.28285	C	5.06378	-2.33338	1.64573
C	3.77273	2.21303	-2.28995	H	3.77761	-1.56331	3.21005
H	5.35059	0.84076	-1.78890	C	6.27708	-0.72314	0.28076
C	-1.66918	3.47121	2.60199	C	5.99899	-2.03562	0.63758
C	-1.63530	4.17711	1.39664	H	6.97228	-0.46932	-0.50968
C	-2.70960	2.55603	2.78383	C	2.67773	0.50375	-3.46699
C	-2.61111	4.03956	0.40758	C	3.14533	-0.69844	-2.92446
H	-0.81165	4.86016	1.22313	C	1.36917	0.61130	-3.96381
C	-3.70961	2.39413	1.82859	C	2.27782	-1.77735	-2.92202
C	-3.65231	3.13441	0.65185	H	4.14769	-0.77626	-2.52609
H	-4.52382	1.70213	2.00401	C	0.52305	-0.50439	-4.02533
C	2.10572	4.00537	-2.92416	C	1.00174	-1.69884	-3.50958
H	2.41045	3.64158	-3.90621	H	-0.46963	-0.41945	-4.44896
C	-2.51228	4.79985	-0.90582	N	0.44437	-2.97211	-3.43052
C	0.65135	3.58724	-2.75539	N	2.42246	-3.06841	-2.42737
C	-0.20879	4.27220	-1.89043	N	6.50252	-3.24850	0.17430
C	0.09902	2.55613	-3.52646	N	-1.66180	-3.57114	3.64235
C	-1.58251	4.02807	-1.83340	N	-6.47280	-1.47490	-1.21939
H	0.20387	5.06096	-1.27299	N	5.01060	-3.72167	1.75156
C	-1.26595	2.27582	-3.49519	N	-3.80568	-2.98421	3.55977
C	-2.09450	3.02491	-2.66534	N	-5.44296	-1.59915	-3.18829
H	-1.68105	1.52267	-4.15210	C	2.29055	5.53741	-2.93827
H	-3.50210	4.78004	-1.36231	H	1.98922	5.97310	-1.97999
O	-0.17787	1.04241	4.46771	H	3.36329	5.73653	-3.03860
O	-2.78652	1.82088	3.97659	C	-2.12745	6.27829	-0.71876
O	4.36036	1.04132	2.87394	H	-2.81889	6.70862	0.01423
O	5.86107	1.61735	0.66926	H	-1.12694	6.37186	-0.28433
O	3.52964	1.61110	-3.53272	C	-2.19552	7.07230	-2.02266
O	0.90996	1.84655	-4.42915	H	-1.96886	8.12882	-1.84872
O	-3.48281	2.89269	-2.75345	H	-1.47979	6.69451	-2.75941
O	-4.69463	3.02811	-0.27798	H	-3.19510	7.01082	-2.46724
C	-1.08235	0.09651	4.00160	C	-0.38708	5.19263	3.96788
C	-2.43014	0.47558	3.86206	H	0.42088	5.27066	4.70428
C	-0.70024	-1.24418	3.89149	H	-0.04150	5.72292	3.07478
C	-3.44097	-0.47427	3.68660	C	4.17091	5.17052	1.95724
C	-1.70612	-2.18035	3.69600	H	5.00730	5.55480	1.36291

H	3.27014	5.64242	1.55113
C	-1.64685	5.87059	4.50441
H	-1.45036	6.91746	4.75582
H	-2.45447	5.84996	3.76583
H	-2.00903	5.36875	5.40860
C	4.35166	5.57417	3.42020
H	4.43297	6.66128	3.51763
H	3.50831	5.24580	4.03519
H	5.26159	5.12982	3.83875
C	1.53357	6.21674	-4.07848
H	0.45292	6.07199	-3.98690
H	1.72675	7.29403	-4.08651
H	1.84260	5.81212	-5.04885
C	5.85086	-4.29807	0.80768
C	1.29726	-3.81768	-2.73672
C	-6.31300	-2.27067	-2.34457
C	-2.94977	-4.07637	3.56485
O	-6.87401	-3.35051	-2.56074
O	-3.28136	-5.26660	3.54002
O	6.00705	-5.50450	0.59942
O	1.09832	-5.00516	-2.45822
C	7.42876	-3.42266	-0.93408
C	6.74160	-3.31448	-2.28438
H	6.19001	-2.36963	-2.34744
C	4.58346	-4.08637	-2.97330
C	3.64125	-3.61498	-1.85977
C	-0.82281	-3.39621	-3.99930
O	5.86217	-4.42884	-2.45629
C	-7.50588	-1.76279	-0.23118
C	-7.18654	-2.92666	0.68543
H	-8.12481	-3.26516	1.14819
C	-6.03325	-3.61179	2.59416
C	-5.23609	-3.14658	3.79562
C	4.01650	-4.46262	2.50585
C	2.64867	-4.37717	1.83249
C	0.38110	-4.28107	2.48269
C	-0.46521	-4.38635	3.74525
H	-5.63055	-2.19702	4.16144
H	-5.35685	-3.88843	4.59023
H	-6.99283	-4.00549	2.95873
H	-5.51039	-4.42726	2.07743
H	-6.77665	-3.76512	0.10780
H	-7.68171	-0.85964	0.35501
H	-8.42772	-1.99737	-0.77111
O	1.67659	-4.77745	2.78919
O	-6.27328	-2.52685	1.70288
H	-0.07667	-4.84150	1.65606
H	0.44997	-3.23139	2.17645
H	2.46185	-3.34147	1.51860
H	2.60887	-5.01138	0.93604

H	4.36074	-5.49350	2.59366
H	3.94734	-4.03421	3.50901
H	-0.78005	-5.41231	3.93857
H	0.12488	-4.03714	4.59829
H	8.21242	-2.66467	-0.85505
H	7.89078	-4.40520	-0.82311
H	7.50103	-3.31787	-3.07684
H	4.17267	-4.98607	-3.44006
H	4.66754	-3.31091	-3.74483
H	4.10914	-2.83402	-1.25589
H	3.37885	-4.44479	-1.20213
H	-0.74711	-4.45909	-4.23681
H	-0.96615	-2.84772	-4.93343
C	-1.98493	-3.14560	-3.04098
H	-1.91384	-3.82554	-2.19092
H	-1.94623	-2.11598	-2.66586
C	-3.59431	-2.40830	-4.62843
H	-2.99640	-1.50326	-4.47545
H	-3.39977	-2.76852	-5.64625
C	-5.07435	-2.09296	-4.50546
H	-5.66982	-2.99137	-4.67467
H	-5.34763	-1.35131	-5.26306
O	-3.23971	-3.39814	-3.66316
C	-2.29031	-0.47560	0.22712
C	-1.22843	0.42578	0.17650
C	0.08980	-0.02213	0.02146
C	0.30616	-1.40165	-0.08050
C	-0.76017	-2.30182	-0.04720
C	-2.07875	-1.85556	0.11096
C	1.22389	0.95972	-0.07650
H	1.03738	1.84434	0.53657
H	2.17342	0.51585	0.23325
H	1.34245	1.30254	-1.10763
H	-1.42209	1.49021	0.26221
H	-0.56251	-3.36501	-0.14539
H	-3.29927	-0.10420	0.36407
H	1.31988	-1.77571	-0.19141
C	-3.24358	-2.81274	0.12586
H	-4.04331	-2.45262	0.77458
H	-3.67375	-2.93084	-0.87637
H	-2.94133	-3.80656	0.46997

***p*-xylene-4⊂OCav**

C	1.52368	3.90328	2.46222
C	0.74811	3.12325	3.32538
C	1.27150	1.88775	3.72057
C	2.53167	1.46463	3.30887
C	3.28518	2.28435	2.47455
C	2.79672	3.52264	2.02689
H	1.12305	4.85114	2.12512

H	2.94787	0.53985	3.68571
C	3.60769	4.39020	1.06803
H	4.66090	4.18494	1.26418
C	-0.60342	3.58537	3.84689
H	-0.76362	3.06551	4.79227
C	3.31545	3.90629	-0.34838
C	4.12792	2.93556	-0.94175
C	2.18685	4.32742	-1.06523
C	3.77778	2.32867	-2.14157
C	1.82113	3.77054	-2.29551
H	1.55948	5.10203	-0.63858
C	2.60419	2.71408	-2.77320
H	4.41190	1.57172	-2.58284
C	-1.72855	3.14400	2.92016
C	-2.09735	3.90221	1.80421
C	-2.45731	1.97559	3.16967
C	-3.19049	3.58270	0.99543
H	-1.51834	4.78715	1.56695
C	-3.56130	1.62573	2.39741
C	-3.93118	2.44428	1.33616
H	-4.14880	0.75249	2.65091
C	0.66153	4.28798	-3.13527
H	0.85498	3.95144	-4.15487
C	-3.53645	4.39326	-0.24886
C	-0.68100	3.68701	-2.74291
C	-1.45523	4.24738	-1.72088
C	-1.21825	2.58597	-3.42154
C	-2.74214	3.80490	-1.40776
H	-1.03788	5.07288	-1.15697
C	-2.50740	2.12852	-3.15854
C	-3.26321	2.75176	-2.17249
H	-2.92932	1.32283	-3.74564
H	-4.59194	4.21954	-0.46064
O	0.57200	1.13420	4.66815
O	-2.14976	1.19664	4.29147
O	4.61568	1.94760	2.21167
O	5.33443	2.57918	-0.32622
O	2.22133	2.04988	-3.94393
O	-0.48646	1.95961	-4.44371
O	-4.60936	2.41060	-2.01411
O	-5.12777	2.19777	0.65713
C	-0.07420	-0.02778	4.25905
C	-1.46611	0.00118	4.07438
C	0.64283	-1.22486	4.18463
C	-2.18563	-1.16829	3.80730
C	-0.07396	-2.38250	3.92455
H	1.70735	-1.23673	4.37877
C	-1.46964	-2.35538	3.73934
H	-3.26282	-1.13890	3.70033
C	-5.22916	1.05778	-0.13091
C	-5.73091	-0.12326	0.42234
C	-4.95384	1.16412	-1.50375
C	-5.93683	-1.19149	-0.43944
H	-5.97644	-0.17123	1.47548
C	-5.17539	0.09092	-2.37223
C	-5.66689	-1.08448	-1.81896
H	-5.00373	0.20335	-3.43535
C	4.96552	0.90494	1.36021
C	5.03692	-0.41678	1.81901
C	5.42120	1.24951	0.07705
C	5.58716	-1.36045	0.95761
H	4.70994	-0.67607	2.81819
C	6.00438	0.30852	-0.77263
C	6.08679	-0.99585	-0.30861
H	6.38213	0.60343	-1.74354
C	1.53799	0.84915	-3.73907
C	2.22985	-0.28415	-3.29771
C	0.17094	0.79039	-4.04844
C	1.51857	-1.46978	-3.21444
H	3.28350	-0.23642	-3.05690
C	-0.53121	-0.42147	-4.00705
C	0.16712	-1.54872	-3.60026
H	-1.57542	-0.46336	-4.28942
N	-0.19927	-2.88962	-3.51212
N	1.91397	-2.74920	-2.84768
N	6.64335	-2.13554	-0.87891
N	0.32048	-3.71575	3.86542
N	-6.45386	-2.46416	-0.22199
N	5.85270	-2.72085	1.11388
N	-1.88330	-3.67705	3.58940
N	-6.04810	-2.28871	-2.40128
C	0.63237	5.83039	-3.17186
H	0.43166	6.23746	-2.17534
H	1.63746	6.17120	-3.44410
C	-3.34662	5.90923	-0.07571
H	-2.30522	6.14932	0.16093
H	-3.55778	6.38397	-1.04042
C	-4.25912	6.49257	1.00234
H	-4.03294	6.07102	1.98700
H	-4.14046	7.57849	1.06894
H	-5.31166	6.28113	0.78331
C	-0.64315	5.09529	4.15248
H	0.21791	5.32812	4.78883
H	-0.51461	5.68259	3.23768
C	3.39428	5.90027	1.26170
H	2.35809	6.18124	1.04782
H	3.56228	6.13169	2.31940
C	-1.93377	5.51855	4.85266
H	-2.80891	5.34150	4.21950
H	-2.07759	4.95875	5.78360

H	-1.91131	6.58427	5.10078
C	4.33336	6.73242	0.38958
H	4.15350	6.54804	-0.67436
H	4.19225	7.80217	0.57236
H	5.38153	6.49005	0.59715
C	-0.38657	6.38165	-4.16804
H	-1.40870	6.10863	-3.88879
H	-0.33331	7.47388	-4.21439
H	-0.20049	5.99249	-5.17535
C	6.44569	-3.21999	-0.04053
C	0.87222	-3.63950	-3.04565
C	-6.49626	-3.16288	-1.42053
C	-0.78399	-4.52111	3.65098
O	-6.88792	-4.32206	-1.58587
O	-0.78818	-5.75373	3.54524
O	6.76009	-4.39418	-0.25593
O	0.90115	-4.86154	-2.86463
C	7.21885	-2.23289	-2.21149
C	6.15779	-2.33310	-3.29063
H	5.44596	-1.50534	-3.18740
C	4.12799	-3.54119	-3.56664
C	3.23824	-3.12623	-2.38994
C	-1.47009	-3.45888	-3.92416
O	5.49280	-3.59165	-3.16753
C	-6.96178	-2.99594	1.03375
C	-5.87473	-3.23698	2.06685
H	-5.34706	-2.30313	2.29150
C	-3.59761	-3.89990	1.83340
C	-3.23474	-4.12802	3.30331
C	5.38344	-3.59447	2.17540
C	3.92102	-3.95816	1.96236
C	2.17315	-5.16359	2.99194
C	1.66540	-4.22516	4.09763
H	-3.92207	-3.58369	3.95655
H	-3.29133	-5.18827	3.55367
H	-2.99489	-4.55000	1.19532
H	-3.39080	-2.85814	1.56278
H	-6.34697	-3.58575	2.99369
H	-7.70103	-2.30146	1.44561
H	-7.46780	-3.93213	0.79280
O	3.58759	-5.01892	2.85099
O	-4.95873	-4.21219	1.56866
H	1.98544	-6.20722	3.24799
H	1.67093	-4.94734	2.04369
H	3.29287	-3.07807	2.14554
H	3.76501	-4.27573	0.92091
H	5.99988	-4.49460	2.15165
H	5.52845	-3.10022	3.13863
H	1.70574	-4.72258	5.07095
H	2.32434	-3.35796	4.14432
H	7.84169	-1.35190	-2.38676
H	7.86141	-3.11508	-2.22674
H	6.63137	-2.25165	-4.27818
H	3.84814	-4.54119	-3.90916
H	3.99062	-2.84434	-4.40257
H	3.66970	-2.26963	-1.86605
H	3.14034	-3.94394	-1.67329
H	-1.30666	-4.51750	-4.13318
H	-1.77369	-2.96908	-4.85408
C	-2.56034	-3.29418	-2.86452
H	-2.33699	-3.91198	-1.99330
H	-2.62148	-2.24866	-2.53853
C	-4.46044	-2.79840	-4.22422
H	-3.95585	-1.82762	-4.17071
H	-4.42538	-3.13811	-5.26632
C	-5.91456	-2.66338	-3.79948
H	-6.43491	-3.61514	-3.91494
H	-6.41355	-1.91917	-4.42767
O	-3.81560	-3.73795	-3.36481
C	1.14311	-2.15241	0.28261
C	1.28209	-0.76313	0.29033
C	0.16891	0.07077	0.14074
C	-1.09119	-0.52958	0.00328
C	-1.22630	-1.91619	0.00136
C	-0.11065	-2.75653	0.14023
C	0.31903	1.56287	0.06417
H	-0.36927	2.07246	0.74309
H	1.33593	1.87200	0.30742
H	0.09381	1.91598	-0.94537
H	2.27037	-0.32162	0.39270
H	-2.20950	-2.35229	-0.12983
H	2.02461	-2.77712	0.36606
H	-1.97141	0.09820	-0.12101
C	-0.24822	-4.25856	0.13469
H	-0.61958	-4.64025	1.08975
H	-0.94744	-4.59636	-0.63430
H	0.71410	-4.73734	-0.05980

***p*-xylene-5⊂OCav**

C	0.68972	4.10457	2.41338
C	-0.30213	3.44190	3.14381
C	0.06214	2.25388	3.79056
C	1.35622	1.74486	3.69582
C	2.31573	2.44134	2.96525
C	2.00805	3.65331	2.32634
H	0.42567	5.02553	1.90894
H	1.63464	0.84876	4.23573
C	3.07599	4.43909	1.57019
H	4.02392	4.23831	2.07127
C	-1.71645	3.99188	3.23590

H	-2.14742	3.58537	4.15179	C	-4.06680	-0.62550	-2.77626
C	3.18781	3.86926	0.16492	C	-4.77409	-1.64133	-2.13902
C	4.15818	2.90800	-0.13003	H	-3.38744	-0.82744	-3.59512
C	2.29526	4.21887	-0.85645	C	4.20078	0.97580	2.33227
C	4.18130	2.24967	-1.35343	C	4.05929	-0.34628	2.77188
C	2.31139	3.60985	-2.11626	C	5.07878	1.28912	1.27992
H	1.55021	4.98151	-0.65733	C	4.84577	-1.31160	2.14893
C	3.22802	2.57120	-2.31028	H	3.39174	-0.59089	3.58916
H	4.93425	1.50178	-1.56031	C	5.90092	0.32810	0.68997
C	-2.59010	3.48192	2.09303	C	5.78342	-0.97264	1.15433
C	-2.62329	4.08746	0.83138	H	6.59450	0.60647	-0.09345
C	-3.42074	2.37322	2.28745	C	2.60406	0.62030	-3.50191
C	-3.48382	3.66484	-0.19076	C	3.25693	-0.48301	-2.94331
H	-1.93937	4.90464	0.62980	C	1.35714	0.47895	-4.13021
C	-4.34597	1.97561	1.33155	C	2.64115	-1.71808	-3.07256
C	-4.37715	2.63186	0.10772	H	4.21357	-0.37579	-2.44966
H	-5.04012	1.17385	1.54195	C	0.78473	-0.78209	-4.33216
C	1.40057	4.05178	-3.25555	C	1.45084	-1.87937	-3.80483
H	1.84933	3.66591	-4.17173	H	-0.14505	-0.87334	-4.87815
C	-3.41225	4.22783	-1.60311	N	1.16503	-3.24588	-3.82516
C	0.02717	3.41174	-3.14619	N	3.01754	-2.97765	-2.62291
C	-1.00806	4.02520	-2.43481	N	6.47311	-2.13343	0.82028
C	-0.25695	2.19462	-3.77797	N	-0.91961	-3.26126	3.84106
C	-2.29994	3.50089	-2.35652	N	-6.33329	-2.56785	-0.79891
H	-0.80333	4.96676	-1.94111	N	4.98492	-2.68020	2.37513
C	-1.52113	1.61487	-3.69441	N	-2.75413	-3.13693	2.59075
C	-2.52876	2.26701	-2.98674	N	-4.80780	-3.01958	-2.34832
H	-1.73871	0.69744	-4.22672	C	1.34018	5.58582	-3.39333
H	-4.34863	3.96622	-2.09793	H	0.62955	5.82191	-4.19338
O	-0.86010	1.61635	4.63649	H	0.94005	6.04162	-2.48200
O	-3.36504	1.67763	3.49951	C	-3.29712	5.76200	-1.63105
O	3.65211	2.04186	3.03823	H	-4.11849	6.15935	-1.02439
O	5.13932	2.60393	0.82365	H	-2.37465	6.09465	-1.14380
O	3.22513	1.87021	-3.52066	C	-3.37411	6.34109	-3.04313
O	0.70753	1.61300	-4.61714	H	-3.35430	7.43510	-3.01721
O	-3.83683	1.78412	-3.06148	H	-2.53575	6.00650	-3.66153
O	-5.33782	2.25423	-0.84094	H	-4.29966	6.03295	-3.54214
C	-1.41279	0.43249	4.14736	C	-1.75585	5.52768	3.36140
C	-2.65128	0.47843	3.48899	H	-1.06715	5.81516	4.16379
C	-0.74721	-0.77969	4.36148	H	-1.37956	6.00097	2.44880
C	-3.20218	-0.67032	2.91200	C	2.87001	5.96394	1.58262
C	-1.31302	-1.92254	3.81542	H	3.66733	6.40318	0.97277
H	0.17540	-0.80234	4.92652	H	1.93030	6.23629	1.09107
C	-2.49331	-1.85325	3.05358	C	-3.15723	6.05435	3.66727
H	-4.15210	-0.63889	2.39561	H	-3.55377	5.60632	4.58525
C	-5.19264	0.94329	-1.28894	H	-3.14504	7.14053	3.80111
C	-5.94144	-0.06984	-0.68865	H	-3.85514	5.82536	2.85599
C	-4.30377	0.68570	-2.34661	C	2.91094	6.56090	2.98882
C	-5.73179	-1.36247	-1.14485	H	2.82538	7.65141	2.95170
H	-6.64916	0.16274	0.09710	H	2.09390	6.18282	3.61058

H	3.85307	6.31422	3.49097
C	2.70304	6.19876	-3.71232
H	3.12237	5.76953	-4.62915
H	2.62025	7.28076	-3.85466
H	3.41911	6.02204	-2.90369
C	5.95003	-3.20600	1.52557
C	2.13037	-3.93073	-3.09350
C	-5.72938	-3.60644	-1.49009
C	-1.80430	-4.01695	3.07988
O	-5.97880	-4.81150	-1.38903
O	-1.76089	-5.23618	2.88256
O	6.29211	-4.38939	1.44137
O	2.19567	-5.14999	-2.90789
C	7.58656	-2.22947	-0.11203
C	7.14705	-2.32720	-1.56118
H	6.43472	-1.52581	-1.78795
C	5.40770	-3.57686	-2.62100
C	4.16488	-3.26859	-1.78325
C	0.26982	-3.91169	-4.76435
O	6.56353	-3.61187	-1.79249
C	-7.41571	-2.74090	0.15791
C	-6.92986	-2.82001	1.59291
H	-6.27651	-1.96747	1.81146
C	-5.07589	-3.94176	2.59489
C	-3.87017	-3.52391	1.74830
C	4.13228	-3.49728	3.21959
C	2.77972	-3.71981	2.54079
C	1.45902	-3.60716	4.50973
C	-0.00466	-3.85094	4.81176
H	-3.55294	-4.34371	1.10054
H	-4.12596	-2.67185	1.11360
H	-5.23103	-3.22052	3.40695
H	-4.89122	-4.92217	3.04259
H	-7.79257	-2.76820	2.27002
H	-8.10640	-1.90071	0.04890
H	-7.94721	-3.65646	-0.10924
O	1.84800	-4.38828	3.37888
O	-6.23883	-4.05645	1.78537
H	1.63401	-2.54257	4.31473
H	2.04824	-3.88900	5.39151
H	2.36748	-2.75522	2.22373
H	2.91466	-4.34428	1.65484
H	4.64384	-4.44459	3.39510
H	4.01269	-2.98815	4.17893
H	-0.20720	-4.92340	4.83089
H	-0.23823	-3.43889	5.79878
H	8.21946	-1.34779	0.02004
H	8.16827	-3.11149	0.16330
H	8.02192	-2.19656	-2.21147
H	5.30841	-4.56238	-3.08419

H	5.51737	-2.83356	-3.42044
H	4.34458	-2.39804	-1.14772
H	3.92172	-4.11339	-1.13569
H	0.53538	-4.97039	-4.75788
H	0.46188	-3.51544	-5.76690
C	-1.20266	-3.74706	-4.44957
H	-1.44746	-2.68913	-4.30074
H	-1.78049	-4.10632	-5.31048
C	-2.51713	-3.86036	-2.47704
H	-2.60117	-4.44429	-1.55679
H	-2.18513	-2.84909	-2.21520
C	-3.88161	-3.78397	-3.16488
H	-4.30496	-4.77901	-3.30732
H	-3.80340	-3.29947	-4.14105
O	-1.53263	-4.49696	-3.27959
C	1.22142	-0.43087	0.00896
C	1.38816	-1.81723	-0.00216
C	0.28663	-2.68097	-0.00163
C	-0.99248	-2.10591	0.01677
C	-1.16321	-0.72430	0.00938
C	-0.05647	0.13670	0.00684
H	2.09454	0.21693	0.00734
H	2.38947	-2.23092	-0.01394
H	-1.86272	-2.75090	0.02718
H	-2.16581	-0.30344	0.00839
C	-0.25143	1.62340	0.04154
H	-1.12327	1.91861	-0.54351
H	-0.42247	1.96113	1.06590
H	0.62155	2.15394	-0.34205
C	0.45222	-4.18001	-0.03310
H	-0.10637	-4.61787	-0.86670
H	1.50008	-4.46115	-0.15544
H	0.08206	-4.64397	0.88798

***p*-xylene**

C	1.421149	0.000138	-0.014040
C	0.697230	-1.199391	-0.008193
C	-0.697462	-1.199270	0.008157
C	-1.421149	0.000403	0.014019
C	-0.697378	1.199678	0.008143
C	0.697610	1.199559	-0.008185
H	1.233695	-2.144724	-0.013979
H	-1.234071	-2.144532	0.013912
H	-1.233792	2.145021	0.013883
H	1.234164	2.144831	-0.013944
C	-2.928882	-0.000185	-0.004474
H	-3.311704	-0.025845	-1.032548
H	-3.333806	-0.875128	0.513002
H	-3.333885	0.898654	0.469894
C	2.928882	-0.000341	0.004540

H	3.334069	0.890559	-0.484528
H	3.311628	-0.008679	1.032930
H	3.333696	-0.883700	-0.498414

***o*-xylene**

C	-1.960773	0.696933	-0.000007
C	-0.745957	1.385533	-0.000059
C	0.477356	0.706059	-0.000080
C	0.477372	-0.706037	-0.000047
C	-0.745928	-1.385538	0.000024
C	-1.960760	-0.696967	0.000035
H	-2.896864	1.247276	0.000043
H	-0.742891	2.472379	-0.000116
H	-0.742837	-2.472385	0.000030
H	-2.896842	-1.247327	0.000163
C	1.775197	-1.471336	-0.000028
H	2.384096	-1.228431	-0.878943
H	2.384428	-1.227717	0.878466
H	1.596221	-2.549439	0.000470
C	1.775166	1.471356	0.000061
H	2.383938	1.228251	0.879034
H	2.384516	1.227916	-0.878381
H	1.596195	2.549463	-0.000150

***m*-xylene**

C	-1.208370	1.133435	0.004937
C	-1.228029	-0.264728	0.001729
C	-0.003833	-0.950611	-0.004259
C	1.220762	-0.277028	-0.006422
C	1.212232	1.126785	-0.005203
C	0.006903	1.824272	0.001048
H	-0.008143	-2.038457	-0.008073
H	2.154670	1.667974	-0.009960
H	0.010011	2.910528	0.001117
C	2.524713	-1.034584	0.004631
H	3.032637	-0.931265	0.970941
H	2.367711	-2.101132	-0.176774
H	3.211636	-0.654423	-0.758783
H	-2.145859	1.682541	0.008459
C	-2.528048	-1.029021	-0.000190
H	-2.621985	-1.648306	-0.899564
H	-2.592000	-1.705101	0.859730
H	-3.386662	-0.353481	0.035278

***o*-xylene⊂OCav**

C	1.762758	4.004208	-1.634704
C	2.962080	3.670792	-0.998926
C	3.695398	2.602504	-1.530711
C	3.221719	1.857828	-2.605592
C	2.007928	2.207985	-3.189174
C	1.278696	3.318298	-2.752587
H	1.190762	4.843300	-1.255614
H	3.804922	1.037798	-3.004071
C	0.047339	3.785881	-3.514240
H	0.110629	3.329879	-4.503003
C	3.430292	4.392984	0.260752
H	4.509501	4.251584	0.328130
C	-1.242103	3.276426	-2.893063
C	-1.833215	2.089972	-3.344996
C	-1.902002	3.978394	-1.879802
C	-3.088206	1.691153	-2.892349
C	-3.140206	3.588199	-1.359726
H	-1.431923	4.869273	-1.481569
C	-3.736807	2.450000	-1.920337
H	-3.567993	0.819511	-3.319362
C	2.806960	3.678595	1.453711
C	1.554526	4.034494	1.959708
C	3.452776	2.587150	2.050736
C	0.930272	3.349844	3.008759
H	1.044011	4.887275	1.528367
C	2.845518	1.845784	3.057198
C	1.582068	2.217367	3.510725
H	3.361663	1.008738	3.509308
C	-3.786245	4.330423	-0.189189
H	-4.860476	4.153557	-0.253982
C	-0.372090	3.852731	3.617245
C	-3.300453	3.672825	1.100296
C	-2.124226	4.066813	1.750769
C	-3.997763	2.584865	1.641501
C	-1.603149	3.383148	2.856518
H	-1.582346	4.925215	1.371257
C	-3.494025	1.856127	2.712305
C	-2.277577	2.229631	3.270574
H	-4.044171	1.018780	3.119381
H	-0.437273	3.398854	4.607108
O	4.975611	2.347815	-1.027898
O	4.777616	2.318995	1.695258
O	1.555716	1.486950	-4.302717
O	-1.215109	1.359985	-4.368088
O	-5.056970	2.139664	-1.574445
O	-5.263310	2.275716	1.134282
O	-1.748130	1.460923	4.313396
O	1.000665	1.468967	4.548440
C	5.210182	1.152182	-0.357183
C	5.096697	1.133484	1.042709
C	5.686356	0.043110	-1.062485
C	5.449174	0.002024	1.784570
C	6.034936	-1.079309	-0.323329
H	5.808183	0.087947	-2.137368
C	5.915194	-1.100769	1.081095

H	5.396708	0.016736	2.865828	H	4.991098	6.434958	-0.822865
C	0.356272	0.304167	4.119555	H	3.575608	6.265216	-1.869551
C	1.102500	-0.863576	3.913419	H	3.749400	7.697531	-0.847285
C	-1.034348	0.322387	3.930424	C	1.230727	5.806754	-4.535603
C	0.423844	-1.981524	3.451312	H	1.163736	6.884612	-4.713300
H	2.168488	-0.876562	4.102172	H	2.174064	5.612031	-4.015828
C	-1.709872	-0.800486	3.439382	H	1.277997	5.307011	-5.509663
C	-0.955446	-1.931909	3.172175	C	-4.181024	6.509388	-1.455682
H	-2.780801	-0.783661	3.286917	H	-5.252776	6.292930	-1.526808
C	0.916860	0.277360	-4.025971	H	-4.058702	7.596414	-1.421359
C	1.694013	-0.870726	-3.836912	H	-3.707178	6.148584	-2.373813
C	-0.487287	0.217131	-4.029859	C	0.429180	-4.241000	-3.350628
C	1.028218	-2.070633	-3.647933	C	-6.613610	-3.364453	-0.037819
H	2.774519	-0.812104	-3.865335	C	-0.206251	-4.006888	2.634493
C	-1.155146	-0.998676	-3.832296	C	6.787052	-3.092016	0.416838
C	-0.379057	-2.133167	-3.635638	O	-0.167975	-5.171027	2.221243
H	-2.236801	-1.041952	-3.860389	O	7.253557	-4.235183	0.438086
C	-5.325874	0.967620	-0.874605	O	0.489915	-5.463612	-3.184138
C	-5.601526	-0.217104	-1.564064	O	-6.948336	-4.552048	0.001445
C	-5.438801	1.039254	0.522519	C	-2.059713	-4.033449	-3.493737
C	-5.961823	-1.324774	-0.806537	C	-2.797912	-3.816420	-2.172080
H	-5.552865	-0.246476	-2.645109	H	-2.359950	-4.439916	-1.388420
C	-5.821314	-0.070295	1.281293	C	-4.928923	-3.297028	-3.090881
C	-6.067142	-1.251880	0.596560	C	-6.327662	-3.170297	-2.516225
H	-5.942054	0.010321	2.354005	C	-6.768380	-2.875873	2.417910
N	-6.486391	-2.501402	1.040661	O	-4.168657	-4.184388	-2.270205
N	-6.327386	-2.616787	-1.172211	C	2.156433	-3.842952	3.448207
N	-0.720340	-3.473233	-3.466628	C	3.153084	-3.658488	2.303379
N	6.586310	-2.298089	-0.703655	H	2.873954	-4.285273	1.453370
N	0.847388	-3.277576	3.165799	C	5.140379	-3.099222	3.502408
N	1.498274	-3.371198	-3.497095	C	6.497917	-2.802845	2.883763
N	6.400976	-2.330915	1.511735	C	2.881939	-3.810093	-3.573530
N	-1.314795	-3.177237	2.670049	C	3.602659	-3.723214	-2.228049
C	-3.571036	5.852598	-0.218129	C	5.758225	-2.985419	-2.937849
H	-4.028674	6.267789	0.686670	C	6.959059	-2.704730	-2.049481
H	-2.506025	6.099904	-0.159759	H	7.020312	-2.054836	3.488399
C	-0.375574	5.381335	3.825892	H	7.106639	-3.707802	2.856838
H	-0.300849	5.902865	2.866112	H	4.547867	-2.181139	3.578606
H	-1.349661	5.653868	4.247070	H	5.293860	-3.483752	4.517647
C	0.739856	5.854622	4.756645	H	3.162041	-2.614638	1.975920
H	0.667071	6.932092	4.934611	H	2.534627	-3.360491	4.353228
H	0.682182	5.348471	5.726827	H	2.023515	-4.905567	3.661742
H	1.728206	5.651995	4.333076	O	4.982446	-4.042051	-2.369706
C	3.167220	5.907948	0.241705	O	4.456543	-4.067877	2.705834
H	2.096258	6.119412	0.159601	H	5.149568	-2.081272	-3.048121
H	3.484702	6.312908	1.209113	H	6.117068	-3.272009	-3.933570
C	0.033158	5.313784	-3.724610	H	3.484795	-2.721086	-1.805934
H	-0.897155	5.569387	-4.244118	H	3.180420	-4.448700	-1.528052
H	0.001490	5.836819	-2.763393	H	2.885646	-4.839623	-3.936194
C	3.912490	6.616134	-0.888837	H	3.388696	-3.185910	-4.314358

H	7.569797	-3.602822	-1.945039
H	7.573535	-1.921331	-2.504325
H	-2.603698	-3.559649	-4.315065
H	-1.969309	-5.098185	-3.713926
H	-2.712138	-2.766702	-1.871938
H	-5.003315	-3.675603	-4.117916
H	-4.444997	-2.313632	-3.129109
H	-6.805118	-4.149220	-2.452881
H	-6.931513	-2.534059	-3.170275
H	-7.363663	-3.790181	2.386988
H	-7.372248	-2.088293	2.877491
C	-5.516238	-3.091052	3.245170
H	-4.864574	-2.211385	3.172149
H	-5.798477	-3.212262	4.300117
C	-3.474097	-4.285567	3.177255
H	-3.349339	-3.833238	4.169035
H	-3.168951	-5.333328	3.239861
C	-2.619467	-3.540327	2.147726
H	-3.123883	-2.619851	1.851787
H	-2.480626	-4.148032	1.250874
O	-4.843206	-4.260527	2.779503
C	2.051377	-1.367369	-0.055933
C	1.544212	-0.071384	0.032048
C	0.166603	0.166279	0.010167
C	-0.724464	-0.921337	-0.099415
C	-0.204138	-2.216614	-0.183592
C	1.170790	-2.445097	-0.163376
C	-0.371006	1.559692	0.132660
H	-0.928012	1.674198	1.064548
H	0.428959	2.299711	0.124579
H	-1.062204	1.791207	-0.678165
H	1.540853	-3.461403	-0.225359
H	2.223085	0.770019	0.122860
H	-0.882662	-3.058771	-0.256305
H	3.124332	-1.526735	-0.031947
C	-2.209310	-0.675684	-0.128448
H	-2.499464	-0.142939	-1.039642
H	-2.772836	-1.608324	-0.091085
H	-2.529906	-0.051533	0.710878

***o*-xylene-1⊂OCav**

C	1.636227	-0.078273	-0.019904
C	2.171414	-1.365478	-0.025375
C	1.316722	-2.469641	-0.012103
C	-0.064980	-2.274705	0.005667
C	-0.613193	-0.986896	0.011237
C	0.253594	0.126171	-0.001710
H	2.297544	0.782299	-0.029516
H	-0.728518	-3.133188	0.015161
H	3.247999	-1.496201	-0.039495
C	-2.103136	-0.772017	0.030692
H	-2.408942	-0.195177	0.909329
H	-2.432573	-0.201441	-0.843509
H	-2.647441	-1.716038	0.041257
C	1.465498	4.163197	1.762625
C	0.979790	3.426223	2.847650
C	1.756354	2.350113	3.290356
C	3.012045	2.087748	2.752470
C	3.483432	2.883500	1.714220
C	2.705739	3.912281	1.167137
H	0.855133	4.968516	1.370430
H	3.628686	1.298240	3.161914
C	3.171992	4.667938	-0.074582
H	4.260956	4.610886	-0.093836
C	-0.308546	3.795674	3.565640
H	-0.250668	3.333503	4.551888
C	2.659213	3.900821	-1.288725
C	3.416037	2.862111	-1.847767
C	1.401696	4.149494	-1.847969
C	2.911171	2.057114	-2.862212
C	0.880130	3.400147	-2.908376
H	0.804456	4.959227	-1.445431
C	1.639539	2.317128	-3.363302
H	3.513842	1.262801	-3.282688
C	-1.538417	3.203984	2.894308
C	-2.190261	3.851613	1.838529
C	-2.077171	1.989537	3.334358
C	-3.376950	3.377969	1.271306
H	-1.752450	4.761192	1.444632
C	-3.288846	1.512970	2.840122
C	-3.935843	2.220884	1.832304
H	-3.734214	0.618938	3.257771
C	-0.432634	3.759701	-3.585820
H	-0.406892	3.288915	-4.569369
C	-4.012917	4.036188	0.049556
C	-1.636569	3.171032	-2.867158
C	-2.250624	3.826622	-1.794856
C	-2.192932	1.955120	-3.280846
C	-3.421517	3.363208	-1.188276
H	-1.799178	4.738900	-1.422758
C	-3.389744	1.487090	-2.745132
C	-4.003281	2.206475	-1.724007
H	-3.850049	0.590501	-3.140541
H	-5.075715	3.792218	0.069192
O	1.307243	1.576104	4.368840
O	-1.445426	1.287020	4.368744
O	4.800425	2.726530	1.272098
O	4.746037	2.708942	-1.445777
O	1.154885	1.532955	-4.418914
O	-1.596405	1.242614	-4.329102

O	-5.277576	1.835004	-1.285498	H	-4.593307	7.301812	-1.067803
O	-5.222037	1.843042	1.436397	H	-4.241359	5.898110	-2.083940
C	0.751367	0.339397	4.039362	C	-0.425447	5.317241	3.790239
C	-0.645952	0.196985	4.015379	H	0.508331	5.654114	4.254162
C	1.598855	-0.754197	3.827290	H	-0.499018	5.846093	2.834517
C	-1.237838	-1.047368	3.762350	C	2.790361	6.156942	-0.080310
C	1.007776	-1.983485	3.583761	H	3.118435	6.580473	-1.036069
H	2.673271	-0.635949	3.884134	H	1.703337	6.282648	-0.049024
C	-0.392915	-2.126341	3.540172	C	-1.609289	5.694512	4.679550
H	-2.314950	-1.156412	3.771511	H	-1.551047	5.183978	5.647379
C	-5.412576	0.630436	0.784633	H	-1.626400	6.772464	4.868406
C	-5.703003	-0.521362	1.520624	H	-2.561829	5.421261	4.215452
C	-5.442708	0.626802	-0.618781	C	3.425796	6.928061	1.075722
C	-6.004124	-1.672771	0.805156	H	3.179005	7.992741	1.018274
H	-5.714431	-0.491896	2.602782	H	3.075491	6.555277	2.043304
C	-5.763032	-0.528622	-1.336293	H	4.517271	6.833632	1.057842
C	-6.033300	-1.676440	-0.603238	C	-1.781577	5.639806	-4.673169
H	-5.818715	-0.504374	-2.417249	H	-1.815422	6.716598	-4.866206
C	5.160974	1.555748	0.614631	H	-1.748212	5.125160	-5.640037
C	5.676567	0.476916	1.338632	H	-2.717083	5.359297	-4.179269
C	5.131776	1.546185	-0.788882	C	7.100091	-2.546120	-0.099973
C	6.153891	-0.605788	0.611567	C	0.425677	-4.211630	-3.205515
H	5.730823	0.516633	2.419182	C	-6.543795	-3.769505	0.116985
C	5.617082	0.457471	-1.518931	C	0.539786	-4.177718	3.227816
C	6.124544	-0.615396	-0.797830	O	-6.804018	-4.976219	0.125580
H	5.627016	0.482331	-2.601233	O	0.673173	-5.392012	3.045381
C	0.610670	0.298794	-4.061452	O	7.647339	-3.653195	-0.103832
C	1.464608	-0.793510	-3.869060	O	0.564855	-5.424478	-3.017918
C	-0.785137	0.156342	-3.991303	C	6.967885	-2.226815	-2.578410
C	0.881866	-2.020658	-3.595852	C	5.692291	-2.609784	-3.311310
H	2.536510	-0.676856	-3.964361	H	5.026074	-1.743809	-3.390156
C	-1.368300	-1.085483	-3.707487	C	3.665522	-3.427599	-2.354976
C	-0.516544	-2.162583	-3.503674	C	2.828782	-3.654836	-3.613126
H	-2.445147	-1.194110	-3.680046	C	-2.075179	-4.150220	-3.266144
N	-0.770876	-3.511741	-3.268617	O	5.043965	-3.672737	-2.611047
N	1.433894	-3.287545	-3.431482	C	-6.307087	-3.461540	2.582333
N	6.726841	-1.799670	-1.209205	C	-4.882167	-3.515109	3.105542
N	1.554495	-3.251564	3.411842	H	-4.457482	-2.504936	3.148185
N	-6.367371	-2.949762	1.223074	C	-2.762244	-3.881281	2.087778
N	6.773705	-1.784251	1.013467	C	-1.957594	-4.116558	3.366706
N	-0.654167	-3.477745	3.326245	C	7.058139	-2.199261	2.377917
N	-6.412880	-2.955506	-0.999742	C	5.805152	-2.575236	3.152269
C	-0.566986	5.278582	-3.819122	C	3.750433	-3.399968	2.264220
H	-0.614931	5.814529	-2.865720	C	2.954492	-3.617435	3.550189
H	0.348771	5.618768	-4.315230	H	-2.498225	-3.719208	4.230373
C	-3.893807	5.568737	0.043176	H	-1.796519	-5.183612	3.527033
H	-4.290893	5.936681	0.995814	H	-2.326949	-4.439927	1.255427
H	-2.844875	5.880761	0.010498	H	-2.753940	-2.815844	1.833501
C	-4.651866	6.210136	-1.118328	H	-4.894410	-3.918706	4.125471
H	-5.710479	5.928150	-1.099891	H	-6.913385	-2.830989	3.238782

H	-6.747084	-4.459344	2.561963
O	5.136207	-3.643079	2.479242
O	-4.102637	-4.336325	2.234901
H	3.420517	-4.099319	1.491933
H	3.595140	-2.380783	1.899101
H	6.095451	-2.883611	4.163758
H	5.141935	-1.708078	3.243343
H	7.731079	-3.055140	2.308848
H	7.581310	-1.391568	2.899248
H	2.994723	-4.667102	3.846516
H	3.378565	-3.017397	4.360212
H	7.473927	-1.423511	-3.123062
H	7.643472	-3.081593	-2.522955
H	5.952987	-2.925911	-4.328471
H	3.521312	-2.405635	-1.993077
H	3.360023	-4.121064	-1.567475
H	2.859355	-4.706761	-3.902549
H	3.225877	-3.061032	-4.441263
H	-1.919476	-5.218337	-3.424585
H	-2.641006	-3.757916	-4.115927
C	-2.843300	-3.906707	-1.966470
H	-2.387974	-4.463329	-1.143512
H	-2.825250	-2.840286	-1.717027
C	-4.990822	-3.536941	-2.924789
H	-4.563844	-2.528449	-2.984028
H	-5.035975	-3.944065	-3.942388
C	-6.398488	-3.475491	-2.357327
H	-6.841000	-4.471588	-2.317253
H	-7.023383	-2.846276	-2.997295
O	-4.188393	-4.358602	-2.075566
C	-0.312749	1.513567	0.003875
H	-0.951445	1.678252	-0.865644
H	-0.932786	1.678713	0.886705
H	0.475465	2.265994	-0.004180
H	1.716690	-3.477511	-0.015538

***o*-xylene-2⊂OCav**

C	-2.180745	3.763744	1.749624
C	-3.362142	3.360484	1.121753
C	-3.987995	2.208995	1.614484
C	-3.419911	1.444853	2.630688
C	-2.221140	1.864419	3.201056
C	-1.611519	3.063181	2.816551
H	-1.693416	4.669329	1.407354
H	-3.914371	0.551959	2.991813
C	-0.413530	3.599125	3.582715
H	-0.429322	3.105919	4.555093
C	-3.917684	4.092123	-0.098117
H	-4.982631	3.864733	-0.151583
C	0.904432	3.216006	2.933103
C	1.622623	2.098744	3.374190
C	1.481036	3.994714	1.927221
C	2.905098	1.826733	2.903303
C	2.759014	3.752641	1.417075
H	0.922417	4.840603	1.544965
C	3.469495	2.666819	1.945668
H	3.470519	0.999300	3.313322
C	-3.260676	3.476726	-1.329039
C	-2.039415	3.941899	-1.822828
C	-3.825894	2.363097	-1.965498
C	-1.360417	3.331639	-2.882683
H	-1.595248	4.817558	-1.364447
C	-3.159982	1.693970	-2.988206
C	-1.918861	2.162842	-3.414386
H	-3.607829	0.829543	-3.462424
C	3.330152	4.594070	0.280441
H	4.415838	4.508083	0.329739
C	-0.098781	3.943020	-3.472978
C	2.882020	3.937452	-1.018219
C	1.644899	4.222113	-1.609141
C	3.674125	2.949956	-1.616859
C	1.171779	3.542285	-2.736709
H	1.027879	5.000701	-1.175650
C	3.220829	2.221134	-2.710267
C	1.961065	2.498369	-3.229195
H	3.845314	1.465166	-3.166873
H	-0.006580	3.527655	-4.477272
O	-5.257782	1.884382	1.124606
O	-5.128118	1.988126	-1.617221
O	-1.681200	1.142583	4.273244
O	1.097831	1.320712	4.414547
O	4.813049	2.505324	1.584812
O	4.975175	2.750038	-1.141296
O	1.496226	1.750292	-4.317875
O	-1.266562	1.488391	-4.459972
C	-5.425716	0.709902	0.400207
C	-5.356305	0.761191	-1.002542
C	-5.785406	-0.464091	1.067206
C	-5.653687	-0.360087	-1.783206
C	-6.059672	-1.580190	0.289504
H	-5.867767	-0.474657	2.146568
C	-6.002256	-1.527369	-1.116824
H	-5.635615	-0.292949	-2.863635
C	-0.488143	0.389770	-4.080223
C	-1.102342	-0.851664	-3.867397
C	0.905049	0.531811	-3.979200
C	-0.287624	-1.915237	-3.505886
H	-2.174027	-0.960285	-3.978054
C	1.719636	-0.540073	-3.600094
C	1.100475	-1.750659	-3.343580

H	2.792464	-0.421493	-3.525775	H	4.626227	6.618146	1.694541
C	-0.902011	0.017521	4.001643	H	3.325365	7.818606	1.642202
C	-1.534785	-1.212654	3.791031	H	3.101005	6.307995	2.534225
C	0.499574	0.103354	4.090454	C	0.111592	-4.446880	3.527478
C	-0.732567	-2.340457	3.691079	C	6.824477	-2.785830	-0.153824
H	-2.615193	-1.270163	3.744322	C	0.596944	-3.913800	-2.868957
C	1.303344	-1.039682	3.997028	C	-6.596558	-3.635184	-0.513159
C	0.667965	-2.258207	3.809101	O	0.704972	-5.097508	-2.533457
H	2.377957	-0.959568	4.104136	O	-6.878629	-4.835544	-0.568384
C	5.188443	1.394960	0.835165	O	0.198307	-5.669670	3.378030
C	5.572143	0.216117	1.480911	O	7.278318	-3.931107	-0.231381
C	5.277400	1.521790	-0.560716	C	2.557149	-3.959721	3.749599
C	6.007578	-0.835087	0.684808	C	3.174211	-3.791485	2.358185
H	5.531173	0.146831	2.560468	H	2.762621	-4.539469	1.675752
C	5.752496	0.474948	-1.357068	C	5.284901	-2.927319	3.038781
C	6.100342	-0.705320	-0.714927	C	6.608022	-2.666687	2.341545
H	5.852521	0.601446	-2.427659	C	7.005530	-2.166032	-2.581345
N	6.611450	-1.903411	-1.204346	O	4.585742	-3.969305	2.357042
N	6.457266	-2.112812	1.004375	C	-1.856088	-3.904858	-3.368594
N	1.158503	-3.561642	3.745810	C	-2.770661	-3.628457	-2.174027
N	-6.461863	-2.866633	0.633528	H	-2.406827	-4.150444	-1.285385
N	-0.565113	-3.255261	-3.243767	C	-4.801246	-3.326135	-3.378746
N	-1.049204	-3.687669	3.534417	C	-6.258911	-3.261683	-2.952033
N	-6.393333	-2.778202	-1.587654	C	-2.381187	-4.239954	3.364444
N	1.617813	-2.979907	-2.949741	C	-2.943422	-3.958846	1.967028
C	2.983346	6.089254	0.369173	C	-5.173119	-3.491562	2.647093
H	3.399055	6.577991	-0.519045	C	-6.532917	-3.420606	1.975708
H	1.900711	6.244801	0.322969	H	-6.813612	-2.609515	-3.632120
C	-0.200346	5.474774	-3.632856	H	-6.713548	-4.252648	-2.978278
H	-0.299246	5.960724	-2.656818	H	-4.376599	-2.316440	-3.424727
H	0.749598	5.824269	-4.052307	H	-4.741426	-3.763818	-4.382673
C	-1.352548	5.901727	-4.540935	H	-2.797589	-2.552894	-1.958216
H	-1.352568	6.986728	-4.684930	H	-2.323156	-3.545931	-4.289555
H	-1.269334	5.431400	-5.527172	H	-1.679864	-4.976808	-3.471180
H	-2.321830	5.620617	-4.118304	O	-4.314315	-4.329876	1.873498
C	-3.778656	5.621589	-0.022891	O	-4.084031	-4.113365	-2.427289
H	-2.726775	5.917724	0.043550	H	-4.736881	-2.488964	2.733698
H	-4.152610	6.036231	-0.965717	H	-5.307260	-3.888025	3.661296
C	-0.511629	5.115606	3.848365	H	-2.825783	-2.895659	1.723204
H	0.402415	5.420321	4.370245	H	-2.405070	-4.546343	1.219401
H	-0.527108	5.674131	2.906974	H	-2.319632	-5.313940	3.544729
C	-4.550399	6.221399	1.151709	H	-3.026734	-3.801782	4.129792
H	-5.612086	5.955226	1.101516	H	-6.965105	-4.417708	1.880777
H	-4.162065	5.860961	2.109662	H	-7.204648	-2.811015	2.587175
H	-4.476619	7.313325	1.151153	H	3.078306	-3.339842	4.482698
C	-1.733687	5.488908	4.686329	H	2.610458	-4.998075	4.080042
H	-1.745405	6.561974	4.901472	H	2.923031	-2.798051	1.976758
H	-2.665608	5.241856	4.168319	H	5.492077	-3.212432	4.078027
H	-1.732250	4.953550	5.642456	H	4.678134	-2.014175	3.056855
C	3.539739	6.745440	1.631981	H	7.169995	-3.596154	2.236592

H	7.199986	-1.974318	2.948466
H	7.653145	-3.044519	-2.568201
H	7.590266	-1.314649	-2.942229
C	5.834474	-2.393074	-3.518057
H	5.100817	-1.586174	-3.402316
H	6.197799	-2.368535	-4.554590
C	3.881941	-3.736361	-3.656361
H	3.718871	-3.151214	-4.569897
H	3.661263	-4.784044	-3.877655
C	2.984608	-3.228393	-2.527633
H	3.383777	-2.290694	-2.139140
H	2.967731	-3.950219	-1.708494
O	5.243470	-3.662283	-3.237747
C	-1.567438	0.364034	-0.099043
C	-0.530428	1.296714	-0.129871
C	0.786520	0.856229	-0.043058
C	1.097009	-0.503791	0.089770
C	0.050866	-1.447992	0.124806
C	-1.270836	-0.990750	0.025471
H	-0.743550	2.352812	-0.224072
H	1.597209	1.576904	-0.076606
C	0.310447	-2.924814	0.279465
H	0.601844	-3.168973	1.301836
H	-0.581726	-3.506584	0.040906
H	1.123566	-3.273913	-0.358085
C	2.545814	-0.889293	0.216279
H	2.892898	-0.747519	1.244863
H	2.733527	-1.928516	-0.049359
H	3.168308	-0.254855	-0.417936
H	-2.596751	0.685296	-0.173482
H	-2.079033	-1.714634	0.042039

***o*-xylene-3⊂OCav**

C	-2.135667	3.757621	2.091367
C	-3.308604	3.432426	1.403973
C	-3.987644	2.274437	1.803451
C	-3.475125	1.431470	2.785087
C	-2.276885	1.768035	3.409714
C	-1.617697	2.970201	3.125211
H	-1.604920	4.661331	1.816367
H	-4.012895	0.539955	3.082296
C	-0.417820	3.413632	3.947882
H	-0.469888	2.859811	4.885866
C	-3.806171	4.254586	0.221142
H	-4.876732	4.070071	0.127463
C	0.898429	3.021202	3.295077
C	1.546894	1.837733	3.659180
C	1.527716	3.832638	2.346170
C	2.798689	1.507676	3.148562
C	2.779868	3.537365	1.799053
H	1.025559	4.739689	2.032259
C	3.407531	2.359234	2.232980
H	3.314414	0.621471	3.493528
C	-3.143185	3.689805	-1.031814
C	-1.901290	4.147975	-1.477853
C	-3.736465	2.644836	-1.750986
C	-1.236620	3.603373	-2.581544
H	-1.435094	4.972098	-0.951264
C	-3.081974	2.031075	-2.815102
C	-1.826278	2.493146	-3.201935
H	-3.554752	1.223191	-3.359321
C	3.417148	4.430482	0.739121
H	4.495839	4.282513	0.802653
C	0.040675	4.227854	-3.119619
C	2.974357	3.912366	-0.625242
C	1.768606	4.303478	-1.220534
C	3.737370	2.947926	-1.292417
C	1.291325	3.735156	-2.406364
H	1.176159	5.073168	-0.738710
C	3.278369	2.328936	-2.448314
C	2.042254	2.696318	-2.963149
H	3.879100	1.585772	-2.954094
H	0.131149	3.889428	-4.152738
O	-5.254264	2.026327	1.265568
O	-5.056951	2.294919	-1.453505
O	-1.786352	0.937764	4.424830
O	0.972811	1.018868	4.641125
O	4.726155	2.103231	1.841399
O	5.013116	2.637782	-0.809271
O	1.561987	2.037766	-4.101900
O	-1.192727	1.872898	-4.291243
C	-5.446731	0.904200	0.467650
C	-5.338275	1.039521	-0.926320
C	-5.871673	-0.292899	1.049878
C	-5.648581	-0.019840	-1.784430
C	-6.166426	-1.346277	0.195646
H	-5.988437	-0.367954	2.123487
C	-6.056411	-1.212585	-1.202255
H	-5.597525	0.113041	-2.857683
C	-0.458896	0.723234	-3.978136
C	-1.115482	-0.509480	-3.853275
C	0.935442	0.816253	-3.845647
C	-0.340416	-1.619118	-3.543923
H	-2.187426	-0.577016	-3.990240
C	1.709383	-0.301798	-3.517612
C	1.049003	-1.506195	-3.346913
H	2.783329	-0.224356	-3.414374
C	-1.040242	-0.179961	4.045500
C	-1.697520	-1.351599	3.648915
C	0.357045	-0.147484	4.188488

C	-0.916432	-2.474407	3.414602	C	6.592955	-3.039888	-0.416991
H	-2.776376	-1.375789	3.561882	C	0.476007	-3.675465	-3.009815
C	1.137010	-1.288305	3.970340	C	-6.761768	-3.324005	-0.748050
C	0.480608	-2.445950	3.588570	O	0.541723	-4.879555	-2.739899
H	2.207771	-1.262079	4.125223	O	-7.095424	-4.504211	-0.886171
C	5.018695	1.042752	0.988363	O	-0.050016	-5.768789	2.758199
C	5.267671	-0.238086	1.497187	O	7.080765	-4.162341	-0.587736
C	5.209502	1.331855	-0.372020	C	2.277174	-4.236564	3.663217
C	5.714425	-1.205552	0.603652	C	3.329350	-3.935722	2.603163
H	5.153287	-0.440025	2.554861	H	3.027606	-4.343495	1.629298
C	5.673351	0.365007	-1.265824	C	5.582125	-4.591711	2.137188
C	5.923833	-0.900536	-0.757385	C	6.320681	-3.245099	2.036007
H	5.859551	0.617643	-2.301879	C	6.893246	-2.151828	-2.743248
N	6.461585	-2.033720	-1.358566	O	4.508822	-4.580557	3.069683
N	6.103012	-2.533347	0.780151	C	-1.958365	-3.574672	-3.589574
N	0.951026	-3.733385	3.356176	C	-2.888581	-3.403460	-2.388304
N	-6.642053	-2.629175	0.446743	H	-2.555599	-4.030458	-1.557968
N	-0.658934	-2.965052	-3.375982	C	-4.890188	-2.908526	-3.579624
N	-1.253625	-3.781349	3.072656	C	-6.345769	-2.814518	-3.152625
N	-6.474555	-2.417499	-1.760230	C	-2.598215	-4.313200	2.938659
N	1.523667	-2.770977	-3.024682	C	-3.239359	-3.947959	1.598847
C	3.146908	5.929464	0.950301	C	-5.423176	-3.469655	2.416045
H	2.076987	6.149321	0.875953	C	-6.771532	-3.260577	1.749460
H	3.440090	6.181962	1.975444	H	-6.869419	-2.097180	-3.790788
C	-0.040301	5.768599	-3.159630	H	-6.838749	-3.782867	-3.247292
H	-0.979814	6.039565	-3.654048	H	-4.419638	-1.919568	-3.532771
H	-0.096421	6.179999	-2.146678	H	-4.845763	-3.253925	-4.619742
C	1.135853	6.400646	-3.902856	H	-2.884437	-2.358027	-2.059770
H	1.020386	7.487480	-3.960560	H	-2.400413	-3.112490	-4.476079
H	2.085827	6.191955	-3.400950	H	-1.799450	-4.633378	-3.801404
H	1.207472	6.014706	-4.925924	O	-4.618384	-4.296847	1.574947
C	-3.615907	5.769575	0.401914	O	-4.213535	-3.813924	-2.706798
H	-4.080136	6.050352	1.353835	H	-4.928723	-2.504211	2.577782
H	-2.555130	6.023839	0.494309	H	-5.580800	-3.936592	3.396113
C	-0.484643	4.911762	4.307564	H	-3.118859	-2.875024	1.409200
H	-0.418754	5.530447	3.406787	H	-2.761423	-4.500867	0.786455
H	-1.473057	5.104360	4.739268	H	-2.537859	-5.396656	3.051399
C	-4.236051	6.577588	-0.737098	H	-3.197017	-3.917080	3.763539
H	-5.303965	6.355253	-0.840384	H	-7.272134	-4.216185	1.587017
H	-4.131783	7.651872	-0.555986	H	-7.405449	-2.643949	2.393060
H	-3.754980	6.351597	-1.693843	H	2.594633	-3.815357	4.621926
C	0.602048	5.329154	5.297302	H	2.189794	-5.318351	3.777780
H	0.490443	6.381370	5.577106	H	3.480862	-2.854369	2.491308
H	0.550233	4.729839	6.213190	H	5.226468	-4.908978	1.149643
H	1.601811	5.200878	4.871337	H	6.276503	-5.349078	2.507919
C	3.913693	6.803216	-0.041496	H	7.396554	-3.409300	2.145734
H	3.603069	6.602016	-1.071838	H	5.999434	-2.597792	2.852809
H	3.741049	7.865312	0.158089	H	7.545796	-3.024653	-2.802325
H	4.991799	6.618690	0.023105	H	7.479719	-1.265958	-3.004132
C	-0.109658	-4.563333	3.019585	C	5.735893	-2.290894	-3.713708

H	5.023882	-1.470755	-3.564070
H	6.118093	-2.219905	-4.741143
C	3.721100	-3.540211	-3.862076
H	3.540975	-2.877194	-4.717185
H	3.445486	-4.556814	-4.155819
C	2.889386	-3.094449	-2.657943
H	3.330669	-2.205552	-2.203167
H	2.867759	-3.879526	-1.900631
O	5.102949	-3.554683	-3.511414
C	0.984977	-2.747055	0.157136
C	-0.342416	-2.331577	0.048398
C	-0.680997	-0.973809	0.028972
C	0.347440	-0.011907	0.111907
C	1.676052	-0.437862	0.212113
C	2.000117	-1.794750	0.240569
C	-2.116032	-0.530859	-0.082763
H	-2.397361	0.111549	0.756824
H	-2.281655	0.056865	-0.992248
H	-2.801027	-1.379716	-0.100407
H	3.036947	-2.100075	0.313489
H	-1.129264	-3.072561	-0.022393
H	2.464653	0.306017	0.260612
H	1.217522	-3.806952	0.164492
C	0.017352	1.452521	0.075691
H	0.919659	2.060222	0.135042
H	-0.509813	1.714625	-0.845468
H	-0.638204	1.734901	0.904886

***o*-xylene-4⊂OCav**

C	1.669890	3.524264	2.688545
C	0.781724	2.771020	3.459111
C	1.158985	1.461885	3.783427
C	2.403195	0.950179	3.415329
C	3.281901	1.754501	2.689656
C	2.928832	3.056617	2.305048
H	1.385257	4.530659	2.408017
H	2.706892	-0.035972	3.744788
C	3.876681	3.915045	1.474901
H	4.890974	3.603602	1.726057
C	-0.524389	3.351667	3.975985
H	-0.770136	2.798383	4.882991
C	3.646353	3.558296	0.014582
C	4.451950	2.603486	-0.612384
C	2.585526	4.095031	-0.724724
C	4.166703	2.135473	-1.889700
C	2.289812	3.681387	-2.027648
H	1.959514	4.852338	-0.266089
C	3.061111	2.643005	-2.560681
H	4.795006	1.387042	-2.353459
C	-1.655792	3.091557	2.993985
C	-1.875193	3.917923	1.887628
C	-2.526035	2.013340	3.176203
C	-2.941593	3.737144	1.002219
H	-1.185657	4.735336	1.711405
C	-3.605544	1.799080	2.324642
C	-3.806215	2.661910	1.251890
H	-4.295226	0.986156	2.511945
C	1.220228	4.347421	-2.878022
H	1.431579	4.061181	-3.908976
C	-3.127928	4.632167	-0.217968
C	-0.179499	3.836530	-2.578503
C	-0.966430	4.403096	-1.570715
C	-0.752045	2.814847	-3.346909
C	-2.301434	4.050203	-1.359886
H	-0.525213	5.166730	-0.940909
C	-2.087167	2.453155	-3.189162
C	-2.852367	3.090273	-2.216928
H	-2.531841	1.715928	-3.845065
H	-4.174926	4.556390	-0.512825
O	0.343363	0.734669	4.654727
O	-2.370435	1.191050	4.299817
O	4.605068	1.344547	2.496620
O	5.587711	2.126818	0.054262
O	2.722329	2.108339	-3.812302
O	-0.002572	2.199704	-4.364088
O	-4.229399	2.859037	-2.152508
O	-4.954594	2.517251	0.465600
C	-0.467797	-0.302455	4.203877
C	-1.852070	-0.084889	4.081423
C	0.070941	-1.586175	4.069143
C	-2.738465	-1.142876	3.852277
C	-0.813842	-2.630065	3.842190
H	1.133949	-1.750804	4.195277
C	-2.202610	-2.417383	3.748065
H	-3.803330	-0.957880	3.783954
C	-5.050128	1.431562	-0.400467
C	-5.626982	0.235539	0.039732
C	-4.687625	1.612187	-1.745604
C	-5.836712	-0.757360	-0.907958
H	-5.924748	0.117757	1.074065
C	-4.911404	0.616648	-2.699171
C	-5.493423	-0.563352	-2.261102
H	-4.676399	0.792642	-3.741106
C	4.988562	0.363584	1.588245
C	4.972368	-0.987364	1.951942
C	5.568184	0.771734	0.374144
C	5.554379	-1.892477	1.073050
H	4.538032	-1.298022	2.894163
C	6.181642	-0.137464	-0.491743
C	6.173333	-1.473660	-0.119415

H	6.648913	0.209052	-1.404971	O	-2.001167	-5.868990	3.492193
C	1.960622	0.938830	-3.722847	O	6.634707	-4.907269	-0.261062
C	2.583939	-0.255225	-3.344604	O	0.894924	-4.700372	-2.775087
C	0.588887	0.979971	-4.020216	C	7.372027	-2.661850	-2.019091
C	1.795858	-1.389672	-3.265845	C	6.383174	-2.621471	-3.170010
H	3.639009	-0.280345	-3.108334	H	5.744521	-1.735558	-3.077550
C	-0.185251	-0.188903	-4.015377	C	4.257171	-3.621044	-3.575408
C	0.439253	-1.368736	-3.637491	C	3.385579	-3.157630	-2.403733
H	-1.233971	-0.156065	-4.282141	C	-1.304062	-3.168576	-4.013050
N	-0.028652	-2.675748	-3.523655	O	5.601151	-3.817306	-3.151536
N	2.093089	-2.687490	-2.868661	C	-7.150935	-2.588955	0.307586
N	6.714469	-2.606536	-0.722164	C	-6.483539	-3.796557	0.935534
N	-0.605082	-4.004309	3.761062	H	-7.241803	-4.327707	1.527786
N	-6.428193	-2.015108	-0.819969	C	-4.941011	-4.498153	2.555502
N	5.730997	-3.273832	1.157098	C	-4.197683	-4.019686	3.788968
N	-2.796728	-3.667562	3.582677	C	4.990545	-4.148823	2.049824
N	-5.924324	-1.685626	-2.961461	C	3.555411	-4.291074	1.553626
C	1.322760	5.886057	-2.812609	C	1.396316	-4.461950	2.455940
H	1.114875	6.245245	-1.799519	C	0.682945	-4.673171	3.782164
H	2.363844	6.154440	-3.023650	H	-4.709032	-3.154850	4.214922
C	-2.841258	6.116248	0.067918	H	-4.222865	-4.823102	4.530500
H	-3.445652	6.409226	0.933631	H	-5.797913	-5.092133	2.900733
H	-1.797487	6.265055	0.362231	H	-4.305223	-5.152907	1.945234
C	-3.168820	7.016689	-1.122521	H	-6.123951	-4.483786	0.158669
H	-3.007127	8.069701	-0.871861	H	-7.293386	-1.808515	1.056774
H	-2.541487	6.779679	-1.987681	H	-8.137378	-2.895072	-0.051603
H	-4.214697	6.899599	-1.427525	O	2.761629	-4.822647	2.606178
C	-0.406854	4.836014	4.372481	O	-5.415515	-3.391349	1.789295
H	0.455803	4.935596	5.040963	H	0.912639	-5.044184	1.659015
H	-0.190572	5.458283	3.498265	H	1.325898	-3.403544	2.183683
C	3.781300	5.424492	1.754756	H	3.182599	-3.298092	1.273848
H	4.466989	5.927751	1.064060	H	3.513938	-4.929945	0.659980
H	2.782215	5.806088	1.520511	H	5.507010	-5.108083	2.094020
C	-1.664802	5.355108	5.067306	H	4.989735	-3.708710	3.049557
H	-1.534302	6.394035	5.385793	H	0.507577	-5.728993	3.991783
H	-2.532439	5.316110	4.401287	H	1.284679	-4.251517	4.592260
H	-1.898794	4.757193	5.955203	H	8.063449	-1.818849	-2.097006
C	4.144393	5.783260	3.195188	H	7.952506	-3.585648	-2.049148
H	4.125086	6.867361	3.344189	H	6.931866	-2.545045	-4.118167
H	3.445325	5.335535	3.908130	H	3.891491	-4.579302	-3.954443
H	5.149670	5.426768	3.445987	H	4.205973	-2.889939	-4.391897
C	0.399038	6.585193	-3.808986	H	3.872302	-2.338157	-1.870240
H	-0.654066	6.379886	-3.593733	H	3.225725	-3.972653	-1.695017
H	0.541511	7.669885	-3.776281	H	-1.187811	-4.231206	-4.233640
H	0.601264	6.249974	-4.832513	H	-1.519778	-2.648628	-4.950676
C	6.380149	-3.731984	0.016316	C	-2.448858	-2.957706	-3.023468
C	0.975625	-3.492681	-3.022706	H	-2.348405	-3.638576	-2.176993
C	-6.477948	-2.597705	-2.077919	H	-2.440274	-1.930157	-2.638472
C	-1.815730	-4.651253	3.587331	C	-4.133660	-2.241266	-4.553021
O	-6.958661	-3.702759	-2.351489	H	-3.580793	-1.310953	-4.379251

H	-3.960359	-2.547961	-5.591636
C	-5.622566	-2.011077	-4.345501
H	-6.188848	-2.912746	-4.582702
H	-5.967495	-1.204830	-4.999042
O	-3.697764	-3.249964	-3.641185
C	-1.838469	-0.543664	0.218753
C	-0.503448	-0.138457	0.103865
C	0.508665	-1.119205	0.039916
C	0.144813	-2.470284	0.045692
C	-1.187197	-2.864441	0.190857
C	-2.185097	-1.894841	0.287151
C	1.957728	-0.712796	0.033279
H	2.229973	-0.284658	1.003246
H	2.611569	-1.567250	-0.146428
H	2.174178	0.051791	-0.716718
H	-1.439198	-3.920131	0.228721
H	-2.614438	0.212385	0.264744
H	0.919390	-3.223235	-0.052342
C	-0.147522	1.321982	0.043275
H	0.502926	1.615598	0.873786
H	0.394229	1.556084	-0.879011
H	-1.041344	1.943720	0.076398
H	-3.223329	-2.184544	0.415826

***o*-xylene-5⊂OCav**

C	2.281524	4.046545	-1.048177
C	3.271074	3.734456	-0.112325
C	4.222950	2.776422	-0.479384
C	4.155046	2.112610	-1.701525
C	3.130903	2.430175	-2.589501
C	2.206404	3.439683	-2.303897
H	1.536450	4.790735	-0.789584
H	4.891062	1.360470	-1.957142
C	1.177320	3.880265	-3.330957
H	1.496043	3.461916	-4.286134
C	3.257228	4.347904	1.279682
H	4.236190	4.163997	1.723311
C	-0.188035	3.285037	-3.037016
C	-0.544686	2.046489	-3.583671
C	-1.148495	3.961609	-2.280059
C	-1.835626	1.537323	-3.449427
C	-2.453614	3.491716	-2.121057
H	-0.879248	4.908752	-1.829808
C	-2.779589	2.273349	-2.734748
H	-2.117111	0.621692	-3.954473
C	2.238897	3.572809	2.108984
C	0.923576	4.010256	2.281801
C	2.604661	2.361799	2.713601
C	-0.010887	3.313257	3.053432
H	0.626071	4.949102	1.831893
C	1.688989	1.606231	3.442303
C	0.390267	2.088633	3.601441
H	2.000416	0.697011	3.941038
C	-3.490591	4.239006	-1.286636
H	-4.466920	4.020786	-1.720597
C	-1.396757	3.869124	3.343245
C	-3.474028	3.633059	0.110391
C	-2.520170	4.008764	1.060365
C	-4.363734	2.611432	0.463408
C	-2.410660	3.395945	2.312175
H	-1.823281	4.801131	0.811380
C	-4.255914	1.939160	1.676771
C	-3.266120	2.321746	2.578694
H	-4.947091	1.142944	1.923853
H	-1.705754	3.441652	4.297721
O	5.294633	2.513658	0.384454
O	3.962766	2.045488	2.738278
O	3.059785	1.761385	-3.820991
O	0.362792	1.416753	-4.441661
O	-4.125845	1.913276	-2.770526
O	-5.428518	2.310973	-0.396439
O	-3.171014	1.654893	3.809239
O	-0.485374	1.433056	4.474181
C	5.326626	1.239710	0.945429
C	4.555201	0.971051	2.091162
C	6.193848	0.287135	0.404003
C	4.575018	-0.294293	2.685354
C	6.239893	-0.953858	1.024392
H	6.806829	0.533998	-0.453898
C	5.410828	-1.249127	2.121552
H	3.992063	-0.495546	3.575596
C	-1.178047	0.298388	4.063446
C	-0.542556	-0.948213	4.100004
C	-2.550778	0.404590	3.774259
C	-1.317389	-2.070052	3.844705
H	0.507351	-1.021946	4.354097
C	-3.329907	-0.732104	3.532213
C	-2.693563	-1.963471	3.571054
H	-4.390844	-0.637775	3.337581
C	2.486771	0.487642	-3.769551
C	3.317577	-0.611657	-3.529946
C	1.112323	0.322196	-4.019386
C	2.732542	-1.867583	-3.528322
H	4.376880	-0.468257	-3.357665
C	0.527137	-0.949444	-4.010476
C	1.353689	-2.033333	-3.755663
H	-0.526373	-1.069857	-4.229908
C	-4.666813	0.811357	-2.124560
C	-4.645888	-0.448888	-2.729001
C	-5.430203	1.041805	-0.965905

C	-5.441343	-1.436651	-2.164210	C	-1.147133	-3.904414	-2.843730
H	-4.069087	-0.621555	-3.628968	H	-0.845885	-4.544539	-2.003473
C	-6.263926	0.057914	-0.428597	C	-3.481962	-3.822221	-2.478426
C	-6.276178	-1.177376	-1.062816	C	-4.718979	-3.541023	-3.329228
H	-6.876892	0.275866	0.437134	C	-7.885348	-2.591020	0.306523
N	-7.016706	-2.334699	-0.831021	O	-2.434250	-4.261736	-3.331437
N	-5.668878	-2.757723	-2.553342	C	0.287745	-4.010216	4.142551
N	1.097995	-3.401629	-3.721692	C	1.224788	-3.861258	2.952916
N	7.016027	-2.084800	0.782720	H	0.914554	-4.517484	2.127811
N	-1.010733	-3.428598	3.857802	C	3.539863	-3.699828	2.512131
N	3.272272	-3.138527	-3.362773	C	4.797144	-3.377574	3.314889
N	5.687548	-2.562679	2.503099	C	4.664998	-3.463759	-3.121233
N	-3.179918	-3.258302	3.425625	C	5.017336	-3.493755	-1.633661
C	-3.322683	5.767784	-1.285947	C	7.224807	-2.563759	-1.628158
H	-4.074499	6.179533	-0.603530	C	7.945615	-2.279907	-0.318360
H	-2.349985	6.055683	-0.873783	H	4.525293	-2.816330	4.211762
C	-1.404738	5.401087	3.512141	H	5.337059	-4.275127	3.617606
H	-0.618813	5.663180	4.229452	H	3.760372	-4.436820	1.726757
H	-1.141534	5.897665	2.572632	H	3.196785	-2.783078	2.021553
C	-2.752763	5.926264	4.003784	H	1.188370	-2.828155	2.587680
H	-2.715260	7.008188	4.164582	H	0.719669	-3.497273	5.006646
H	-3.547552	5.723451	3.279103	H	0.132906	-5.057862	4.403716
H	-3.036750	5.454399	4.951074	O	6.412481	-3.730966	-1.478438
C	3.041785	5.870974	1.265531	O	2.537406	-4.174178	3.400680
H	3.784457	6.300493	0.584046	H	6.614157	-1.701897	-1.918477
H	2.063052	6.124596	0.845350	H	7.966847	-2.734292	-2.415895
C	1.142595	5.412243	-3.499234	H	4.722687	-2.556994	-1.147783
H	0.349013	5.652775	-4.215740	H	4.494798	-4.317656	-1.139700
H	0.867117	5.900340	-2.558887	H	4.866829	-4.439794	-3.566877
C	3.187101	6.501552	2.650081	H	5.277171	-2.721200	-3.638826
H	4.170516	6.280160	3.079505	H	8.583903	-3.123047	-0.049133
H	3.080688	7.589530	2.596538	H	8.573088	-1.390060	-0.418554
H	2.429111	6.125485	3.343953	H	-0.609729	-3.567755	-4.891094
C	2.475320	5.973952	-3.991987	H	0.027316	-5.087604	-4.237445
H	2.407830	7.054290	-4.153579	H	-1.154659	-2.866632	-2.489582
H	3.275712	5.793736	-3.267463	H	-3.187280	-2.894890	-1.974920
H	2.771780	5.509346	-4.939034	H	-3.706943	-4.568257	-1.702929
C	-3.495038	6.381729	-2.674794	H	-5.213345	-4.457155	-3.653100
H	-4.472603	6.124523	-3.097581	H	-4.432249	-2.969915	-4.214912
H	-3.424108	7.473012	-2.630887	H	-8.479148	-3.472462	0.060006
H	-2.728313	6.024712	-3.369169	H	-8.562948	-1.744024	0.442275
C	2.269338	-4.091423	-3.445598	C	-7.080680	-2.831010	1.575575
C	-6.584418	-3.341694	-1.683606	H	-6.529543	-1.924479	1.848558
C	-2.146230	-4.170917	3.570418	H	-7.763014	-3.074472	2.397883
C	6.624986	-3.109317	1.633497	C	-4.814648	-3.598425	1.600360
O	-2.227072	-5.399885	3.479225	H	-4.210136	-4.354933	1.091666
O	7.046556	-4.268953	1.644026	H	-4.559097	-2.615074	1.184878
O	2.401158	-5.313717	-3.324728	C	-4.542106	-3.635224	3.104264
O	-6.958768	-4.516952	-1.696264	H	-4.711033	-4.643574	3.485933
C	-0.173202	-4.040518	-4.006360	H	-5.216357	-2.954010	3.629392

O	-6.177837	-3.916048	1.349813
C	1.619947	0.144959	-0.091545
C	0.227437	0.067834	0.007310
C	-0.397710	-1.197810	-0.011219
C	0.397222	-2.342518	-0.132628
C	1.787121	-2.254814	-0.244275
C	2.402604	-1.004130	-0.218037
H	-0.080569	-3.317497	-0.135391
H	3.481130	-0.915393	-0.297394
C	-0.594945	1.315591	0.152196
H	-1.109223	1.333905	1.117105
H	0.026402	2.210030	0.086498
H	-1.369138	1.377052	-0.617063
H	2.378896	-3.158258	-0.345744
H	2.093442	1.120052	-0.068220
C	-1.897968	-1.306020	0.085938
H	-2.283793	-0.772640	0.960324
H	-2.386524	-0.860532	-0.789998
H	-2.214053	-2.349484	0.153471

***m*-xylene⊂OCav**

C	-1.904029	-0.035393	0.028840
C	-2.120834	-1.414399	0.020161
C	-1.038749	-2.307504	0.001500
C	0.252902	-1.774011	-0.007567
C	0.490097	-0.394112	-0.000788
C	-0.605900	0.477901	0.018004
H	-2.751129	0.639429	0.041655
H	-0.442877	1.552401	0.022495
H	1.101493	-2.451258	-0.022891
H	-3.136089	-1.795357	0.026788
C	1.904912	0.111745	-0.020748
H	2.427307	-0.222295	-0.922067
H	1.942169	1.201603	0.005245
H	2.470280	-0.267675	0.835347
C	-1.229836	-3.803703	-0.008061
H	-0.845896	-4.266122	0.905500
H	-2.282505	-4.072962	-0.095234
H	-0.693868	-4.270738	-0.838687
C	-1.342305	4.213907	-1.701300
C	-0.883757	3.479480	-2.797710
C	-1.708194	2.456269	-3.277429
C	-2.978183	2.235515	-2.752930
C	-3.418753	3.026950	-1.696011
C	-2.600926	4.011165	-1.128732
H	-0.701214	4.984395	-1.288651
H	-3.624029	1.480762	-3.183387
C	-3.043103	4.772447	0.114439
H	-4.132189	4.737712	0.144177
C	0.419430	3.812058	-3.501209
H	0.351673	3.365190	-4.493550
C	-2.531140	3.985990	1.312719
C	-3.314242	2.980046	1.892552
C	-1.248943	4.185893	1.831550
C	-2.821155	2.169634	2.910574
C	-0.735567	3.429382	2.888781
H	-0.631502	4.969171	1.408008
C	-1.530100	2.389655	3.382783
H	-3.443191	1.403070	3.354752
C	1.628423	3.172573	-2.837121
C	2.314653	3.794655	-1.790413
C	2.145759	1.964620	-3.319044
C	3.520442	3.306054	-1.279537
H	1.901979	4.705048	-1.371980
C	3.368340	1.468607	-2.873820
C	4.049714	2.152060	-1.870598
H	3.786165	0.572888	-3.315988
C	0.598893	3.752123	3.536108
H	0.576240	3.293603	4.525182
C	4.196913	3.939573	-0.070165
C	1.774131	3.120014	2.807744
C	2.404449	3.754304	1.734981
C	2.321224	1.911094	3.253164
C	3.587759	3.279990	1.162627
H	1.969273	4.668152	1.347783
C	3.522933	1.426626	2.743820
C	4.151796	2.124351	1.715489
H	3.965926	0.527669	3.154114
H	5.249651	3.658660	-0.100922
O	-1.281766	1.697720	-4.376463
O	1.480654	1.304783	-4.360904
O	-4.739100	2.913110	-1.245589
O	-4.653776	2.871680	1.502380
O	-1.052229	1.616789	4.450138
O	1.709582	1.238231	4.319104
O	5.414201	1.719020	1.267288
O	5.330889	1.740357	-1.487863
C	-0.761664	0.437994	-4.077046
C	0.630300	0.247133	-4.032153
C	-1.648655	-0.632688	-3.918683
C	1.173175	-1.021719	-3.794498
C	-1.104641	-1.888390	-3.706607
H	-2.716799	-0.474402	-3.995604
C	0.288070	-2.076601	-3.623262
H	2.246390	-1.165986	-3.782967
C	5.485268	0.526884	-0.826522
C	5.722866	-0.634248	-1.567468
C	5.529783	0.516323	0.577969
C	5.983560	-1.800265	-0.861317
H	5.723686	-0.599814	-2.649565

C	5.813562	-0.655943	1.284660	H	-4.346431	6.966120	-1.009691
C	6.029053	-1.811267	0.545732	C	2.021113	5.597761	4.589690
H	5.881000	-0.637719	2.365093	H	2.092411	6.674718	4.771355
C	-5.121804	1.754464	-0.578029	H	1.980338	5.094204	5.562103
C	-5.653720	0.684138	-1.303138	H	2.941880	5.281711	4.089675
C	-5.074749	1.731929	0.826085	C	-7.075957	-2.343901	0.124391
C	-6.122815	-0.404571	-0.581209	C	-0.492073	-4.180951	3.441975
H	-5.719465	0.734153	-2.382604	C	6.428181	-3.923328	-0.188791
C	-5.556527	0.637498	1.550119	C	-0.714904	-4.109289	-3.430136
C	-6.073666	-0.428267	0.826850	O	6.623365	-5.141921	-0.205058
H	-5.550252	0.653089	2.632494	O	-0.890057	-5.325719	-3.303424
C	-0.539462	0.362517	4.118057	O	-7.642144	-3.440998	0.125532
C	-1.426150	-0.712616	3.989550	O	-0.665745	-5.396684	3.303870
C	0.850893	0.178942	4.018550	C	-6.942393	-2.019948	2.607080
C	-0.883772	-1.964564	3.750625	C	-5.688359	-2.391271	3.380783
H	-2.491199	-0.561456	4.111226	H	-4.998332	-1.541161	3.418176
C	1.390835	-1.085377	3.753363	C	-3.665037	-3.350486	2.550168
C	0.505669	-2.144323	3.611966	C	-2.877703	-3.536417	3.847139
H	2.463564	-1.222833	3.698781	C	2.010582	-4.159854	3.316998
N	0.719613	-3.506178	3.419667	O	-5.062578	-3.512253	2.755910
N	-1.468787	-3.224910	3.670377	C	6.139778	-3.597756	-2.644543
N	-6.670475	-1.616069	1.235682	C	4.697287	-3.556657	-3.117548
N	-1.694389	-3.145176	-3.608245	H	4.336230	-2.521190	-3.148248
N	6.281943	-3.090635	-1.289759	C	2.557767	-3.851276	-2.117463
N	-6.749764	-1.577667	-0.987054	C	1.790626	-4.105503	-3.418758
N	0.501030	-3.440920	-3.446346	C	-7.116807	-1.934888	-2.349095
N	6.354472	-3.108478	0.932832	C	-5.921123	-2.303846	-3.211683
C	0.786484	5.268718	3.751495	C	-3.879488	-3.293008	-2.458774
H	0.842296	5.793168	2.792166	C	-3.106910	-3.450299	-3.768101
H	-0.112065	5.644074	4.253726	H	2.370435	-3.747366	-4.273955
C	4.131283	5.475664	-0.055630	H	1.612341	-5.173335	-3.552023
H	4.541204	5.834700	-1.006309	H	2.092840	-4.392554	-1.289541
H	3.094399	5.824830	-0.020065	H	2.555654	-2.780901	-1.873445
C	4.911322	6.081725	1.110244	H	4.651994	-3.959705	-4.136734
H	5.958683	5.760420	1.091793	H	6.761705	-3.009334	-3.324858
H	4.893570	7.175129	1.066646	H	6.513339	-4.622413	-2.637506
H	4.487417	5.779015	2.073164	O	-5.277833	-3.453314	-2.660038
C	0.588189	5.331988	-3.708137	O	3.896464	-4.324814	-2.218398
H	-0.332829	5.704132	-4.170694	H	-3.574294	-4.068776	-1.754352
H	0.676523	5.848008	-2.746728	H	-3.663152	-2.315009	-2.016220
C	-2.629807	6.253275	0.122539	H	-6.274284	-2.518641	-4.227358
H	-2.945821	6.681365	1.080373	H	-5.220199	-1.464013	-3.270408
H	-1.540694	6.356895	0.087007	H	-7.803074	-2.780084	-2.277214
C	1.785516	5.679867	-4.591435	H	-7.650979	-1.095765	-2.806229
H	1.713804	5.179612	-5.563725	H	-3.187184	-4.474789	-4.135780
H	1.835989	6.758358	-4.771134	H	-3.511698	-2.779921	-4.531261
H	2.728175	5.373869	-4.127723	H	-7.459069	-1.204772	3.123849
C	-3.253338	7.038776	-1.030269	H	-7.618716	-2.874395	2.553476
H	-2.985153	8.098225	-0.972127	H	-5.973228	-2.637394	4.410961
H	-2.912838	6.660236	-1.999203	H	-3.450875	-2.362527	2.128894

H	-3.366749	-4.109546	1.825290
H	-2.950371	-4.568558	4.194152
H	-3.270831	-2.882835	4.630620
H	1.850157	-5.229245	3.460267
H	2.636663	-3.795816	4.136189
C	2.695843	-3.898568	1.972465
H	2.179973	-4.435773	1.172410
H	2.677490	-2.826488	1.735408
C	4.889861	-3.612250	2.846818
H	4.528103	-2.578807	2.912649
H	4.908110	-4.028898	3.861347
C	6.300228	-3.639038	2.285094
H	6.676771	-4.661540	2.237147
H	6.961179	-3.058606	2.934733
O	4.037995	-4.371923	1.988233

***m*-xylene-1⊂OCav**

C	1.204673	0.796604	0.061062
C	1.264712	-0.601637	0.074604
C	0.067595	-1.325193	0.003344
C	-1.174216	-0.682946	-0.077131
C	-1.205747	0.716298	-0.082242
C	-0.023431	1.450236	-0.015112
H	-0.059202	2.532586	-0.021790
C	2.187957	4.067679	1.234248
C	1.964191	3.427081	2.455379
C	2.842099	2.396129	2.810059
C	3.956334	2.093400	2.032930
C	4.164793	2.784115	0.841242
C	3.267650	3.764444	0.398899
H	1.485103	4.829268	0.915653
H	4.654388	1.329598	2.351861
C	3.400429	4.410930	-0.976540
H	4.429886	4.260814	-1.302838
C	0.841308	3.857172	3.384497
H	1.070444	3.425786	4.359550
C	2.506676	3.630377	-1.934261
C	2.992220	2.467824	-2.548216
C	1.185137	3.997086	-2.204669
C	2.177462	1.665474	-3.341087
C	0.350182	3.251332	-3.044882
H	0.796636	4.906645	-1.763448
C	0.857106	2.052554	-3.558981
H	2.572333	0.784229	-3.829262
C	-0.506120	3.279089	2.977436
C	-1.384413	3.950393	2.119008
C	-0.937762	2.063828	3.518745
C	-2.680170	3.496622	1.857712
H	-1.053244	4.874756	1.661356
C	-2.229624	1.587167	3.306454
C	-3.092130	2.318341	2.495927
H	-2.567735	0.691793	3.811217
C	-1.030555	3.732273	-3.462522
H	-1.230463	3.272816	-4.431345
C	-3.619643	4.200885	0.884767
C	-2.124287	3.242540	-2.526680
C	-2.387751	3.888662	-1.316958
C	-2.934663	2.152534	-2.861927
C	-3.446170	3.534367	-0.475981
H	-1.738032	4.702768	-1.015911
C	-4.026339	1.792054	-2.076824
C	-4.278947	2.490504	-0.898517
H	-4.672675	0.977849	-2.379901
H	-4.638663	3.997428	1.215000
O	2.631243	1.686493	4.002677
O	-0.103035	1.394310	4.420503
O	5.321622	2.523287	0.093954
O	4.362676	2.216075	-2.458327
O	0.066287	1.313411	-4.445671
O	-2.680471	1.437358	-4.043044
O	-5.417430	2.172448	-0.145543
O	-4.443598	1.977777	2.411427
C	2.032247	0.433390	3.841666
C	0.639847	0.298338	3.985457
C	2.846118	-0.678859	3.602567
C	0.018208	-0.946781	3.837460
C	2.225445	-1.911825	3.474085
H	3.920235	-0.564668	3.527875
C	0.826569	-2.040661	3.560414
H	-1.051780	-1.046348	3.967271
C	-4.869753	0.819721	1.776137
C	-4.900114	-0.390478	2.474085
C	-5.423864	0.931981	0.489635
C	-5.485982	-1.478569	1.840912
H	-4.511169	-0.448084	3.481723
C	-6.067487	-0.149032	-0.120415
C	-6.099482	-1.348093	0.578837
H	-6.527587	-0.032631	-1.093787
C	5.406496	1.273121	-0.516549
C	6.116715	0.246722	0.113867
C	4.861174	1.100622	-1.800169
C	6.223531	-0.961658	-0.561681
H	6.569116	0.410703	1.084072
C	4.968088	-0.118586	-2.474148
C	5.619873	-1.155485	-1.820236
H	4.585420	-0.219272	-3.480651
C	-0.605400	0.178746	-3.995030
C	0.093101	-1.022118	-3.825109
C	-2.004565	0.226938	-3.861005
C	-0.646402	-2.160941	-3.536852

H	1.168232	-1.054781	-3.946211	H	3.507729	-2.157866	-3.589801
C	-2.747971	-0.930845	-3.609598	C	1.969533	-3.602272	-2.310688
C	-2.051145	-2.120080	-3.459207	C	1.078259	-4.024550	-3.481689
H	-3.827646	-0.883865	-3.543325	C	-3.861720	-3.889396	-3.217023
N	-2.486042	-3.425839	-3.253056	O	3.338770	-3.930269	-2.530262
N	-0.267114	-3.489350	-3.349860	C	-5.180592	-3.408478	3.474579
N	5.881202	-2.467582	-2.200432	C	-3.671911	-3.352896	3.639042
N	2.741647	-3.190016	3.284751	H	-3.313545	-2.317080	3.630140
N	-5.665691	-2.796416	2.247161	C	-1.694413	-3.675288	2.363941
N	6.880624	-2.144770	-0.240548	C	-0.775544	-4.012498	3.541268
N	0.531840	-3.393337	3.394670	C	7.728856	-2.390201	0.915580
N	-6.680771	-2.576246	0.281215	C	6.937265	-2.727598	2.169582
C	-1.088921	5.259403	-3.671373	C	4.737310	-3.599748	1.854683
H	-0.927752	5.787563	-2.726136	C	4.143372	-3.567346	3.262864
H	-0.255835	5.538677	-4.326020	H	-1.211811	-3.659516	4.479393
C	-3.447086	5.728231	0.845636	H	-0.620821	-5.089257	3.620057
H	-3.538738	6.101783	1.871740	H	-1.357352	-4.192190	1.462235
H	-2.441533	6.000940	0.509858	H	-1.662917	-2.597296	2.174529
C	-4.481223	6.407010	-0.051344	H	-3.435812	-3.776816	4.623268
H	-5.500501	6.163882	0.268827	H	-5.638760	-2.912064	4.336307
H	-4.369593	7.495309	-0.023218	H	-5.521746	-4.444554	3.458786
H	-4.374561	6.086949	-1.092937	O	6.131178	-3.885383	1.939604
C	0.814175	5.388795	3.568566	O	-3.038342	-4.089633	2.593890
H	1.829816	5.704475	3.831858	H	4.274856	-4.396801	1.265852
H	0.575499	5.889542	2.624523	H	4.566581	-2.649571	1.342113
C	3.138726	5.925889	-0.972391	H	7.638506	-2.940735	2.983278
H	3.204000	6.279513	-2.007464	H	6.317754	-1.876021	2.471592
H	2.120523	6.148006	-0.637357	H	8.382137	-3.224289	0.653207
C	-0.162258	5.840057	4.653897	H	8.349902	-1.506743	1.090704
H	0.066308	5.358329	5.611204	H	4.234175	-4.554094	3.721701
H	-0.106784	6.923242	4.801004	H	4.689694	-2.853325	3.884031
H	-1.195375	5.590759	4.393894	H	5.867087	-2.625401	-4.286995
C	4.136011	6.684478	-0.097993	H	5.839772	-4.145098	-3.380438
H	3.959575	7.763376	-0.148366	H	3.720754	-3.620812	-4.563330
H	4.054961	6.380849	0.950574	H	1.865537	-2.525360	-2.143035
H	5.165850	6.495517	-0.420879	H	1.665580	-4.122127	-1.398950
C	-2.409626	5.715542	-4.289939	H	0.994859	-5.110571	-3.536633
H	-2.400458	6.793861	-4.476901	H	1.493291	-3.664888	-4.426876
H	-2.592247	5.209532	-5.244591	H	-3.892421	-4.888978	-3.655435
H	-3.255985	5.498019	-3.630739	H	-4.454129	-3.223581	-3.849217
C	6.643998	-3.094612	-1.224097	C	-4.445535	-3.929349	-1.804657
C	-1.395828	-4.277451	-3.170662	H	-3.930694	-4.683409	-1.202745
C	-6.385199	-3.489848	1.282966	H	-4.331337	-2.959768	-1.313752
C	1.707626	-4.110360	3.220667	C	-6.698169	-3.202121	-2.118835
O	-6.723508	-4.676620	1.324620	H	-6.134094	-2.317462	-2.433863
O	1.817912	-5.330638	3.063586	H	-7.384110	-3.471343	-2.929003
O	7.058762	-4.257428	-1.242903	C	-7.510490	-2.896759	-0.869979
O	-1.428647	-5.499974	-2.996225	H	-8.108402	-3.767012	-0.593579
C	5.437127	-3.131842	-3.416589	H	-8.187853	-2.058879	-1.060096
C	3.928215	-3.170127	-3.584489	O	-5.819530	-4.302486	-1.872455

H	0.103236	-2.411598	0.011031
C	2.594801	-1.301067	0.145631
H	3.098259	-1.297007	-0.825415
H	3.261344	-0.799839	0.850712
H	2.478531	-2.340986	0.456646
C	-2.456068	-1.467906	-0.140379
H	-2.968531	-1.471166	0.825977
H	-3.146309	-1.028280	-0.863645
H	-2.270982	-2.505679	-0.424317
H	2.121332	1.369356	0.106129
H	-2.157995	1.226800	-0.134604

***m*-xylene-2⊂OCav**

C	1.265836	-0.840130	-0.017709
C	1.303887	-2.238814	-0.015766
C	0.131413	-2.986618	0.002181
C	-1.094961	-2.330689	0.018268
C	-1.164019	-0.933125	0.016223
C	0.024679	-0.198482	-0.001760
H	-0.016955	0.888496	-0.003360
H	-2.009785	-2.901391	0.032544
H	2.259998	-2.737442	-0.027900
C	-2.498651	-0.252170	0.033773
H	-3.072697	-0.534760	0.920300
H	-2.394198	0.833131	0.031614
H	-3.096659	-0.536048	-0.836381
C	1.773772	4.069045	1.719346
C	1.231310	3.334934	2.776527
C	1.960289	2.229890	3.233022
C	3.233934	1.945815	2.746563
C	3.763800	2.740811	1.732121
C	3.029790	3.792390	1.171346
H	1.203306	4.898766	1.318172
H	3.808001	1.129317	3.166324
C	3.544933	4.537973	-0.054590
H	4.629060	4.429268	-0.069296
C	-0.043412	3.762220	3.484051
H	-0.014299	3.291318	4.467115
C	2.993511	3.793365	-1.263339
C	3.709983	2.737260	-1.840052
C	1.724249	4.071575	-1.779398
C	3.153155	1.941976	-2.839122
C	1.152311	3.333815	-2.818964
H	1.164052	4.901272	-1.364868
C	1.867090	2.226802	-3.292105
H	3.715742	1.124815	-3.272918
C	-1.309928	3.252689	2.814886
C	-1.926762	3.948518	1.772362
C	-1.953343	2.105124	3.291418
C	-3.175981	3.590363	1.258036
H	-1.421681	4.813630	1.359253
C	-3.217963	1.736116	2.837718
C	-3.823941	2.491433	1.836606
H	-3.727270	0.885982	3.273979
C	-0.144802	3.755395	-3.487810
H	-0.143860	3.283853	-4.471033
C	-3.773666	4.296691	0.048659
C	-1.387986	3.241435	-2.779677
C	-1.974381	3.935711	-1.719662
C	-2.046551	2.095610	-3.239950
C	-3.211406	3.581821	-1.174461
H	-1.459441	4.801947	-1.320640
C	-3.299377	1.729651	-2.753489
C	-3.877929	2.486812	-1.737077
H	-3.821609	0.880640	-3.176296
H	-4.849254	4.120982	0.063546
O	1.442850	1.464788	4.287608
O	-1.359336	1.376581	4.331417
O	5.086481	2.555852	1.309108
O	5.042838	2.551351	-1.451236
O	1.320150	1.459554	-4.330186
O	-1.481905	1.366538	-4.295669
O	-5.186520	2.218231	-1.315215
O	-5.142143	2.221221	1.447589
C	0.775706	0.284106	3.954057
C	-0.629939	0.239818	3.976619
C	1.530128	-0.872425	3.720836
C	-1.317527	-0.961831	3.766132
C	0.842910	-2.059083	3.518848
H	2.612059	-0.830634	3.736365
C	-0.564291	-2.103633	3.541341
H	-2.398921	-0.987829	3.815869
C	-5.426896	1.037046	0.774463
C	-5.777463	-0.102100	1.505254
C	-5.449485	1.035657	-0.630434
C	-6.112559	-1.243080	0.789426
H	-5.782568	-0.076039	2.587655
C	-5.821239	-0.105421	-1.347699
C	-6.133386	-1.244968	-0.619408
H	-5.859028	-0.081666	-2.429560
C	5.423544	1.390434	0.627616
C	5.865880	0.276484	1.347420
C	5.400854	1.387879	-0.777269
C	6.249131	-0.842045	0.620656
H	5.903199	0.304761	2.429152
C	5.820621	0.271327	-1.506490
C	6.227242	-0.844350	-0.787962
H	5.823653	0.294854	-2.588978
C	0.664841	0.278118	-3.976351
C	1.427043	-0.877239	-3.762999

C	-0.740823	0.231884	-3.958780	C	6.817229	-2.556148	-2.572116
C	0.747278	-2.064371	-3.538993	C	5.467586	-2.819123	-3.220766
H	2.507955	-0.834286	-3.810361	H	4.867868	-1.901626	-3.232454
C	-1.420653	-0.969739	-3.724797	C	3.412483	-3.565604	-2.262911
C	-0.659980	-2.110240	-3.519405	C	2.578894	-3.815320	-3.521564
H	-2.503064	-0.996706	-3.742135	C	-2.372860	-3.976815	-3.448765
N	-1.023071	-3.443137	-3.350181	O	4.789013	-3.842105	-2.490440
N	1.200830	-3.370647	-3.382096	C	-6.588565	-2.989782	2.575964
N	6.700604	-2.085877	-1.200590	C	-5.223703	-3.165664	3.222109
N	1.290759	-3.365913	3.350815	H	-4.686312	-2.210328	3.235648
N	-6.505714	-2.511836	1.204332	C	-3.124475	-3.777689	2.263395
N	6.734797	-2.082279	1.022720	C	-2.280050	-3.968422	3.525064
N	-0.933132	-3.436682	3.386676	C	6.884410	-2.550828	2.391692
N	-6.537626	-2.515065	-1.019292	C	5.550488	-2.810726	3.073370
C	-0.197039	5.279980	-3.726183	C	3.471603	-3.560836	2.170939
H	-0.223603	5.820314	-2.774363	C	2.672167	-3.810812	3.451682
H	0.738924	5.569507	-4.216749	H	-2.754207	-3.462589	4.371414
C	-3.556283	5.818694	0.043168	H	-2.194059	-5.029025	3.766412
H	-3.944753	6.213123	0.988851	H	-2.755189	-4.425081	1.464357
H	-2.489818	6.065140	0.026052	H	-3.066341	-2.736903	1.925738
C	-4.253505	6.502291	-1.132338	H	-5.364973	-3.486761	4.261413
H	-5.328054	6.288088	-1.131196	H	-7.181948	-2.285000	3.165975
H	-4.126488	7.588275	-1.084847	H	-7.119130	-3.942565	2.546021
H	-3.847131	6.160163	-2.089631	O	4.854070	-3.835326	2.362245
C	-0.077598	5.287110	3.723436	O	-4.481161	-4.140785	2.488809
H	0.875118	5.569060	4.185514	H	3.125661	-4.227826	1.377558
H	-0.128231	5.827800	2.772787	H	3.338204	-2.524619	1.840568
C	3.233721	6.043267	-0.053061	H	5.738155	-3.116811	4.109841
H	3.594325	6.458288	-1.000890	H	4.951752	-1.892735	3.096392
H	2.154080	6.222653	-0.033273	H	7.473023	-3.468438	2.348709
C	-1.234368	5.718885	4.623471	H	7.447547	-1.808662	2.965308
H	-1.200370	5.193920	5.584738	H	2.659501	-4.875954	3.687772
H	-1.189468	6.793665	4.825547	H	3.133925	-3.280377	4.289858
H	-2.203141	5.506215	4.161404	H	7.364903	-1.813907	-3.160482
C	3.891116	6.771615	1.118428	H	7.408283	-3.472684	-2.542043
H	3.697112	7.847581	1.069447	H	5.630688	-3.128202	-4.260505
H	3.510061	6.406263	2.077315	H	3.289113	-2.528955	-1.929941
H	4.976838	6.624137	1.113664	H	3.086557	-4.231469	-1.460158
C	-1.383867	5.702825	-4.590878	H	2.559910	-4.880406	-3.757631
H	-1.356981	6.779001	-4.788579	H	3.017330	-3.284465	-4.371964
H	-1.371347	5.182894	-5.555435	H	-2.291916	-5.038484	-3.687151
H	-2.337201	5.476685	-4.103382	H	-2.870560	-3.475698	-4.284303
C	6.970431	-2.876834	-0.091555	C	-3.183355	-3.781085	-2.165711
C	0.117408	-4.228417	-3.244787	H	-2.792768	-4.425193	-1.374201
C	-6.721301	-3.320913	0.096560	H	-3.116860	-2.738913	-1.833868
C	0.203062	-4.223157	3.248278	C	-5.308635	-3.172893	-3.068669
O	-7.048123	-4.511488	0.103016	H	-4.773012	-2.216927	-3.097719
O	0.239692	-5.448060	3.095005	H	-5.477042	-3.495922	-4.103311
O	7.376724	-4.042720	-0.095978	C	-6.656168	-2.997677	-2.386715
O	0.159725	-5.452965	-3.090176	H	-7.183752	-3.951455	-2.339619

H	-7.266612	-2.296010	-2.962871
O	-4.545434	-4.145738	-2.353646
C	2.545428	-0.060824	-0.037043
H	3.164019	-0.300438	0.831953
H	3.137102	-0.300741	-0.924455
H	2.360635	1.013786	-0.034511
H	0.172974	-4.072209	0.003928

***m*-xylene-3⊂OCav**

C	2.104298	3.926974	-1.655179
C	3.304132	3.518903	-1.065132
C	3.945353	2.405976	-1.623043
C	3.373983	1.676127	-2.662180
C	2.148906	2.085718	-3.181614
C	1.523359	3.256019	-2.735880
H	1.604415	4.805524	-1.263851
H	3.878372	0.810202	-3.072001
C	0.289921	3.786620	-3.447823
H	0.289609	3.325981	-4.436264
C	3.858799	4.193402	0.188042
H	4.929190	3.987791	0.215660
C	-1.006254	3.356758	-2.780465
C	-1.726706	2.254108	-3.254547
C	-1.571695	4.089208	-1.733987
C	-3.005825	1.961133	-2.783310
C	-2.844324	3.824024	-1.222101
H	-1.009035	4.917631	-1.320160
C	-3.565228	2.766432	-1.792961
H	-3.574944	1.150762	-3.222072
C	3.235263	3.499398	1.396276
C	2.006766	3.903541	1.925573
C	3.842737	2.377699	1.977580
C	1.366857	3.229705	2.971560
H	1.526289	4.782424	1.512140
C	3.217193	1.647673	2.984712
C	1.968689	2.058000	3.447752
H	3.697067	0.775794	3.411773
C	-3.396363	4.602930	-0.033063
H	-4.482458	4.516598	-0.064608
C	0.099200	3.774884	3.610232
C	-2.915105	3.866978	1.209695
C	-1.672162	4.131102	1.794188
C	-3.679545	2.825112	1.750174
C	-1.167095	3.384402	2.863352
H	-1.079256	4.952488	1.408871
C	-3.186282	2.018526	2.770963
C	-1.922614	2.287411	3.290800
H	-3.787916	1.219520	3.184146
H	0.028824	3.301604	4.590190
O	5.224898	2.078572	-1.162675
O	5.143645	2.046038	1.583916
O	1.582944	1.375263	-4.247877
O	-1.206284	1.511702	-4.323290
O	-4.912171	2.610388	-1.439776
O	-4.997974	2.672403	1.305985
O	-1.434228	1.499227	4.340035
O	1.346997	1.322401	4.469732
C	5.418101	0.871599	-0.499797
C	5.374017	0.853838	0.904784
C	5.784038	-0.263431	-1.228996
C	5.699966	-0.300024	1.624508
C	6.091228	-1.411047	-0.511586
H	5.850378	-0.220925	-2.308777
C	6.053866	-1.428432	0.896718
H	5.702323	-0.285736	2.707113
C	0.597800	0.211939	4.065040
C	1.248392	-1.008704	3.837467
C	-0.801296	0.310921	3.972750
C	0.464584	-2.099981	3.488617
H	2.324293	-1.081565	3.937384
C	-1.584074	-0.789301	3.605377
C	-0.931238	-1.983035	3.350982
H	-2.661607	-0.705581	3.548226
C	0.825159	0.238160	-3.957397
C	1.478045	-0.982320	-3.743653
C	-0.578588	0.302873	-4.020362
C	0.693888	-2.120513	-3.620866
H	2.559599	-1.026398	-3.713150
C	-1.364409	-0.850419	-3.904814
C	-0.710147	-2.058306	-3.717056
H	-2.441764	-0.786256	-3.994482
C	-5.315125	1.468853	-0.753964
C	-5.758489	0.351023	-1.466840
C	-5.356120	1.499567	0.650310
C	-6.181085	-0.747727	-0.730094
H	-5.737042	0.349535	-2.549411
C	-5.829064	0.410446	1.387161
C	-6.209115	-0.719460	0.676986
H	-5.870654	0.456442	2.467873
N	-6.636023	-1.972006	1.104810
N	-6.604296	-2.014971	-1.119577
N	-1.181810	-3.367200	-3.653818
N	6.522278	-2.667629	-0.923890
N	0.782834	-3.429115	3.220852
N	1.031539	-3.462741	-3.470487
N	6.473591	-2.692889	1.298542
N	-1.418204	-3.233078	2.981944
C	-3.053706	6.101037	-0.044232
H	-3.450475	6.538002	0.878965
H	-1.970816	6.257981	-0.013629

C	0.170698	5.296973	3.855218	H	6.961668	-4.188897	2.615424
H	0.255147	5.838375	2.907459	H	4.575842	-2.379105	3.276398
H	-0.782705	5.604246	4.299164	H	5.099681	-3.796868	4.203164
C	1.321438	5.693423	4.778935	H	2.968385	-2.676517	1.869663
H	1.304427	6.768787	4.982028	H	2.579070	-3.647373	4.219698
H	1.252950	5.168462	5.738361	H	1.961008	-5.110581	3.440369
H	2.292425	5.452464	4.335675	O	4.460143	-4.190819	-2.212931
C	3.685112	5.721399	0.199280	O	4.305580	-4.200142	2.312948
H	2.626265	5.996833	0.158325	H	4.761344	-2.287849	-2.990246
H	4.059264	6.091892	1.160202	H	5.393831	-3.608934	-3.989856
C	0.346283	5.313380	-3.666021	H	3.009258	-2.768600	-1.804554
H	-0.586503	5.611923	-4.157369	H	2.642250	-4.447521	-1.353892
H	0.369559	5.841578	-2.707334	H	2.296837	-5.077386	-3.693872
C	4.431312	6.402479	-0.947206	H	2.941641	-3.530960	-4.262968
H	5.499430	6.159695	-0.920306	H	7.112050	-4.114379	-2.257307
H	4.042530	6.085171	-1.920133	H	7.242552	-2.459490	-2.874653
H	4.331701	7.490676	-0.886683	H	-3.045984	-3.169553	-4.534442
C	1.539334	5.743686	-4.518279	H	-2.571353	-4.826722	-4.129811
H	1.518310	6.822659	-4.700716	H	-3.054793	-2.681234	-2.016445
H	2.488764	5.506288	-4.028291	H	-5.540672	-2.949345	-4.219445
H	1.530399	5.237507	-5.490125	H	-4.749681	-1.827100	-3.098475
C	-3.639295	6.824871	-1.255865	H	-7.277368	-3.434098	-2.451456
H	-4.726718	6.698386	-1.301135	H	-7.281517	-1.777582	-3.074441
H	-3.426613	7.897492	-1.211450	H	-7.489151	-3.268607	2.466914
H	-3.220284	6.439202	-2.190566	H	-7.523630	-1.562536	2.934733
C	-0.120025	-4.238203	-3.460629	C	-5.662747	-2.533620	3.341158
C	-6.856409	-2.792285	0.005338	H	-4.944521	-1.726909	3.148125
C	-0.360487	-4.119422	2.843185	H	-5.953610	-2.475416	4.399145
C	6.698088	-3.487213	0.181997	C	-3.750635	-3.914145	3.593790
O	-0.425337	-5.294621	2.469345	H	-3.631910	-3.290057	4.487773
O	7.027755	-4.676904	0.175532	H	-3.590578	-4.956822	3.881859
O	-0.185333	-5.465389	-3.336827	C	-2.765173	-3.494347	2.503750
O	-7.234229	-3.967168	0.024030	H	-3.131461	-2.581359	2.036734
C	-2.568474	-3.794825	-3.775528	H	-2.708267	-4.266940	1.736020
C	-3.289071	-3.661495	-2.433579	O	-5.077444	-3.807691	3.071751
H	-2.930667	-4.429392	-1.744184	C	1.196880	-1.597017	-0.051013
C	-5.364705	-2.732938	-3.158063	C	0.615114	-0.325902	-0.002408
C	-6.710027	-2.502645	-2.487397	C	-0.776785	-0.219186	-0.029232
C	-6.905849	-2.346331	2.486696	C	-1.592920	-1.350376	-0.086579
O	-4.698767	-3.825535	-2.524061	C	-0.992335	-2.614566	-0.131944
C	2.098760	-4.035033	3.317149	C	0.395202	-2.736881	-0.117190
C	2.973610	-3.749270	2.095174	H	0.842875	-3.723842	-0.145081
H	2.598102	-4.288765	1.222839	H	-1.609651	-3.503931	-0.162967
C	5.052031	-3.363436	3.196705	H	2.278088	-1.684966	-0.028955
C	6.464294	-3.218893	2.653711	C	-3.079906	-1.162218	-0.096895
C	2.376117	-4.014415	-3.461266	H	-3.390559	-0.655795	-1.013726
C	3.090680	-3.814065	-2.122578	H	-3.625236	-2.103970	-0.029580
C	5.254537	-3.266600	-2.956947	H	-3.390327	-0.524253	0.733646
C	6.616117	-3.143563	-2.294679	C	1.445316	0.914265	0.099416
H	7.043625	-2.559898	3.306771	H	1.236088	1.588331	-0.731889

H	1.205917	1.456180	1.015275
H	2.510534	0.686666	0.101950
H	-1.237217	0.767070	0.012208

***m*-xylene-4⊂OCav**

C	2.601008	-4.028428	0.502032
C	3.319223	-3.617040	-0.622852
C	4.307712	-2.644919	-0.435569
C	4.551885	-2.089268	0.818364
C	3.799732	-2.516715	1.910510
C	2.831073	-3.517758	1.781314
H	1.819872	-4.769798	0.374385
H	5.314451	-1.329635	0.941076
C	2.034109	-4.020924	2.974953
H	2.551810	-3.671371	3.868735
C	2.987111	-4.163857	-2.001222
H	3.811044	-3.889669	-2.660912
C	0.658331	-3.374552	2.986177
C	0.478713	-2.130450	3.603554
C	-0.465889	-3.991745	2.431034
C	-0.776477	-1.530683	3.686498
C	-1.748304	-3.445855	2.525842
H	-0.346283	-4.955182	1.951785
C	-1.876502	-2.196997	3.148893
H	-0.910688	-0.604788	4.231242
C	1.748336	-3.446674	-2.525318
C	0.465925	-3.992569	-2.430412
C	1.876521	-2.197955	-3.148643
C	-0.658296	-3.375476	-2.985668
H	0.346324	-4.955907	-1.950959
C	0.776508	-1.531767	-3.686410
C	-0.478672	-2.131509	-3.603311
H	0.910717	-0.606014	-4.231386
C	-2.987041	-4.163255	2.001959
H	-3.810986	-3.888902	2.661565
C	-2.034114	-4.021762	-2.974273
C	-3.319164	-3.616871	0.623426
C	-2.600979	-4.028565	-0.501358
C	-4.307652	-2.644800	0.435906
C	-2.831051	-3.518217	-1.780770
H	-1.819864	-4.769929	-0.373535
C	-4.551814	-2.089436	-0.818151
C	-3.799676	-2.517167	-1.910202
H	-5.314360	-1.329814	-0.941053
H	-2.551799	-3.672427	-3.868151
O	5.097385	-2.248589	-1.525257
O	3.168238	-1.751316	-3.420727
O	4.054852	-1.963206	3.174284
O	1.556604	-1.603816	4.319610
O	-3.168222	-1.750328	3.420972
O	-5.097327	-2.248279	1.525517
O	-4.054807	-1.963910	-3.174088
O	-1.556591	-1.604994	-4.319391
C	4.881024	-0.943295	-1.965149
C	3.798636	-0.670331	-2.822907
C	5.793287	0.048857	-1.598429
C	3.529849	0.636379	-3.241537
C	5.559210	1.331297	-2.074084
H	6.647452	-0.197521	-0.980081
C	4.409411	1.628627	-2.827859
H	2.699083	0.846319	-3.903482
C	-2.265151	-0.491163	-3.889296
C	-1.759515	0.789524	-4.132231
C	-3.562403	-0.672309	-3.374905
C	-2.589607	1.865051	-3.856889
H	-0.767769	0.921789	-4.545743
C	-4.403446	0.416545	-3.124500
C	-3.899323	1.683984	-3.375956
H	-5.405432	0.255815	-2.746113
C	3.562536	-0.671535	3.374845
C	4.403664	0.417193	3.124167
C	2.265334	-0.490174	3.889287
C	3.899715	1.684728	3.375466
H	5.405608	0.256282	2.745750
C	1.759875	0.790613	4.132081
C	2.590065	1.866006	3.856501
H	0.768169	0.923031	4.545644
C	-3.798786	-0.669588	2.822862
C	-3.530233	0.637241	3.241268
C	-4.881099	-0.942868	1.965108
C	-4.409908	1.629287	2.827327
H	-2.699518	0.847403	3.903209
C	-5.793476	0.049084	1.598128
C	-5.559618	1.331652	2.073535
H	-6.647577	-0.197554	0.979795
N	-6.285202	2.512385	1.951013
N	-4.444064	2.995368	3.097655
N	2.392461	3.237276	3.999497
N	6.284624	2.512161	-1.951807
N	-2.391897	3.236278	-4.000252
N	4.460524	2.952641	3.241194
N	4.443425	2.994642	-3.098590
N	-4.460046	2.951972	-3.242022
C	-2.885137	-5.699285	2.018990
H	-3.800948	-6.091778	1.563280
H	-2.061167	-6.043363	1.385342
C	-1.988569	-5.559388	-3.047320
H	-1.356451	-5.836693	-3.898567
H	-1.506752	-5.976754	-2.157301
C	-3.377793	-6.174599	-3.207903

H	-3.314729	-7.262846	-3.305249	H	-0.640616	3.354470	-5.113223
H	-4.012732	-5.952293	-2.344410	H	-1.336067	4.904078	-4.609533
H	-3.880095	-5.784751	-4.100253	O	6.811146	3.888899	0.540088
C	2.885287	-5.699900	-2.017784	O	1.059120	4.335550	-3.488089
H	3.801039	-6.092200	-1.561790	H	7.189738	1.838022	0.584963
H	2.061225	-6.043819	-1.384170	H	8.613182	2.891028	0.504823
C	1.988480	-5.558530	3.048424	H	5.114190	2.677218	0.617569
H	1.356227	-5.835567	3.899657	H	4.886514	4.403966	0.960066
H	1.506775	-5.976123	2.158451	H	6.125206	4.178419	3.094644
C	2.728541	-6.270670	-3.426878	H	6.421385	2.464239	2.779155
H	3.557936	-5.956491	-4.070278	H	8.043985	3.565394	-1.797955
H	2.716290	-7.364896	-3.404753	H	8.224726	1.805579	-1.683957
H	1.798571	-5.935625	-3.895971	H	0.642246	3.356236	5.114035
C	3.377651	-6.173765	3.209387	H	1.337070	4.905469	4.608313
H	3.314516	-7.261981	3.307036	H	0.134863	2.837247	2.687954
H	4.012727	-5.951741	2.345923	H	-1.907403	2.769821	2.441376
H	3.879844	-5.783687	4.101698	H	-2.364194	4.382503	1.857872
C	-2.728100	-6.269594	3.428237	H	-3.733948	4.762588	3.892500
H	-3.557392	-5.955253	4.071690	H	-3.071006	3.296219	4.635529
H	-2.715795	-7.363827	3.406459	H	-8.044619	3.565414	1.796472
H	-1.798059	-5.934349	3.897048	H	-8.225142	1.805572	1.682600
C	3.514871	3.918087	3.554194	C	-7.588095	2.757662	-0.144360
C	-5.582073	3.557892	2.533142	H	-7.189492	1.837781	-0.585946
C	-3.514259	3.917266	-3.555076	H	-8.612944	2.890828	-0.506443
C	5.581299	3.557455	-2.534075	C	-5.552212	3.549987	-1.113531
O	-3.655410	5.141820	-3.472552	H	-4.886131	4.403531	-0.961010
O	5.913782	4.745730	-2.572607	H	-5.113978	2.676820	-0.618440
O	3.656142	5.142612	3.471445	C	-5.723517	3.286212	-2.610098
O	-5.914749	4.746119	2.571479	H	-6.124553	4.178000	-3.095712
C	1.127092	3.883700	4.289074	H	-6.421026	2.463880	-2.780161
C	0.213887	3.863127	3.067827	O	-6.810844	3.888645	-0.541205
H	0.627517	4.490129	2.265710	C	-0.000038	-1.248605	0.000024
C	-2.148245	3.790559	2.758015	C	-1.201534	-0.553857	-0.140221
C	-3.359656	3.759087	3.687550	C	-1.215481	0.847583	-0.146131
C	-7.612448	2.657344	1.372297	C	0.000063	1.530915	-0.000037
O	-1.059552	4.334224	3.490434	C	1.215566	0.847497	0.146052
C	-1.126309	3.882601	-4.289200	C	1.201516	-0.553935	0.140215
C	-0.214404	3.863260	-3.066956	H	-0.000075	-2.333005	0.000029
H	-0.629589	4.490139	-2.265559	H	2.133485	-1.095196	0.246017
C	2.147991	3.790017	-2.757299	H	0.000104	2.618979	-0.000075
C	3.358590	3.758257	-3.687866	H	-2.133535	-1.095058	-0.246073
C	5.723979	3.286664	2.609125	C	2.510813	1.603960	0.290970
C	5.552544	3.550378	1.112562	H	2.420306	2.413590	1.021523
C	7.588209	2.757832	0.143108	H	3.318959	0.942485	0.606612
C	7.612048	2.657323	-1.373541	H	2.810338	2.062488	-0.654390
H	3.069213	3.295211	-4.635529	C	-2.510694	1.604080	-0.291179
H	3.732745	4.761716	-3.893289	H	-2.810928	2.061693	0.654408
H	2.365413	4.380894	-1.856812	H	-2.419843	2.414358	-1.020981
H	1.906472	2.769202	-2.441464	H	-3.318572	0.942782	-0.607877
H	-0.134929	2.837577	-2.686710				

***m*-xylene-5⊂OCav**

C	-0.956209	-2.113361	-0.439470
C	0.282795	-2.687238	-0.150687
C	1.363625	-1.886052	0.234647
C	1.170255	-0.500221	0.316871
C	-0.064483	0.089019	0.021939
C	-1.134834	-0.735142	-0.351311
H	-1.790174	-2.748282	-0.710398
H	-2.103200	-0.291302	-0.570043
H	2.001031	0.134877	0.617802
H	0.406582	-3.764399	-0.215600
C	-0.233310	1.579682	0.058147
H	0.441317	2.043903	0.778540
H	-0.011579	2.007473	-0.923000
H	-1.258449	1.855844	0.308683
C	2.707422	-2.490631	0.542701
H	3.363397	-2.470048	-0.335117
H	3.214637	-1.940909	1.337107
H	2.609950	-3.533664	0.855210
C	1.696325	4.008504	2.039521
C	1.286219	3.222800	3.122137
C	2.079806	2.117726	3.449537
C	3.256063	1.840546	2.759723
C	3.644464	2.666556	1.710203
C	2.867513	3.766124	1.316854
H	1.072186	4.844656	1.747684
H	3.880556	1.010126	3.063744
C	3.257234	4.632413	0.123565
H	4.335176	4.529091	-0.006665
C	0.031482	3.561092	3.911035
H	0.117511	3.042644	4.866820
C	2.602294	4.045163	-1.122431
C	3.267410	3.059790	-1.861241
C	1.320545	4.413940	-1.547927
C	2.654065	2.408309	-2.923304
C	0.684776	3.815106	-2.641750
H	0.801218	5.200620	-1.012421
C	1.357974	2.764498	-3.273687
H	3.184330	1.657520	-3.492271
C	-1.209420	2.997600	3.230197
C	-1.958195	3.727220	2.301391
C	-1.659857	1.712629	3.550807
C	-3.155790	3.255150	1.756279
H	-1.606292	4.709935	2.010961
C	-2.853243	1.206747	3.044142
C	-3.599196	1.992134	2.172558
H	-3.225922	0.246772	3.376403
C	-0.660615	4.282989	-3.181752
H	-0.691469	3.968424	-4.225881
C	-3.941417	4.031899	0.702939

C	-1.835717	3.600473	-2.497396
C	-2.343600	4.076661	-1.283784
C	-2.476656	2.493613	-3.069124
C	-3.476853	3.542657	-0.664292
H	-1.826348	4.895209	-0.797220
C	-3.625805	1.950190	-2.500974
C	-4.125734	2.487774	-1.319548
H	-4.145961	1.136949	-2.991197
H	-4.987945	3.743931	0.808953
O	1.747622	1.318138	4.552498
O	-0.974012	0.991930	4.530277
O	4.892718	2.459926	1.113561
O	4.607636	2.790634	-1.570178
O	0.741710	2.104395	-4.340756
O	-2.005722	1.945094	-4.274918
O	-5.352353	2.027395	-0.827758
O	-4.890191	1.602528	1.817091
C	1.220114	0.060457	4.257727
C	-0.173831	-0.087229	4.171898
C	2.079112	-1.039642	4.148044
C	-0.751585	-1.330607	3.901848
C	1.499110	-2.281430	3.929337
H	3.148904	-0.913310	4.259327
C	0.106268	-2.412532	3.769835
H	-1.826660	-1.444923	3.853643
C	-5.131228	0.496538	1.013935
C	-5.272662	-0.771919	1.582640
C	-5.418181	0.724620	-0.342518
C	-5.727209	-1.792050	0.757791
H	-5.070042	-0.931166	2.633604
C	-5.894571	-0.298446	-1.166535
C	-6.058913	-1.552427	-0.591044
H	-6.153737	-0.095496	-2.198230
C	5.070536	1.350248	0.292210
C	5.511414	0.135980	0.830667
C	4.933080	1.525768	-1.093746
C	5.806044	-0.887021	-0.061814
H	5.649253	0.025875	1.899051
C	5.230996	0.497155	-1.989952
C	5.660133	-0.706943	-1.452150
H	5.166556	0.658806	-3.058274
C	0.148801	0.875308	-4.050597
C	0.941076	-0.248504	-3.792928
C	-1.250166	0.779539	-4.108595
C	0.289608	-1.461385	-3.637884
H	2.018799	-0.172191	-3.736919
C	-1.896650	-0.459654	-4.021826
C	-1.103775	-1.576424	-3.802579
H	-2.973732	-0.526672	-4.108822
N	-1.412758	-2.927808	-3.676011

N	0.775165	-2.733548	-3.362872
N	6.085165	-1.879090	-2.068703
N	2.038797	-3.565485	3.854591
N	-6.034879	-3.124246	1.020348
N	6.315191	-2.170032	0.125875
N	-0.163747	-3.765416	3.592236
N	-6.580100	-2.738821	-1.099714
C	-0.799957	5.819228	-3.172353
H	-1.804145	6.063034	-3.536291
H	-0.742604	6.206775	-2.150196
C	-3.870618	5.559754	0.861169
H	-2.838947	5.916486	0.777286
H	-4.413674	6.004218	0.019737
C	-4.474352	6.043388	2.179234
H	-5.515364	5.715790	2.276941
H	-3.921482	5.654813	3.040215
H	-4.458571	7.136155	2.238111
C	-0.076510	5.066558	4.222997
H	0.876565	5.381775	4.662036
H	-0.194025	5.647692	3.302567
C	2.964756	6.127751	0.333864
H	1.890730	6.304603	0.450953
H	3.427887	6.427717	1.280411
C	-1.218948	5.390380	5.184940
H	-1.108171	4.838268	6.124964
H	-1.237187	6.458632	5.422505
H	-2.191188	5.126857	4.757266
C	3.502067	6.994174	-0.804588
H	4.580581	6.849574	-0.932882
H	3.018293	6.749650	-1.755602
H	3.326087	8.055575	-0.604000
C	0.246834	6.512738	-4.043366
H	1.261064	6.329775	-3.675079
H	0.197811	6.152667	-5.077154
H	0.086561	7.595357	-4.056350
C	6.502687	-2.783374	-1.106434
C	-0.268528	-3.642448	-3.352346
C	-6.517003	-3.728987	-0.129993
C	1.015822	-4.487040	3.654684
O	-6.853853	-4.911651	-0.250750
O	1.144900	-5.713590	3.582166
O	6.984079	-3.904873	-1.297679
O	-0.193558	-4.853460	-3.114963
C	6.240267	-2.085130	-3.500983
C	4.923102	-2.253568	-4.231644
H	4.242541	-1.431544	-3.977500
C	2.956437	-3.564205	-4.186592
C	2.140429	-3.058905	-2.993942
C	-2.717141	-3.537287	-3.857249
O	4.348231	-3.511546	-3.881329

C	-5.814981	-3.857565	2.258878
C	-4.430337	-4.475085	2.327962
H	-4.339943	-5.063068	3.251892
C	-2.138046	-3.831226	2.111294
C	-1.490158	-4.335632	3.413826
C	6.881850	-2.743513	1.341269
C	6.096796	-3.906776	1.916789
C	4.306672	-4.514328	3.297591
C	3.340927	-4.003295	4.350119
H	-2.116834	-4.034620	4.260131
H	-1.376344	-5.419309	3.437959
H	-2.073523	-4.589421	1.320832
H	-1.606357	-2.945901	1.769742
H	-4.276031	-5.148932	1.473754
H	-5.968653	-3.171520	3.094796
H	-6.568513	-4.645143	2.318861
O	4.992687	-3.429883	2.679269
O	-3.483578	-3.414654	2.290852
H	5.035591	-5.164881	3.801601
H	3.779140	-5.120610	2.549146
H	5.756599	-4.572558	1.112660
H	6.768829	-4.484632	2.567836
H	7.888544	-3.095981	1.100269
H	6.973470	-1.949609	2.084504
H	3.159114	-4.812865	5.062396
H	3.794082	-3.173280	4.895604
H	6.774591	-1.227722	-3.921335
H	6.860968	-2.973402	-3.631007
H	5.108711	-2.211647	-5.313872
H	2.704575	-4.606263	-4.401619
H	2.731469	-2.968225	-5.079802
H	2.594801	-2.156518	-2.580528
H	2.115559	-3.810313	-2.203281
H	-2.568170	-4.543325	-4.254828
H	-3.255078	-2.952526	-4.607446
C	-3.532384	-3.610248	-2.565646
H	-3.068482	-4.311655	-1.866965
H	-3.585975	-2.624709	-2.086760
C	-5.725495	-3.120772	-3.379464
H	-5.222865	-2.150539	-3.452143
H	-6.045852	-3.411774	-4.386218
C	-6.947672	-3.012267	-2.478686
H	-7.500165	-3.952984	-2.472593
H	-7.612088	-2.223485	-2.842735
O	-4.836801	-4.105896	-2.846653

***p*-xylene⊂MCav**

Pd	5.075912	-2.940044	-0.091430
Pd	-5.040480	-2.978345	-0.070312
C	-0.029828	3.990977	-2.396338

C	1.202210	3.492965	-2.831846	C	4.006032	-0.484935	-1.935569
C	1.169916	2.423032	-3.734773	C	2.528204	-1.743924	-3.439761
C	-0.032077	1.903053	-4.200231	H	1.498242	-0.567535	-4.924356
C	-1.233016	2.424012	-3.732349	C	3.409603	-1.701951	-2.316552
C	-1.263210	3.491413	-2.826427	H	4.724199	-0.477707	-1.128272
H	-0.028990	4.816194	-1.694465	C	3.715765	0.472588	2.729328
H	-0.032594	1.112196	-4.937398	C	4.049074	-0.631647	1.980660
C	-2.570578	4.110123	-2.347389	C	2.752855	0.375256	3.775024
H	-3.328157	3.833528	-3.082234	C	3.430070	-1.868535	2.244535
C	2.511335	4.115682	-2.364078	H	4.779945	-0.570447	1.187354
H	3.263483	3.839792	-3.104703	C	2.210633	-0.844173	4.107376
C	-3.013751	3.500299	-1.023316	C	2.533320	-1.996763	3.349335
C	-3.892581	2.412887	-1.027329	H	1.505460	-0.940291	4.922844
C	-2.603213	3.983710	0.226676	C	-2.774014	0.653622	-3.657971
C	-4.383575	1.860591	0.144633	C	-2.247463	-0.532491	-4.112806
C	-3.013834	3.405261	1.437348	C	-3.710560	0.658660	-2.584007
H	-1.921794	4.826041	0.259560	C	-2.560028	-1.747302	-3.454922
C	-3.893976	2.322407	1.357483	H	-1.565735	-0.557665	-4.953425
H	-5.138166	1.089682	0.114681	C	-4.024192	-0.505398	-1.922697
C	2.968189	3.510618	-1.042918	C	-3.421852	-1.715605	-2.316157
C	2.560241	3.991385	0.208819	H	-4.730260	-0.507067	-1.104807
C	3.860652	2.434468	-1.051407	C	-3.697462	0.442166	2.747033
C	2.989506	3.422493	1.417283	C	-4.023024	-0.663502	1.997151
H	1.869093	4.825679	0.244896	C	-2.727419	0.351842	3.786643
C	4.368309	1.891561	0.117941	C	-3.386630	-1.893499	2.251634
C	3.882376	2.350526	1.333264	H	-4.760649	-0.609315	1.209566
H	5.132155	1.129867	0.083875	C	-2.168592	-0.862127	4.111028
C	-2.555771	3.912367	2.801155	C	-2.480341	-2.015032	3.349219
H	-3.301901	3.568999	3.519474	H	-1.457541	-0.952863	4.922011
C	2.540634	3.931430	2.783325	N	-1.897681	-3.204443	3.661407
C	-1.236563	3.272778	3.215975	N	-3.626451	-2.987059	1.460397
C	-0.006789	3.821132	2.834589	N	-3.657836	-2.876808	-1.626439
C	-1.196172	2.121432	4.012783	N	1.987308	-2.914818	-3.876001
C	1.229853	3.283380	3.209406	N	3.678949	-2.962670	1.456875
H	-0.011981	4.703678	2.207232	N	-2.015732	-2.912103	-3.902793
C	0.008023	1.564920	4.425873	N	3.672213	-2.860638	-1.631460
C	1.204964	2.131785	4.006326	N	1.969730	-3.192678	3.671916
H	0.013603	0.711718	5.089748	C	-2.523172	5.450953	2.889885
H	3.295289	3.596031	3.496414	H	-2.147476	5.717259	3.883925
O	2.366816	1.870047	-4.228803	H	-1.810981	5.868965	2.171165
O	4.260487	1.884834	-2.279027	C	2.497438	5.470089	2.866410
O	-2.430890	1.871213	-4.224913	H	1.775140	5.880011	2.153204
O	-4.298601	1.864732	-2.253388	H	2.129565	5.737446	3.863114
O	-4.297547	1.669790	2.533611	C	3.866439	6.107400	2.629926
O	-2.389179	1.518042	4.453911	H	3.815174	7.194055	2.749953
O	2.406062	1.539789	4.440903	H	4.606317	5.724029	3.341495
O	4.302131	1.705594	2.508110	H	4.235242	5.899579	1.620623
C	2.721284	0.658129	-3.656452	C	2.466078	5.655845	-2.321287
C	3.674242	0.674049	-2.597171	H	1.720614	6.005586	-1.600223
C	2.194107	-0.533762	-4.095712	H	3.436599	6.002390	-1.949252

C	-2.529223	5.650193	-2.301504
H	-3.498549	5.993167	-1.923074
H	-1.780571	6.000428	-1.583982
C	2.185897	6.273804	-3.690874
H	2.216294	7.366535	-3.637936
H	2.930830	5.950638	-4.426683
H	1.199315	5.984993	-4.066814
C	-2.258203	6.272357	-3.670969
H	-2.295511	7.364763	-3.615836
H	-1.271005	5.990754	-4.050637
H	-3.003594	5.945716	-4.404778
C	-3.899190	6.078892	2.669518
H	-3.854786	7.165454	2.793062
H	-4.276708	5.872023	1.663250
H	-4.628960	5.687438	3.387101
C	-2.264863	-3.997814	-3.198102
C	-3.008765	-4.122505	1.742134
C	2.234840	-4.212291	2.878775
C	3.099272	-3.981350	-2.035840
N	6.633850	-3.052370	1.271959
H	6.681322	-4.012392	1.617180
N	6.613451	-2.958628	-1.482169
H	6.411179	-3.574792	-2.269268
C	7.874727	-3.358509	-0.793047
C	7.895678	-2.689015	0.564149
H	6.690663	-2.009021	-1.851353
H	6.476501	-2.444697	2.076328
C	3.081530	-4.105957	1.750969
C	2.263317	-3.997697	-3.177229
H	3.304082	-4.886783	-1.477743
H	1.831707	-4.939728	-3.498981
H	3.275304	-4.959039	1.111996
H	1.781618	-5.171298	3.107954
C	-3.076260	-3.990284	-2.039130
H	-1.829342	-4.934989	-3.528730
H	-3.256137	-4.896977	-1.474351
C	-2.152435	-4.222791	2.863270
H	-3.193438	-4.974164	1.098564
H	-1.683152	-5.176204	3.083325
N	-6.580166	-3.122882	1.310725
H	-6.603633	-4.084222	1.654795
N	-6.593375	-3.013297	-1.443010
H	-6.388669	-3.618288	-2.238095
C	-7.838073	-3.444216	-0.742514
C	-7.858038	-2.784374	0.619390
H	-6.425417	-2.513485	2.114253
H	-6.693283	-2.061924	-1.802030
H	-7.799417	-4.531098	-0.642148
H	-8.720417	-3.173511	-1.327659
H	-8.713916	-3.120082	1.210306
H	-7.900353	-1.697468	0.520292
H	8.744451	-3.073173	-1.389989
H	7.860429	-4.445219	-0.684885
H	7.911378	-1.602152	0.457250
H	8.765880	-3.000729	1.147246
C	0.031407	-2.280230	1.019243
C	0.016258	-0.898383	1.186489
C	-0.014591	-0.038349	0.077775
C	-0.029235	-0.611223	-1.196537
C	-0.013150	-1.999924	-1.358756
C	0.018156	-2.859587	-0.257795
C	-0.042715	1.450075	0.276367
H	0.790049	1.776492	0.904350
H	0.014717	1.983643	-0.673204
H	-0.962417	1.756430	0.782290
H	0.027230	-0.476381	2.187036
H	-0.023207	-2.417377	-2.355567
H	0.053762	-2.922457	1.888898
H	-0.053307	0.030879	-2.071781
C	0.047936	-4.358402	-0.421222
H	0.987826	-4.779266	-0.046524
H	-0.760304	-4.836606	0.142608
H	-0.051981	-4.647684	-1.469956

***p*-xylene-1⊂MCav**

Pd	-5.621628	-3.003407	-0.086385
Pd	5.792636	-2.736756	-0.086117
C	-0.076834	3.809536	2.723108
C	-1.297286	3.212618	3.062373
C	-1.237368	2.005969	3.770870
C	-0.026879	1.434681	4.142064
C	1.158112	2.049734	3.761387
C	1.166102	3.258504	3.055323
H	-0.095393	4.741857	2.172730
H	-0.007712	0.549343	4.760468
C	2.480665	3.939899	2.699329
H	3.207360	3.618404	3.447364
C	-2.638266	3.837350	2.695547
H	-3.354147	3.484779	3.440223
C	3.002603	3.445780	1.355065
C	3.967459	2.436430	1.283328
C	2.549434	3.977669	0.140049
C	4.482174	2.005871	0.069846
C	3.001389	3.533784	-1.109993
H	1.790575	4.751503	0.168100
C	3.967587	2.522355	-1.109329
H	5.271600	1.270626	0.039763
C	-3.135521	3.318882	1.349716
C	-2.715304	3.872454	0.133319
C	-4.046269	2.259713	1.279307

C	-3.141117	3.402454	-1.116248	C	4.453903	-0.404271	-1.975778
H	-2.003018	4.689314	0.158052	C	2.808078	0.613551	-3.456974
C	-4.537024	1.800617	0.067000	C	3.864010	-1.663779	-2.207480
C	-4.051527	2.341355	-1.113737	H	5.304514	-0.327497	-1.311192
H	-5.285766	1.024284	0.038508	C	2.246024	-0.611987	-3.723822
C	2.481383	4.122170	-2.415102	C	2.749431	-1.775974	-3.096631
H	3.205320	3.850730	-3.185295	H	1.414408	-0.717871	-4.407455
C	-2.645911	4.013184	-2.423280	N	2.152591	-2.973024	-3.350291
C	1.162582	3.472129	-2.820547	N	4.318673	-2.790491	-1.570755
C	-0.081408	3.997646	-2.449250	N	4.359995	-2.906185	1.427110
C	1.155036	2.317188	-3.610967	N	-2.049134	-3.310604	3.243312
C	-1.300600	3.421800	-2.828319	N	-4.139898	-2.989403	-1.562525
H	-0.100565	4.892234	-1.838750	N	2.235809	-3.225602	3.237025
C	-0.028719	1.728470	-4.034541	N	-4.173048	-3.092732	1.419373
C	-1.238639	2.265990	-3.617019	N	-1.970541	-3.067055	-3.345148
H	-0.008891	0.888157	-4.712981	C	2.422189	5.661432	-2.373216
H	-3.359212	3.711496	-3.192262	H	1.702791	6.003449	-1.622303
O	-2.427352	1.390582	4.188428	H	3.402888	6.017165	-2.038665
O	-4.480993	1.613669	2.451243	C	-2.639424	5.553703	-2.390427
O	2.374938	1.488744	4.178755	H	-1.963377	5.923673	-1.613020
O	4.432815	1.810606	2.454798	H	-2.232658	5.905756	-3.344940
O	4.433190	1.978159	-2.320999	C	-4.034709	6.138427	-2.172742
O	2.372224	1.779315	-4.055928	H	-4.730386	5.793679	-2.945995
O	-2.428447	1.668032	-4.058110	H	-4.443879	5.844163	-1.200917
O	-4.490183	1.768739	-2.322476	H	-4.007858	7.231982	-2.206661
C	-2.815157	0.245859	3.519908	C	-2.631108	5.376170	2.775213
C	-3.918206	0.359625	2.624777	H	-2.228777	5.656658	3.754868
C	-2.217068	-0.973359	3.731913	H	-1.951618	5.802989	2.030293
C	-4.398340	-0.741199	1.954344	C	2.402829	5.476770	2.777534
C	-2.670533	-2.117968	3.034092	H	1.710213	5.871548	2.027401
H	-1.396471	-1.088072	4.427394	H	1.980587	5.738963	3.753912
C	-3.767150	-1.990337	2.125338	C	-4.025264	5.976023	2.593876
H	-5.244125	-0.662769	1.284382	H	-4.725688	5.574484	3.334763
C	-3.915788	0.531346	-2.567862	H	-3.998543	7.063901	2.710212
C	-4.391463	-0.611777	-1.967875	H	-4.428681	5.756103	1.600209
C	-2.800124	0.483477	-3.452491	C	3.769393	6.139487	2.606715
C	-3.737989	-1.840941	-2.195485	H	4.481260	5.770956	3.353787
H	-5.246865	-0.576975	-1.306018	H	4.190040	5.937312	1.616629
C	-2.174554	-0.712182	-3.713199	H	3.692040	7.225128	2.721622
C	-2.619584	-1.899217	-3.084768	C	2.085133	6.277855	-3.730683
H	-1.338475	-0.777215	-4.396301	H	2.112007	7.370752	-3.678196
C	2.824914	0.364755	3.514362	H	2.803333	5.959193	-4.494463
C	2.286951	-0.882514	3.725070	H	1.086795	5.983892	-4.068804
C	3.929148	0.531030	2.628388	C	2.731034	-4.235008	2.549863
C	2.802686	-2.004559	3.034095	C	3.727060	-3.946291	-1.826345
H	1.466217	-1.035452	4.413159	C	-2.410807	-4.144646	-2.727339
C	4.468452	-0.546532	1.964554	C	-3.555508	-4.245058	1.628492
C	3.899100	-1.825092	2.134038	N	-7.161743	-2.948446	-1.474509
H	5.312612	-0.424891	1.298672	H	-7.003190	-3.591241	-2.249984
C	3.920728	0.714960	-2.572518	N	-7.180411	-3.024989	1.277408

H	-7.292935	-3.981498	1.617058
C	-8.415592	-2.573327	0.573764
C	-8.447491	-3.249294	-0.779712
H	-6.978233	-2.433776	2.083856
H	-7.175915	-2.003297	-1.862340
C	-3.496688	-4.116438	-1.822640
C	-2.492501	-4.343773	2.555243
H	-3.882892	-5.107699	1.060499
H	-2.014254	-5.305822	2.707060
H	-3.818960	-5.014582	-1.310077
H	-1.911093	-5.088203	-2.920716
C	3.793994	-4.085489	1.629498
H	2.294722	-5.217899	2.693430
H	4.162420	-4.930323	1.059976
C	2.640561	-4.026530	-2.727505
H	4.090604	-4.827040	-1.311336
H	2.178960	-4.991309	-2.910207
N	7.327934	-2.611518	-1.475927
H	7.191498	-3.253358	-2.256327
N	7.352772	-2.702373	1.276546
H	7.499467	-3.654938	1.614126
C	8.570618	-2.204917	0.573212
C	8.624204	-2.871269	-0.784287
H	7.307525	-1.663194	-1.855760
H	7.130861	-2.120111	2.084255
H	9.467444	-2.423166	1.158478
H	8.470485	-1.122348	0.467934
H	8.729637	-3.953166	-0.677028
H	9.457893	-2.491826	-1.380162
H	-8.352347	-1.488704	0.461869
H	-9.303665	-2.818672	1.161621
H	-9.294673	-2.903169	-1.376789
H	-8.514742	-4.333544	-0.665659
C	-1.046322	-2.195353	0.006493
C	-1.162782	-0.809226	0.022801
C	-0.021193	0.007487	0.027297
C	1.231684	-0.610171	0.016779
C	1.338729	-2.002648	0.000827
C	0.207758	-2.821146	-0.005096
C	-0.163167	1.499986	0.038256
H	-0.705811	1.831553	0.925442
H	0.807294	1.997199	0.026674
H	-0.732597	1.839630	-0.829010
H	-2.147529	-0.349852	0.030002
H	2.316710	-2.460383	-0.009661
H	-1.936017	-2.811513	-0.000866
H	2.131866	-0.001488	0.018172
C	0.319186	-4.323887	-0.016540
H	1.358873	-4.645065	-0.116673
H	-0.078445	-4.756749	0.908905
H	-0.253147	-4.756615	-0.844699

***o*-xylene⊂MCav**

Pd	5.947921	-2.788519	0.016480
Pd	-5.946796	-2.790528	-0.054510
C	0.035103	3.842784	-2.502543
C	1.272155	3.297860	-2.864234
C	1.256980	2.116146	-3.615203
C	0.066641	1.512282	-4.007054
C	-1.139333	2.087048	-3.619711
C	-1.186952	3.273570	-2.877537
H	0.023421	4.761327	-1.929631
H	0.076486	0.643643	-4.650468
C	-2.523815	3.925805	-2.553187
H	-3.217214	3.613867	-3.335606
C	2.592354	3.964059	-2.502095
H	3.309011	3.671813	-3.270984
C	-3.085114	3.394704	-1.241961
C	-4.074879	2.408078	-1.221204
C	-2.638117	3.873053	-0.005498
C	-4.625655	1.952914	-0.031436
C	-3.122591	3.400026	1.218944
H	-1.860109	4.628360	0.005003
C	-4.113106	2.414802	1.172738
H	-5.419765	1.221287	-0.041488
C	3.122339	3.416074	-1.184349
C	2.637466	3.872638	0.046295
C	4.113262	2.430877	-1.150920
C	3.083706	3.377065	1.276179
H	1.859827	4.628437	0.045430
C	4.625076	1.952177	0.047091
C	4.073046	2.390282	1.242557
H	5.419462	1.220814	0.046366
C	-2.591876	3.929885	2.543742
H	-3.309040	3.628770	3.308733
C	2.522085	3.888999	2.594779
C	-1.272927	3.256585	2.896545
C	-0.035738	3.804734	2.541077
C	-1.258325	2.065144	3.631912
C	1.185991	3.231012	2.909434
H	-0.023519	4.730092	1.979351
C	-0.068121	1.454548	4.014128
C	1.138172	2.034666	3.635577
H	-0.078084	0.576522	4.644769
H	3.215753	3.566312	3.372578
O	2.477901	1.591486	-4.067538
O	4.585303	1.863462	-2.350843
O	-2.343866	1.530024	-4.076523
O	-4.502712	1.812667	-2.424373
O	-4.584834	1.832054	2.365255

O	-2.479271	1.537698	4.080945	H	4.528620	5.695101	3.294951
O	2.342265	1.473516	4.088518	H	4.300627	5.841517	1.546571
O	4.500104	1.779539	2.438067	H	3.787410	7.153401	2.615719
C	2.963208	0.452991	-3.456913	C	2.518801	5.503179	-2.512416
C	4.083475	0.591450	-2.583767	H	2.087477	5.810718	-3.471509
C	2.435828	-0.789159	-3.716009	H	1.836938	5.866192	-1.736972
C	4.639188	-0.510424	-1.974921	C	-2.468937	5.465809	-2.585851
C	2.971903	-1.935988	-3.086290	H	-1.813115	5.849051	-1.797675
H	1.604724	-0.915670	-4.397044	H	-2.015407	5.764046	-3.537578
C	4.075758	-1.784521	-2.189928	C	3.890390	6.151266	-2.326030
H	5.485415	-0.409636	-1.307403	H	4.319978	5.901467	-1.350468
C	3.973789	0.507550	2.611242	H	4.592924	5.814083	-3.096401
C	4.537574	-0.578866	1.983359	H	3.817651	7.241389	-2.389790
C	2.825728	0.352655	3.444459	C	-3.852922	6.099353	-2.446909
C	3.957253	-1.854254	2.137329	H	-4.530313	5.741114	-3.230209
H	5.410787	-0.461815	1.354756	H	-4.305816	5.860118	-1.479279
C	2.275955	-0.891627	3.640785	H	-3.791664	7.189139	-2.526646
C	2.822934	-2.022974	2.991586	C	-3.885716	6.121841	2.395705
H	1.421989	-1.031942	4.290515	H	-4.314244	5.887031	1.415964
C	-2.822961	0.401183	-3.443235	H	-4.590155	5.774919	3.159990
C	-2.265106	-0.838373	-3.646352	H	-3.811035	7.210795	2.475321
C	-3.973370	0.543724	-2.611099	C	-2.744141	-4.214893	-2.564515
C	-2.806045	-1.976859	-3.004988	C	-4.006223	-4.093307	1.743781
H	-1.408444	-0.969908	-4.294225	C	2.776746	-4.259485	2.540416
C	-4.533874	-0.550456	-1.993756	C	3.983171	-4.062851	-1.772938
C	-3.945032	-1.820949	-2.154297	N	7.449605	-2.738359	1.445722
H	-5.408203	-0.443522	-1.365126	H	7.291962	-3.416048	2.191106
C	-4.085713	0.556690	2.584838	N	7.538294	-2.677659	-1.306786
C	-4.643224	-0.538091	1.964910	H	7.686834	-3.608498	-1.699494
C	-2.968342	0.406549	3.459812	C	8.743991	-2.233167	-0.549009
C	-4.086621	-1.816476	2.172773	C	8.759348	-2.973931	0.770756
H	-5.487907	-0.428719	1.296741	H	7.337573	-2.045699	-2.082088
C	-2.448209	-0.839922	3.712384	H	7.425982	-1.808912	1.869512
C	-2.988312	-1.979934	3.073728	C	3.869684	-4.119460	1.654333
H	-1.620353	-0.974640	4.395845	C	2.921354	-4.184479	-2.697755
N	-2.440634	-3.200462	3.323623	H	4.359232	-4.926888	-1.238842
N	-4.554950	-2.914413	1.497727	H	2.494267	-5.164846	-2.882124
N	-4.427351	-2.916801	-1.485863	H	4.257470	-4.972336	1.110880
N	2.415222	-3.151656	-3.340017	H	2.340226	-5.242509	2.683976
N	4.444316	-2.943183	1.460291	C	-3.842413	-4.087576	-1.683332
N	-2.227924	-3.191224	-3.213109	H	-2.299094	-5.193724	-2.710463
N	4.541407	-2.888993	-1.524303	H	-4.226703	-4.946178	-1.146607
N	2.255167	-3.242929	3.195841	C	-2.950371	-4.226687	2.673771
C	-2.515572	5.468636	2.574846	H	-4.384232	-4.952021	1.202271
H	-2.084794	5.762197	3.538549	H	-2.530988	-5.210850	2.855603
H	-1.832182	5.841296	1.805385	N	-7.547172	-2.689207	1.258893
C	2.466122	5.428325	2.650290	H	-7.445564	-3.337445	2.039323
H	1.808896	5.823109	1.868977	N	-7.438577	-2.729933	-1.492583
H	2.013757	5.711914	3.607033	H	-7.559652	-3.675688	-1.858085
C	3.849389	6.065054	2.518725	C	-8.694354	-2.258295	-0.839742

C	-8.805776	-2.950012	0.501577
H	-7.550685	-1.744080	1.647201
H	-7.182043	-2.128230	-2.275297
H	-9.559719	-2.475053	-1.471023
H	-8.611543	-1.176935	-0.710192
H	-8.893301	-4.030891	0.370226
H	-9.671558	-2.590523	1.063150
H	8.652121	-1.157011	-0.386197
H	9.652175	-2.428863	-1.124584
H	9.582646	-2.636859	1.405322
H	8.856676	-4.049408	0.606269
C	0.697939	-3.403185	-0.040829
C	1.377113	-2.183388	-0.075483
C	0.701556	-0.960472	-0.033510
C	-0.705413	-0.963184	0.038638
C	-1.376515	-2.188613	0.080654
C	-0.692889	-3.405861	0.045223
C	1.457788	0.337866	-0.066388
H	2.532613	0.165207	-0.077918
H	1.198086	0.928021	-0.952470
H	1.222665	0.958906	0.805011
H	-1.247627	-4.338371	0.080012
H	2.456175	-2.175789	-0.133767
H	-2.455552	-2.184842	0.137752
H	1.256355	-4.333462	-0.076823
C	-1.466449	0.332291	0.070635
H	-1.212577	0.921790	0.958787
H	-1.229913	0.955693	-0.798705
H	-2.540637	0.155623	0.077352

***o*-xylene-1⊂MCav**

C	-0.097265	3.762730	-2.486425
C	1.118188	3.174803	-2.849301
C	1.054592	1.958072	-3.541614
C	-0.158914	1.378145	-3.895179
C	-1.342869	2.017315	-3.540580
C	-1.341373	3.226037	-2.834082
H	-0.075613	4.700740	-1.946258
H	-0.175105	0.477711	-4.494406
C	-2.645628	3.933121	-2.484757
H	-3.373299	3.634307	-3.240631
C	2.454656	3.856143	-2.581063
H	3.129472	3.527337	-3.373095
C	-3.163747	3.420187	-1.150260
C	-4.137066	2.418289	-1.100808
C	-2.661327	3.881746	0.073693
C	-4.615488	1.925714	0.102846
C	-3.062852	3.356928	1.309330
H	-1.887556	4.641469	0.062295
C	-4.040288	2.359733	1.287436
H	-5.409944	1.195853	0.113803
C	3.063560	3.376424	-1.271080
C	2.661401	3.883111	-0.028000
C	4.041125	2.379124	-1.262935
C	3.162621	3.403084	1.189338
H	1.887745	4.642719	-0.005907
C	4.615351	1.927472	-0.084389
C	4.136085	2.402116	1.125890
H	5.409384	1.197430	-0.106386
C	-2.454934	3.818040	2.626356
H	-3.130705	3.478995	3.413207
C	2.643589	3.895056	2.531296
C	-1.119515	3.131594	2.885452
C	0.095983	3.723535	2.529760
C	-1.056481	1.905366	3.560888
C	1.339934	3.181662	2.869244
H	0.074472	4.668879	2.002661
C	0.157109	1.318745	3.903527
C	1.341251	1.962482	3.557457
H	0.173735	0.409474	4.489329
H	3.371232	3.585551	3.282859
O	2.243273	1.384344	-4.016718
O	4.438751	1.771018	-2.467885
O	-2.563894	1.508237	-4.011441
O	-4.637728	1.860260	-2.290896
O	-4.438153	1.736980	2.484555
O	-2.244970	1.328271	4.032945
O	2.562007	1.447177	4.022489
O	4.635982	1.827509	2.308213
C	2.770070	0.282538	-3.376487
C	3.949037	0.481264	-2.599706
C	2.257325	-0.982364	-3.542596
C	4.579432	-0.576516	-1.989739
C	2.885384	-2.090819	-2.923704
H	1.380813	-1.156381	-4.153988
C	4.047910	-1.874236	-2.118717
H	5.479816	-0.422392	-1.411259
C	4.200444	0.530717	2.525704
C	4.812003	-0.538308	1.912142
C	3.101151	0.332794	3.413789
C	4.339570	-1.844164	2.155565
H	5.647880	-0.392707	1.240359
C	2.672523	-0.938705	3.711374
C	3.284648	-2.057820	3.097052
H	1.872178	-1.107816	4.420123
C	-3.098920	0.384495	-3.416681
C	-2.663293	-0.881640	-3.726684
C	-4.198825	0.567601	-2.526373
C	-3.267519	-2.009948	-3.121742
H	-1.861648	-1.039744	-4.436399

C	-4.806243	-0.511022	-1.925557	C	4.109513	-4.145368	-1.666809
C	-4.325685	-1.811614	-2.180597	Pd	6.190075	-2.730238	-0.108195
H	-5.640268	-0.375794	-1.249453	N	7.828765	-2.543771	1.145765
C	-3.952260	0.444811	2.604228	H	7.796584	-3.209963	1.916999
C	-4.585508	-0.605104	1.983634	N	7.618577	-2.553472	-1.601727
C	-2.777021	0.234282	3.383619	H	7.789077	-3.483284	-1.987891
C	-4.062568	-1.906880	2.106430	C	8.863777	-2.008418	-0.986679
H	-5.483057	-0.439719	1.403521	C	9.071641	-2.710972	0.337345
C	-2.274406	-1.035198	3.545980	H	7.294757	-1.958883	-2.364674
C	-2.907694	-2.136228	2.918887	H	7.787180	-1.606690	1.550798
H	-1.402695	-1.218080	4.161723	C	4.426402	-4.133976	1.802298
N	-2.414776	-3.389981	3.116323	C	2.992233	-4.335137	-2.512566
N	-4.639279	-2.965930	1.453988	H	4.565085	-4.978854	-1.145667
N	-4.844060	-2.900325	-1.528062	H	2.608827	-5.339829	-2.658249
N	2.380236	-3.339457	-3.122165	H	4.842866	-4.976988	1.263984
N	4.865699	-2.924264	1.494455	H	3.108523	-5.324251	3.030952
N	-2.827018	-3.256161	-3.448010	C	-4.390536	-4.103875	-1.839696
N	4.623065	-2.940750	-1.477675	H	-3.058141	-5.274672	-3.071367
N	2.856624	-3.310033	3.416930	H	-4.799875	-4.953709	-1.306490
C	-2.374624	5.353372	2.732360	C	-3.029795	-4.378161	2.497581
H	-1.875869	5.599214	3.676509	H	-4.593512	-5.002346	1.111056
H	-1.747764	5.766732	1.935679	H	-2.656167	-5.386593	2.642813
C	2.550761	5.431338	2.595954	Pd	-6.185467	-2.732654	0.065068
H	3.528927	5.828158	2.302496	N	-7.627640	-2.578346	1.548882
H	1.836339	5.811654	1.858848	H	-7.487906	-3.261250	2.293051
C	2.181823	5.946000	3.987110	N	-7.812971	-2.537603	-1.199997
H	2.191281	7.040208	4.011128	H	-8.034103	-3.462331	-1.572831
H	1.183154	5.613935	4.287010	C	-8.958247	-2.007087	-0.404894
H	2.892968	5.586100	4.739082	C	-8.974099	-2.731399	0.923927
C	2.375455	5.392790	-2.665819	H	-7.528197	-1.650605	1.964806
H	1.875391	5.652042	-3.605676	H	-7.601245	-1.929532	-1.991181
H	1.750154	5.795365	-1.862449	H	-9.897663	-2.148828	-0.945118
C	-2.554229	5.470228	-2.525794	H	-8.790346	-0.937554	-0.259948
H	-3.532530	5.861725	-2.225728	H	-9.148960	-3.799523	0.776853
H	-1.839620	5.839512	-1.783308	H	-9.749222	-2.333390	1.583323
C	3.755417	6.047492	-2.611900	H	8.713684	-0.937106	-0.834961
H	4.399358	5.674557	-3.416232	H	9.717177	-2.156292	-1.653126
H	3.675611	7.133631	-2.720197	H	9.933264	-2.303969	0.872181
H	4.256911	5.842279	-1.660745	H	9.223671	-3.781818	0.184512
C	-2.186490	6.006313	-3.909150	C	-1.381245	-0.346552	-0.168305
H	-2.196562	7.100755	-3.916566	C	-0.693669	0.865983	-0.080659
H	-1.187854	5.679380	-4.214771	C	0.691660	0.865951	0.081726
H	-2.897929	5.657475	-4.666045	C	1.378741	-0.346622	0.172520
C	-3.754008	6.009841	2.685285	C	0.697161	-1.565680	0.097654
H	-4.399823	5.625766	3.482844	C	-0.700205	-1.565718	-0.089935
H	-3.673600	7.094232	2.809497	H	-2.458663	-0.348437	-0.301211
H	-4.253758	5.818846	1.730265	H	2.456128	-0.348934	0.305422
C	-3.390945	-4.271553	-2.825175	C	-1.433458	-2.871390	-0.220869
C	-4.137296	-4.175390	1.641892	H	-1.202097	-3.550788	0.603621
C	3.430412	-4.316597	2.788807	H	-2.509774	-2.711547	-0.239254

H	-1.150862	-3.392414	-1.142270
C	1.430279	-2.870940	0.232986
H	1.155932	-3.383629	1.161486
H	1.190618	-3.557374	-0.583217
H	2.506776	-2.711816	0.239179
H	-1.234049	1.801010	-0.145688
H	1.232261	1.800947	0.144802

***o*-xylene-2⊂MCav**

Pd	4.949948	-2.946176	-0.144305
Pd	-4.947106	-2.948835	-0.146912
C	-0.000142	4.026156	-2.335634
C	1.231479	3.556954	-2.798889
C	1.203560	2.546721	-3.768901
C	0.001415	2.064035	-4.276864
C	-1.201476	2.545669	-3.769741
C	-1.231029	3.555900	-2.799762
H	-0.000707	4.804908	-1.582118
H	0.002015	1.301872	-5.045078
C	-2.531516	4.155529	-2.283585
H	-3.299995	3.901890	-3.014947
C	2.531047	4.157562	-2.281521
H	3.300374	3.904758	-3.012281
C	-2.957567	3.509521	-0.971747
C	-3.852359	2.436057	-1.002007
C	-2.533994	3.954876	0.288293
C	-4.353385	1.860008	0.155711
C	-2.963547	3.358287	1.482818
H	-1.845612	4.790659	0.341738
C	-3.859331	2.291034	1.378528
H	-5.117969	1.099620	0.106834
C	2.956352	3.511518	-0.969466
C	2.531192	3.956214	0.290263
C	3.851833	2.438594	-0.999137
C	2.959834	3.359499	1.485045
H	1.842226	4.791546	0.343283
C	4.351881	1.862420	0.158923
C	3.856334	2.292809	1.381370
H	5.116842	1.102379	0.110575
C	-2.536844	3.839446	2.864573
H	-3.303925	3.493699	3.558621
C	2.531530	3.840098	2.866503
C	-1.234358	3.185037	3.300476
C	-0.002663	3.712975	2.902795
C	-1.205342	2.057494	4.131501
C	1.228876	3.185206	3.301168
H	-0.002529	4.577216	2.250268
C	-0.003008	1.513326	4.571839
C	1.199485	2.057508	4.131976
H	-0.003085	0.658122	5.234976
H	3.298077	3.494478	3.561206
O	2.406240	1.997308	-4.259582
O	4.261701	1.935090	-2.243090
O	-2.403330	1.995115	-4.261206
O	-4.260542	1.931841	-2.246244
O	-4.273387	1.636736	2.549692
O	-2.407913	1.450979	4.550108
O	2.401857	1.450939	4.550985
O	4.269538	1.638332	2.552737
C	2.746116	0.769580	-3.706667
C	3.666597	0.745517	-2.617518
C	2.228469	-0.408427	-4.193627
C	3.959073	-0.433047	-1.971569
C	2.531351	-1.639260	-3.559561
H	1.557864	-0.412087	-5.043908
C	3.363639	-1.633519	-2.398630
H	4.647328	-0.453145	-1.139147
C	3.672009	0.411884	2.775705
C	3.969119	-0.678326	1.991379
C	2.742716	0.301189	3.851380
C	3.357955	-1.917454	2.252246
H	4.669368	-0.602347	1.172207
C	2.216738	-0.926702	4.181805
C	2.514053	-2.067158	3.394817
H	1.538296	-1.032918	5.019010
C	-2.742534	0.767255	-3.708165
C	-2.223419	-0.410462	-4.194240
C	-3.663919	0.742722	-2.619770
C	-2.525811	-1.641383	-3.560057
H	-1.552051	-0.413833	-5.043925
C	-3.955788	-0.435858	-1.973647
C	-3.359113	-1.635999	-2.399864
H	-4.644654	-0.456310	-1.141771
C	-3.675701	0.410609	2.773860
C	-3.971112	-0.680020	1.989468
C	-2.747929	0.300700	3.850957
C	-3.359674	-1.918765	2.251663
H	-4.670116	-0.604584	1.169196
C	-2.222273	-0.926889	4.182914
C	-2.517781	-2.067740	3.395819
H	-1.545096	-1.032487	5.021216
N	-1.978937	-3.274477	3.727099
N	-3.570765	-2.994896	1.426855
N	-3.579895	-2.810502	-1.725391
N	2.006785	-2.796545	-4.051259
N	3.571321	-2.993340	1.427663
N	-2.000012	-2.798393	-4.051051
N	3.584488	-2.808006	-1.724159
N	1.975438	-3.274326	3.724946
C	-2.490696	5.376756	2.977932

H	-2.136511	5.623215	3.984925
H	-1.756862	5.797739	2.283201
C	2.484710	5.377372	2.980169
H	1.751552	5.798247	2.284666
H	2.129240	5.623478	3.986798
C	3.848824	6.023508	2.739349
H	3.795852	7.107338	2.882151
H	4.598537	5.628239	3.433919
H	4.205852	5.837542	1.721602
C	2.487134	5.696186	-2.189575
H	1.728381	6.022850	-1.471602
H	3.451072	6.028372	-1.788256
C	-2.488914	5.694211	-2.192114
H	-3.453350	6.025780	-1.791478
H	-1.730840	6.021767	-1.473837
C	2.232930	6.359370	-3.542768
H	2.267773	7.449624	-3.453760
H	2.988233	6.056290	-4.276546
H	1.251097	6.088289	-3.943471
C	-2.234520	6.357010	-3.545469
H	-2.269505	7.447290	-3.456847
H	-1.252603	6.085865	-3.945924
H	-2.989656	6.053578	-4.279278
C	-3.854759	6.022334	2.735328
H	-3.802348	7.106226	2.877866
H	-4.210521	5.835930	1.717219
H	-4.605140	5.626995	3.429139
C	-2.238085	-3.898378	-3.366172
C	-2.985811	-4.142050	1.726007
C	2.204254	-4.274785	2.898784
C	3.020262	-3.912507	-2.181296
N	6.490627	-3.125679	1.230055
H	6.508500	-4.095051	1.551584
N	6.497988	-2.961881	-1.522520
H	6.287407	-3.551831	-2.327344
C	7.742358	-3.411284	-0.832837
C	7.768154	-2.778782	0.541803
H	6.601141	-2.004466	-1.864331
H	6.343707	-2.534332	2.048443
C	2.986787	-4.140970	1.725709
C	2.245007	-3.896512	-3.366416
H	3.184202	-4.831898	-1.632120
H	1.825925	-4.828654	-3.731151
H	3.140814	-4.978653	1.056297
H	1.771289	-5.242027	3.132614
C	-3.014458	-3.914690	-2.181789
H	-1.818033	-4.830301	-3.730340
H	-3.178315	-4.834103	-1.632626
C	-2.205370	-4.275174	2.900523
H	-3.137906	-4.979864	1.056325
H	-1.772042	-5.242042	3.135215
N	-6.489421	-3.128098	1.225676
H	-6.507383	-4.097348	1.547575
N	-6.493565	-2.965552	-1.526986
H	-6.281800	-3.555680	-2.331365
C	-7.738564	-3.415139	-0.838595
C	-7.766225	-2.781975	0.535693
H	-6.343918	-2.536423	2.044076
H	-6.596646	-2.008275	-1.869196
H	-7.695710	-4.503609	-0.759601
H	-8.619992	-3.136650	-1.421470
H	-8.622639	-3.132841	1.116965
H	-7.812755	-1.693547	0.457591
H	8.624364	-3.132195	-1.414555
H	7.699836	-4.499808	-0.754396
H	7.814417	-1.690303	0.464312
H	8.623980	-3.129678	1.123924
C	0.000146	-3.531162	0.478802
C	-0.001756	-2.369152	1.254268
C	-0.001631	-1.099328	0.671552
C	0.000629	-0.991637	-0.730523
C	0.002426	-2.157970	-1.500516
C	0.002217	-3.424673	-0.910942
C	-0.004152	0.135905	1.522879
H	-0.003734	-0.122338	2.578103
H	0.874085	0.761190	1.325272
H	-0.885083	0.757393	1.325087
H	-0.003187	-2.444093	2.332290
H	0.004132	-2.068058	-2.577732
H	-0.000027	-4.504952	0.957927
C	0.001150	0.359699	-1.381912
H	-0.878520	0.945815	-1.091717
H	0.880221	0.945902	-1.090018
H	0.002223	0.266111	-2.464092
H	0.003649	-4.314339	-1.532273

***o*-xylene-3⊂MCav**

C	-1.774407	-1.968335	-0.751461
C	-1.849795	-0.576808	-0.723993
C	-0.759030	0.168308	-0.280722
C	0.396824	-0.465568	0.183488
C	0.441475	-1.874668	0.227764
C	-0.631621	-2.607731	-0.279576
Pd	5.982885	-2.784476	-0.404154
Pd	-5.999145	-2.745195	0.362415
C	-0.387625	3.776277	-2.475486
C	0.759279	3.101115	-2.901142
C	0.569551	1.880138	-3.563207
C	-0.701103	1.403263	-3.881211
C	-1.814565	2.145592	-3.492881

C	-1.681787	3.328454	-2.756306	H	5.136827	-0.553509	-1.690584
H	-0.268890	4.705848	-1.933778	C	4.569285	0.701699	2.325737
H	-0.806972	0.499486	-4.467672	C	5.050527	-0.416206	1.683669
C	-2.909548	4.062403	-2.243347	C	3.679661	0.571239	3.436945
H	-3.732238	3.836822	-2.922317	C	4.678139	-1.697000	2.140866
C	2.147613	3.713078	-2.764948	H	5.696718	-0.331769	0.819669
H	2.729074	3.322736	-3.601773	C	3.405506	-0.667422	3.964191
C	-3.264064	3.459681	-0.891557	C	3.899572	-1.833460	3.332141
C	-4.253938	2.479734	-0.774137	H	2.775345	-0.770141	4.838718
C	-2.572522	3.821962	0.271346	C	-3.689427	0.645835	-3.429389
C	-4.565027	1.908741	0.452126	C	-3.418411	-0.587750	-3.968474
C	-2.822464	3.236640	1.516534	C	-4.631332	0.775144	-2.360367
H	-1.796308	4.575915	0.198528	C	-3.964520	-1.754298	-3.383817
C	-3.817384	2.259131	1.567646	H	-2.750474	-0.688931	-4.814579
H	-5.368927	1.194152	0.539185	C	-5.185287	-0.344159	-1.778559
C	2.873119	3.269563	-1.503403	C	-4.799966	-1.625295	-2.230253
C	2.625562	3.844265	-0.250791	H	-5.888573	-0.259712	-0.959993
C	3.861834	2.285540	-1.567442	C	-3.480401	0.304894	2.748567
C	3.298859	3.450230	0.912285	C	-4.130216	-0.716539	2.101332
H	1.856581	4.605320	-0.173424	C	-2.208968	0.096945	3.361644
C	4.605025	1.918689	-0.454442	C	-3.534150	-1.986035	2.015131
C	4.276570	2.460766	0.779755	H	-5.087476	-0.543536	1.629793
H	5.406719	1.201251	-0.545398	C	-1.620948	-1.146528	3.309441
C	-2.104193	3.666402	2.786278	C	-2.269569	-2.217667	2.643174
H	-2.694620	3.273021	3.615313	H	-0.663255	-1.325945	3.783481
C	2.948322	4.017857	2.282812	N	-1.688722	-3.448096	2.625748
C	-0.719829	3.049241	2.932800	N	-4.142689	-3.000954	1.322386
C	0.430469	3.722989	2.515077	N	-5.157303	-2.760923	-1.549572
C	-0.536820	1.828739	3.599375	N	1.672976	-3.397639	-2.662784
C	1.724795	3.278990	2.804109	N	5.025774	-2.826765	1.447080
H	0.313759	4.648733	1.966310	N	-3.630757	-2.970931	-3.895063
C	0.732134	1.363888	3.938266	N	4.172967	-3.019044	-1.434666
C	1.849786	2.103903	3.554083	N	3.575015	-3.054409	3.838929
H	0.835480	0.457279	4.521339	C	-2.088314	5.201957	2.938855
H	3.778311	3.779020	2.948416	H	-1.528644	5.443198	3.849190
O	1.694330	1.204913	-4.061381	H	-1.546679	5.667517	2.109284
O	4.086997	1.599593	-2.777352	C	2.794101	5.549535	2.274943
O	-3.102037	1.786255	-3.933995	H	1.992880	5.857802	1.595740
O	-4.952149	2.048090	-1.918522	H	2.484394	5.860521	3.278873
O	-4.045853	1.570903	2.774407	C	4.089989	6.259279	1.883684
O	-1.663193	1.142521	4.081457	H	3.968857	7.345953	1.930300
O	3.133177	1.714962	3.986208	H	4.909679	5.981592	2.555728
O	4.930182	1.976962	1.926485	H	4.392595	6.000122	0.863811
C	2.235476	0.145394	-3.362479	C	2.136751	5.249430	-2.906460
C	3.516366	0.334593	-2.762436	H	1.570965	5.499631	-3.810585
C	1.630275	-1.089474	-3.316111	H	1.603593	5.711272	-2.069325
C	4.168451	-0.706978	-2.147255	C	-2.756543	5.592991	-2.199377
C	2.277280	-2.177430	-2.679567	H	-1.961243	5.886910	-1.507044
H	0.660625	-1.250905	-3.771096	H	-2.439724	5.927237	-3.193605
C	3.560812	-1.974549	-2.081801	C	3.545708	5.835096	-2.996017

H	4.114319	5.650712	-2.078929
H	4.103370	5.393054	-3.829346
H	3.506895	6.917521	-3.152985
C	-4.056560	6.291314	-1.801429
H	-4.871064	6.027482	-2.485227
H	-4.365540	6.008206	-0.789780
H	-3.937357	7.378981	-1.822264
C	-3.495425	5.793025	3.021752
H	-3.452975	6.874025	3.187404
H	-4.057648	5.618456	2.098823
H	-4.061583	5.347122	3.847252
C	-4.066433	-4.031707	-3.245316
C	-3.538895	-4.177149	1.276908
C	3.965703	-4.109173	3.151748
C	3.560885	-4.190547	-1.409303
N	7.835431	-2.533751	0.490754
H	7.947917	-3.123428	1.315009
N	7.101634	-2.800270	-2.150957
H	7.187853	-3.770882	-2.456829
C	8.445260	-2.230734	-1.841899
C	8.899173	-2.807793	-0.518520
H	6.647433	-2.280796	-2.902297
H	7.877998	-1.561963	0.802926
C	4.675814	-4.006393	1.933775
C	2.307613	-4.370703	-2.041516
H	4.043412	-5.004833	-0.882223
H	1.841739	-5.350463	-2.012311
H	4.943611	-4.889583	1.365800
H	3.717245	-5.093501	3.535182
C	-4.805607	-3.937588	-2.044033
H	-3.820117	-5.012905	-3.637857
H	-5.082631	-4.824522	-1.486841
C	-2.314275	-4.393055	1.952310
H	-4.014286	-4.971578	0.713703
H	-1.864668	-5.380197	1.917302
N	-7.007327	-2.795598	2.174141
H	-7.069104	-3.772618	2.465597
N	-7.902758	-2.499278	-0.412122
H	-8.060269	-3.079273	-1.235912
C	-8.900767	-2.795853	0.656698
C	-8.371226	-2.231706	1.957205
H	-6.513378	-2.288229	2.908511
H	-7.973210	-1.523758	-0.708034
H	-9.002002	-3.881519	0.721605
H	-9.871845	-2.364126	0.402383
H	-9.026789	-2.481794	2.795073
H	-8.272335	-1.145729	1.895365
H	8.334248	-1.146377	-1.771787
H	9.153614	-2.464191	-2.640638
H	9.849430	-2.371834	-0.200805
H	9.013750	-3.891702	-0.590875
H	-2.603266	-2.566508	-1.100033
H	-2.747349	-0.069555	-1.038454
H	-0.805995	1.254406	-0.287339
C	1.601755	-2.581800	0.865367
H	1.586999	-2.426286	1.949588
H	1.564470	-3.657672	0.683275
H	2.556313	-2.197756	0.505394
C	1.578175	0.341435	0.627968
H	1.862368	0.089203	1.649921
H	2.449943	0.141052	-0.001596
H	1.366871	1.409941	0.585159
H	-0.581293	-3.692302	-0.275145

***m*-xylene⊂MCav**

C	0.828249	-1.822955	0.021477
C	0.559095	-0.448688	0.006385
C	-0.771293	-0.011921	0.003135
C	-1.805231	-0.942378	0.016092
C	-1.521734	-2.308577	0.032900
C	-0.201057	-2.769973	0.035071
H	-2.830073	-0.598164	0.012430
Pd	5.817799	-2.950286	-0.036064
Pd	-6.016349	-2.642514	-0.038225
C	0.139409	3.875140	-2.489851
C	1.362341	3.305893	-2.865537
C	1.307251	2.133687	-3.628661
C	0.100591	1.555620	-4.008956
C	-1.087866	2.146714	-3.600335
C	-1.098524	3.332810	-2.855973
H	0.150387	4.790492	-1.911565
H	0.081875	0.690149	-4.655615
C	-2.418316	4.007404	-2.513151
H	-3.125313	3.719989	-3.292894
C	2.706867	3.938847	-2.519317
H	3.403122	3.625460	-3.298720
C	-2.967847	3.460810	-1.205157
C	-3.968530	2.485632	-1.184474
C	-2.494143	3.913678	0.030450
C	-4.499108	2.010856	0.006774
C	-2.970301	3.432065	1.254038
H	-1.707731	4.660172	0.039684
C	-3.970226	2.456967	1.209475
H	-5.306647	1.294225	-0.002699
C	3.254527	3.389513	-1.206730
C	2.813310	3.872984	0.031131
C	4.208334	2.366263	-1.183051
C	3.250680	3.361164	1.258588
H	2.064385	4.656951	0.039128
C	4.723818	1.880969	0.010957

C	4.204553	2.338824	1.213922	H	-5.475282	-0.330919	1.370482
H	5.484961	1.115270	0.002644	C	-2.359226	-0.863592	3.664687
C	-2.421959	3.945055	2.575596	C	-2.969839	-1.982395	3.046694
H	-3.130307	3.639992	3.347302	H	-1.511301	-1.030592	4.316803
C	2.698177	3.875903	2.583229	N	-2.470351	-3.226283	3.281443
C	-1.104936	3.257366	2.899936	N	-4.611040	-2.846745	1.502407
C	0.132915	3.806767	2.545883	N	-4.551604	-2.804151	-1.533410
C	-1.096043	2.052030	3.612640	N	2.102673	-3.122793	-3.209594
C	1.355214	3.229849	2.909868	N	4.365253	-3.027679	1.455906
H	0.144391	4.735404	1.989400	N	-2.365406	-3.132351	-3.271079
C	0.092489	1.447727	4.002961	N	4.304376	-2.979835	-1.472537
C	1.299685	2.038671	3.643185	N	2.193754	-3.226005	3.222748
H	0.073392	0.563780	4.624452	C	-2.335433	5.482621	2.622900
H	3.393682	3.544954	3.355993	H	-1.890513	5.762594	3.584203
O	2.509145	1.571815	-4.086081	H	-1.659893	5.860021	1.848838
O	4.629602	1.756072	-2.382469	C	2.654597	5.415263	2.645217
O	-2.306975	1.613828	-4.046535	H	1.992460	5.818435	1.872237
O	-4.443862	1.935426	-2.390584	H	2.212844	5.697761	3.607211
O	-4.449668	1.883157	2.402443	C	4.040892	6.043341	2.504405
O	-2.315961	1.517626	4.055581	H	3.987214	7.131579	2.607500
O	2.501171	1.475701	4.100275	H	4.724289	5.664833	3.272831
O	4.622400	1.704909	2.401331	H	4.482143	5.821611	1.527330
C	2.929722	0.417958	-3.457379	C	2.667830	5.479492	-2.541383
C	4.057754	0.507823	-2.590061	H	2.228967	5.788398	-3.496570
C	2.310793	-0.789073	-3.673538	H	2.005089	5.863748	-1.759441
C	4.547159	-0.616019	-1.964087	C	-2.336232	5.545983	-2.520768
C	2.761129	-1.948951	-3.006303	H	-1.663024	5.904335	-1.735665
H	1.468027	-0.879252	-4.345278	H	-1.890412	5.852210	-3.473605
C	3.892254	-1.854254	-2.137520	C	4.055587	6.099626	-2.381371
H	5.409678	-0.557713	-1.313093	H	4.494449	5.850198	-1.409929
C	4.059984	0.449584	2.587328	H	4.739176	5.740068	-3.158673
C	4.560354	-0.660699	1.946612	H	4.005116	7.190400	-2.455065
C	2.937586	0.334850	3.459665	C	-3.706791	6.200978	-2.351786
C	3.932823	-1.912196	2.124132	H	-4.401022	5.868103	-3.131495
H	5.418702	-0.586000	1.291479	H	-4.150364	5.952773	-1.382055
C	2.344093	-0.884585	3.677338	H	-3.627542	7.290750	-2.413624
C	2.815718	-2.034624	3.007224	C	-3.703929	6.145596	2.468716
H	1.510994	-0.991958	4.358759	H	-3.621925	7.233120	2.559755
C	-2.817370	0.485811	-3.436031	H	-4.146210	5.924578	1.491831
C	-2.308325	-0.770941	-3.662258	H	-4.400721	5.793962	3.237837
C	-3.970106	0.651105	-2.611157	C	-2.922250	-4.147803	-2.641210
C	-2.901816	-1.901535	-3.049107	C	-4.109965	-4.049893	1.736536
H	-1.450089	-0.922773	-4.304347	C	2.664312	-4.269648	2.571503
C	-4.582761	-0.431659	-2.025669	C	3.652137	-4.113322	-1.675527
C	-4.040947	-1.720887	-2.201860	N	7.392865	-2.928254	1.313547
H	-5.450190	-0.294638	-1.392968	H	7.255644	-3.590905	2.076191
C	-3.988422	0.591705	2.604399	N	7.344487	-2.911999	-1.439053
C	-4.611818	-0.478275	2.006437	H	7.448462	-3.853978	-1.819196
C	-2.842105	0.402896	3.434538	C	8.597345	-2.487211	-0.750307
C	-4.091223	-1.776126	2.182687	C	8.660263	-3.209144	0.578275

H	7.122718	-2.289724	-2.216384
H	7.415852	-1.993087	1.724397
C	3.755985	-4.181100	1.678679
C	2.546604	-4.174535	-2.553326
H	3.991096	-4.998090	-1.151932
H	2.038418	-5.122644	-2.694745
H	4.117415	-5.057030	1.154223
H	2.187438	-5.231810	2.726792
C	-4.009949	-3.992563	-1.751617
H	-2.514063	-5.140017	-2.803776
H	-4.411412	-4.840237	-1.209441
C	-3.045581	-4.230413	2.649361
H	-4.525733	-4.887276	1.188688
H	-2.668943	-5.232564	2.826387
N	-7.580659	-2.532663	1.313048
H	-7.752694	-3.474525	1.668424
N	-7.536358	-2.487501	-1.440005
H	-7.412578	-3.142930	-2.211231
C	-8.843465	-2.703542	-0.753335
C	-8.782079	-2.017588	0.594082
H	-7.346522	-1.941008	2.110349
H	-7.488338	-1.545138	-1.831825
H	-8.976914	-3.780661	-0.630419
H	-9.663191	-2.312515	-1.360966
H	-9.687063	-2.205897	1.177123
H	-8.655936	-0.939396	0.473042
H	8.540116	-1.406919	-0.599525
H	9.470622	-2.713883	-1.367087
H	9.523359	-2.886065	1.165477
H	8.720307	-4.289117	0.425630
H	1.860499	-2.160958	0.021186
H	-2.325357	-3.032541	0.042572
H	-0.992294	1.052842	-0.009776
C	1.672080	0.550271	-0.003621
H	2.644278	0.062367	-0.020639
H	1.601206	1.203883	-0.876198
H	1.626833	1.190195	0.880648
C	0.088905	-4.249435	0.050851
H	-0.361617	-4.746255	-0.816458
H	1.163519	-4.447322	0.035761
H	-0.331354	-4.722422	0.946257

***m*-xylene-1⊂MCav**

Pd	-4.995102	-2.963434	-0.069853
Pd	5.036797	-2.926323	-0.137077
C	0.016049	3.789744	2.512765
C	-1.208358	3.285880	2.953976
C	-1.169495	2.191636	3.828200
C	0.041089	1.652652	4.260277
C	1.239181	2.205830	3.812145
C	1.251981	3.300558	2.938308
H	0.007147	4.631100	1.831235
H	0.050350	0.822522	4.955785
C	2.537201	3.979227	2.487397
H	3.310330	3.683446	3.197346
C	-2.507326	3.952915	2.523832
H	-3.265145	3.649067	3.246921
C	2.971404	3.452069	1.128154
C	3.878063	2.390376	1.073527
C	2.529582	3.982966	-0.090465
C	4.376847	1.911827	-0.126987
C	2.950470	3.480812	-1.331198
H	1.830053	4.810763	-0.074986
C	3.863024	2.422657	-1.311387
H	5.151109	1.159664	-0.142937
C	-2.965606	3.427009	1.171397
C	-2.563382	3.971532	-0.054849
C	-3.862404	2.356117	1.132654
C	-3.013312	3.473635	-1.287279
H	-1.872311	4.806544	-0.052656
C	-4.386798	1.878638	-0.057423
C	-3.912310	2.404466	-1.251446
H	-5.152248	1.117436	-0.057501
C	2.495181	4.066446	-2.664219
H	3.245810	3.768595	-3.397870
C	-2.594452	4.067470	-2.628377
C	1.178445	3.459368	-3.129080
C	-0.051119	3.975458	-2.706255
C	1.139764	2.376632	-4.016978
C	-1.286159	3.463288	-3.118251
H	-0.047680	4.808525	-2.014297
C	-0.063548	1.866998	-4.489827
C	-1.261212	2.383379	-4.009486
H	-0.067036	1.060141	-5.209784
H	-3.360107	3.769365	-3.346027
O	-2.368567	1.651788	4.339064
O	-4.248596	1.763703	2.342627
O	2.452561	1.682452	4.306412
O	4.298980	1.806583	2.276455
O	4.265494	1.835444	-2.523154
O	2.336175	1.775244	-4.454430
O	-2.465409	1.790690	-4.439667
O	-4.344155	1.825601	-2.457031
C	-2.777476	0.471078	3.736947
C	-3.678679	0.540731	2.632282
C	-2.342603	-0.748398	4.195512
C	-3.990240	-0.588507	1.914112
C	-2.672141	-1.933793	3.490789
H	-1.694125	-0.813260	5.060700
C	-3.449553	-1.830719	2.296010

H	-4.658245	-0.544602	1.066532	H	-2.160598	7.122585	3.976605
C	-3.727413	0.621347	-2.747769	H	-2.896668	5.676032	4.685990
C	-4.016690	-0.518674	-2.033180	H	-1.164978	5.703627	4.325609
C	-2.771902	0.595673	-3.804044	C	2.180147	6.054926	3.931874
C	-3.347391	-1.721086	-2.328060	H	2.188000	7.149346	3.938101
H	-4.746357	-0.502921	-1.236133	H	1.204016	5.726220	4.302145
C	-2.181399	-0.592280	-4.167271	H	2.940398	5.707853	4.640636
C	-2.443491	-1.774601	-3.432923	C	3.838601	6.220407	-2.388907
H	-1.476188	-0.638067	-4.986960	H	3.794975	7.312595	-2.444496
C	2.867992	0.504029	3.704566	H	4.211032	5.950967	-1.395678
C	2.460073	-0.718147	4.180457	H	4.571796	5.874096	-3.125928
C	3.749587	0.578073	2.583893	C	2.588147	-4.158483	3.178641
C	2.791911	-1.904047	3.477434	C	2.869676	-3.945874	-1.875632
H	1.828626	-0.785423	5.057978	C	-2.018300	-3.978178	-3.009038
C	4.064289	-0.552300	1.868489	C	-3.170088	-4.105341	1.961479
C	3.545241	-1.798942	2.267321	N	-6.504018	-3.028120	-1.492345
H	4.718942	-0.506246	1.010391	H	-6.534592	-3.974083	-1.876284
C	3.641299	0.627508	-2.783129	N	-6.578375	-3.097720	1.261087
C	3.958551	-0.504981	-2.068859	H	-6.394818	-3.769083	2.006794
C	2.652142	0.589321	-3.807123	C	-7.814525	-3.452814	0.505501
C	3.289079	-1.713601	-2.334154	C	-7.789117	-2.702545	-0.808641
H	4.714902	-0.478971	-1.297869	H	-6.673294	-2.177997	1.695883
C	2.047291	-0.602493	-4.133848	H	-6.330205	-2.389089	-2.268545
C	2.342925	-1.778544	-3.402209	C	-2.878781	-3.946853	-1.885925
H	1.313545	-0.657905	-4.927393	C	-2.447539	-4.183512	3.174222
N	1.714389	-2.942218	-3.724142	H	-3.345053	-4.985316	1.354380
N	3.533016	-2.839673	-1.586969	H	-2.073757	-5.148342	3.501195
N	3.769133	-2.925062	1.519852	H	-3.026520	-4.825120	-1.269472
N	-2.209773	-3.132462	3.935323	H	-1.511693	-4.905082	-3.257836
N	-3.547730	-2.850194	-1.572982	C	3.283403	-4.078753	1.950249
N	2.352766	-3.105485	3.938055	H	2.232705	-5.125611	3.519031
N	-3.677209	-2.955072	1.547247	H	3.453686	-4.959633	1.343091
N	-1.814364	-2.930335	-3.780803	C	1.964453	-3.987732	-2.963375
C	2.463437	5.608384	-2.654997	H	3.056653	-4.823271	-1.268668
H	2.092911	5.937552	-3.632001	H	1.462282	-4.921697	-3.193918
H	1.748116	5.980080	-1.914690	N	6.502103	-2.976041	-1.606170
C	-2.565833	5.609089	-2.615255	H	6.536271	-3.923413	-1.986245
H	-1.835030	5.980152	-1.889849	N	6.661827	-3.034707	1.144708
H	-2.217550	5.941904	-3.599209	H	6.510811	-3.708910	1.895059
C	-3.936033	6.217205	-2.316948	C	7.879946	-3.371609	0.352334
H	-3.895775	7.309724	-2.368519	C	7.802092	-2.627099	-0.962919
H	-4.684240	5.872681	-3.039572	H	6.295575	-2.343680	-2.379768
H	-4.286384	5.942468	-1.317135	H	6.756066	-2.113854	1.577216
C	-2.437865	5.492480	2.566756	H	7.874082	-4.450580	0.182499
H	-1.688136	5.870927	1.864795	H	8.782901	-3.105235	0.907073
H	-3.403745	5.873374	2.216580	H	8.636794	-2.890243	-1.617535
C	2.454935	5.518150	2.527602	H	7.808024	-1.547619	-0.796632
H	3.412133	5.906453	2.162073	H	-8.704411	-3.203286	1.088465
H	1.691275	5.888472	1.836431	H	-7.795867	-4.530935	0.331332
C	-2.147286	6.028234	3.968232	H	-7.807208	-1.623862	-0.637895

H	-8.638992	-2.976836	-1.438731
C	0.005640	-2.886152	0.292856
C	-0.050966	-1.872412	-0.672738
C	-0.063899	-0.515761	-0.315101
C	-0.015285	-0.171455	1.041860
C	0.042832	-1.170297	2.009789
C	0.052651	-2.514960	1.639896
C	-0.137871	0.560138	-1.360399
H	-0.074047	0.143136	-2.363718
H	-1.078747	1.116108	-1.286599
H	0.672875	1.285694	-1.239759
H	-0.084745	-2.139802	-1.722990
H	0.077547	-0.900501	3.055749
H	-0.023128	0.875062	1.335544
H	0.093308	-3.283681	2.400496
C	0.013919	-4.348333	-0.075032
H	-0.016586	-4.497331	-1.155210
H	0.912438	-4.843253	0.311254
H	-0.847574	-4.865624	0.362431

***m*-xylene-2⊂MCav**

Pd	5.791005	-2.728948	-0.561985
Pd	-5.769887	-2.849758	0.116004
C	-0.359078	4.004420	-2.187634
C	0.808346	3.425055	-2.691931
C	0.649947	2.288444	-3.496150
C	-0.605001	1.813672	-3.862119
C	-1.739164	2.459429	-3.382351
C	-1.642109	3.553860	-2.515234
H	-0.267968	4.867921	-1.540098
H	-0.690057	0.980763	-4.547123
C	-2.886787	4.210164	-1.937034
H	-3.702810	4.011215	-2.632767
C	2.182062	4.048686	-2.458723
H	2.796370	3.741397	-3.306747
C	-3.236655	3.513440	-0.631143
C	-4.188138	2.489215	-0.607427
C	-2.611615	3.836689	0.579925
C	-4.537289	1.844853	0.568683
C	-2.890817	3.168691	1.779311
H	-1.865690	4.623822	0.585912
C	-3.849217	2.154405	1.732997
H	-5.327103	1.108820	0.574973
C	2.871638	3.503841	-1.217168
C	2.597231	3.972688	0.075986
C	3.829576	2.493711	-1.336588
C	3.206135	3.443433	1.222601
H	1.846149	4.745781	0.196026
C	4.518362	2.005522	-0.237362
C	4.157805	2.438086	1.028033
H	5.309129	1.282848	-0.363591
C	-2.225984	3.537750	3.097831
H	-2.839653	3.093369	3.883005
C	2.836494	3.886129	2.635821
C	-0.843702	2.916855	3.240562
C	0.313570	3.601835	2.858181
C	-0.673946	1.657935	3.832909
C	1.604452	3.119009	3.097178
H	0.205342	4.561080	2.367786
C	0.589213	1.147104	4.111367
C	1.713117	1.887344	3.755614
H	0.689221	0.209571	4.642937
H	3.659874	3.582010	3.283450
O	1.783510	1.661668	-4.036238
O	4.074296	1.894782	-2.587313
O	-3.006115	2.059376	-3.844713
O	-4.808472	2.088826	-1.802557
O	-4.105212	1.385530	2.882946
O	-1.801394	0.950901	4.281156
O	2.983189	1.423574	4.141598
O	4.763275	1.825886	2.136352
C	2.250261	0.530818	-3.393414
C	3.480138	0.644033	-2.683294
C	1.603314	-0.681611	-3.466785
C	4.045771	-0.452361	-2.077808
C	2.159067	-1.824858	-2.840897
H	0.668796	-0.788113	-4.002516
C	3.397250	-1.699675	-2.138167
H	4.974408	-0.364380	-1.531681
C	4.370317	0.524998	2.393030
C	4.853511	-0.540741	1.669257
C	3.476407	0.313015	3.485661
C	4.495919	-1.852292	2.044550
H	5.527574	-0.399159	0.834843
C	3.185446	-0.960519	3.911888
C	3.709726	-2.077400	3.216649
H	2.563247	-1.127794	4.781943
C	-3.492227	0.849153	-3.391451
C	-3.157341	-0.335118	-4.002438
C	-4.404323	0.860652	-2.293683
C	-3.626952	-1.562957	-3.476472
H	-2.505943	-0.351600	-4.867315
C	-4.876801	-0.317369	-1.763399
C	-4.454468	-1.546387	-2.309800
H	-5.554290	-0.317845	-0.920075
C	-3.514441	0.133558	2.812152
C	-4.089061	-0.863913	2.062351
C	-2.290871	-0.085483	3.509415
C	-3.464702	-2.120950	1.972926
H	-5.011158	-0.682654	1.528955

C	-1.687865	-1.321865	3.468167
C	-2.265877	-2.371583	2.710860
H	-0.773051	-1.514868	4.014773
N	-1.685669	-3.602650	2.717998
N	-3.995391	-3.117113	1.193569
N	-4.769271	-2.736952	-1.705762
N	1.517090	-3.021194	-2.939314
N	4.912746	-2.932205	1.312013
N	-3.222786	-2.729924	-4.049058
N	3.940565	-2.804094	-1.532926
N	3.460174	-3.333573	3.677680
C	-2.223119	5.060943	3.339643
H	-1.692670	5.249616	4.279452
H	-1.657758	5.576575	2.556811
C	2.694285	5.413256	2.770252
H	1.903277	5.791861	2.115083
H	2.374087	5.629719	3.795442
C	4.000280	6.146625	2.465920
H	3.885454	7.224849	2.614304
H	4.807658	5.799920	3.120616
H	4.316675	5.983076	1.430844
C	2.152809	5.591125	-2.463649
H	1.525189	5.969688	-1.650306
H	3.170055	5.936642	-2.249081
C	-2.780055	5.738327	-1.783247
H	-3.708128	6.085829	-1.315997
H	-1.973742	6.005588	-1.092804
C	1.683691	6.171954	-3.797227
H	1.719908	7.265716	-3.778644
H	2.321518	5.827458	-4.619023
H	0.655721	5.875880	-4.027103
C	-2.573867	6.451193	-3.119206
H	-2.558703	7.536988	-2.982541
H	-1.628080	6.160602	-3.587000
H	-3.380883	6.210692	-3.820327
C	-3.635090	5.641151	3.413861
H	-3.603681	6.712214	3.636730
H	-4.169916	5.512359	2.467624
H	-4.221323	5.150258	4.198784
C	-3.581405	-3.846635	-3.447067
C	-3.381017	-4.287463	1.161928
C	3.942036	-4.336626	2.971359
C	3.288319	-3.952177	-1.612904
N	7.689741	-2.636250	0.252908
H	7.942990	-3.575365	0.564872
N	6.817312	-2.584767	-2.362467
H	6.448382	-3.223637	-3.066234
C	8.262074	-2.851200	-2.098619
C	8.632191	-2.162901	-0.802483
H	6.691851	-1.635935	-2.718967
H	7.713308	-2.024993	1.069176
C	4.649933	-4.148673	1.761381
C	2.070841	-4.048130	-2.326718
H	3.719026	-4.815024	-1.119388
H	1.568972	-5.008711	-2.380054
H	4.980431	-4.992197	1.167286
H	3.764069	-5.346999	3.324923
C	-4.337989	-3.862160	-2.252782
H	-3.266546	-4.789139	-3.882963
H	-4.575836	-4.792930	-1.751738
C	-2.230409	-4.525347	1.952022
H	-3.794401	-5.061706	0.526201
H	-1.773942	-5.509689	1.931301
N	-6.927242	-2.978646	1.834231
H	-7.026514	-3.966730	2.073217
N	-7.607851	-2.582876	-0.807093
H	-7.699097	-3.147091	-1.651530
C	-8.691545	-2.894952	0.169893
C	-8.259803	-2.383086	1.527122
H	-6.494101	-2.513863	2.632485
H	-7.651722	-1.602201	-1.090157
H	-8.814491	-3.980059	0.190477
H	-9.632877	-2.438271	-0.145155
H	-8.985615	-2.647562	2.300213
H	-8.138580	-1.297702	1.510294
H	8.875034	-2.490427	-2.928277
H	8.387807	-3.932550	-2.009623
H	8.508302	-1.081433	-0.892812
H	9.665031	-2.378727	-0.518204
C	-0.661989	-2.865747	-0.366239
C	0.488163	-2.644787	0.396996
C	0.909456	-1.324156	0.600641
C	0.200527	-0.235427	0.074215
C	-0.969821	-0.480580	-0.655345
C	-1.388769	-1.791738	-0.871182
H	-1.541038	0.351003	-1.059658
H	-0.994594	-3.881554	-0.555938
H	-2.282773	-1.982783	-1.438275
C	1.228582	-3.802282	1.006847
H	1.019543	-3.875729	2.078685
H	0.934762	-4.748988	0.545501
H	2.305288	-3.687336	0.890898
C	0.722353	1.162493	0.235169
H	1.319672	1.263335	1.141418
H	1.366397	1.420197	-0.610430
H	-0.085272	1.899255	0.263367
H	1.809620	-1.132738	1.180271

***m*-xylene-3⊂MCav**

C	-0.257719	-3.282549	0.011495
C	-1.387608	-2.459019	0.022709
C	-1.235207	-1.067021	0.016812
C	0.046626	-0.532108	0.002794
C	1.179540	-1.336913	-0.008809
C	1.026993	-2.728743	-0.005189
H	0.168217	0.549810	-0.000911
C	-2.360056	-0.089883	0.023645
H	-2.283821	0.556877	0.897640
H	-2.288376	0.564219	-0.845078
H	-3.335252	-0.562807	0.023708
H	1.905848	-3.368563	-0.014643
H	-2.380595	-2.894955	0.034028
Pd	-6.369765	-2.646145	-0.027576
Pd	6.404054	-2.600338	0.003270
C	-0.029148	3.686169	2.500857
C	-1.259418	3.118370	2.843700
C	-1.236755	1.897181	3.529591
C	-0.037308	1.274485	3.879877
C	1.166976	1.887602	3.531397
C	1.196182	3.111789	2.850642
H	-0.024950	4.634893	1.980214
H	-0.039740	0.369865	4.474063
C	2.520865	3.801427	2.564123
H	3.209784	3.494265	3.351692
C	-2.581098	3.812776	2.548529
H	-3.275572	3.506976	3.331759
C	3.102560	3.300117	1.251649
C	4.145241	2.370191	1.221638
C	2.615252	3.756028	0.023458
C	4.703496	1.940696	0.021769
C	3.121453	3.323599	-1.205611
H	1.793320	4.463895	0.023219
C	4.164495	2.393957	-1.178215
H	5.528221	1.242935	0.022947
C	-3.160433	3.318405	1.231317
C	-2.658303	3.768500	0.006311
C	-4.206594	2.391736	1.193074
C	-3.142604	3.323343	-1.227533
H	-1.833981	4.473686	0.013891
C	-4.756520	1.965810	-0.011722
C	-4.189455	2.397029	-1.206760
H	-5.578249	1.264374	-0.019182
C	2.553570	3.845763	-2.516021
H	3.251502	3.553718	-3.301338
C	-2.549529	3.825275	-2.535952
C	1.234763	3.154097	-2.823900
C	0.003637	3.715001	-2.472073
C	1.218421	1.938865	-3.521041
C	-1.220993	3.140885	-2.825094
H	-0.001323	4.658036	-1.941105
C	0.020342	1.318849	-3.878093
C	-1.185183	1.926274	-3.522105
H	0.024552	0.420976	-4.482415
H	-3.234453	3.520017	-3.327796
O	-2.461466	1.397388	4.004796
O	-4.684779	1.834262	2.399108
O	2.387385	1.380248	4.010467
O	4.628258	1.833267	2.434492
O	4.666844	1.882529	-2.394346
O	2.445119	1.449605	-4.003629
O	-2.403354	1.416802	-4.003429
O	-4.647552	1.841931	-2.421567
C	-3.064101	0.314940	3.400096
C	-4.242928	0.535375	2.618625
C	-2.595261	-0.962392	3.593657
C	-4.907617	-0.522976	2.043951
C	-3.240041	-2.063192	2.983508
H	-1.721487	-1.148211	4.205049
C	-4.393395	-1.829792	2.171235
H	-5.786814	-0.350929	1.436065
C	-4.192081	0.546265	-2.632876
C	-4.856112	-0.513954	-2.061711
C	-3.001341	0.332579	-3.397108
C	-4.331709	-1.817665	-2.174115
H	-5.743088	-0.340121	-1.466439
C	-2.517454	-0.941708	-3.572875
C	-3.161681	-2.044193	-2.964752
H	-1.632499	-1.123717	-4.169286
C	3.005765	0.302668	3.412723
C	2.547606	-0.981178	3.592771
C	4.202076	0.531800	2.659802
C	3.216101	-2.073118	2.988909
H	1.659776	-1.177827	4.180586
C	4.896457	-0.518232	2.105383
C	4.389899	-1.829144	2.208926
H	5.784354	-0.330552	1.515630
C	4.251003	0.583755	-2.650971
C	4.942850	-0.473842	-2.107755
C	3.065328	0.363477	-3.423752
C	4.439120	-1.783484	-2.238525
H	5.823178	-0.298173	-1.503043
C	2.615638	-0.918494	-3.635482
C	3.276881	-2.018262	-3.038388
H	1.736368	-1.108106	-4.238098
N	2.768535	-3.268529	-3.213559
N	4.983709	-2.829463	-1.537473
N	4.946690	-2.867141	1.504518
N	-2.738265	-3.313519	3.174648

N	-4.889593	-2.872873	-1.499141
N	2.707847	-3.326147	3.144120
N	-4.945263	-2.883381	1.489640
N	-2.644511	-3.290634	-3.139730
C	2.461837	5.383657	-2.539339
H	1.994802	5.678486	-3.485649
H	1.804433	5.746266	-1.742792
C	-2.464628	5.363661	-2.578870
H	-1.822110	5.739414	-1.776167
H	-1.983130	5.648021	-3.521095
C	-3.840261	6.021739	-2.476227
H	-3.758376	7.109907	-2.559423
H	-4.505119	5.671281	-3.273639
H	-4.319774	5.795008	-1.518379
C	-2.488419	5.350309	2.599704
H	-2.018474	5.627538	3.549859
H	-1.832801	5.726712	1.808040
C	2.435017	5.339360	2.611422
H	1.786033	5.717465	1.815136
H	1.960897	5.620821	3.558261
C	-3.858797	6.016624	2.480846
H	-4.325549	5.797580	1.514935
H	-4.537139	5.665772	3.266630
H	-3.772083	7.103874	2.570891
C	3.809364	5.998652	2.499141
H	4.481389	5.646297	3.289647
H	4.280674	5.775193	1.536433
H	3.727470	7.086519	2.586197
C	3.832751	6.045787	-2.404673
H	3.747792	7.134713	-2.473471
H	4.297176	5.807002	-1.442279
H	4.512173	5.709430	-3.195834
C	3.322249	-4.300176	2.504626
C	4.472262	-4.037760	-1.713944
C	-3.251024	-4.278498	-2.514095
C	-4.443841	-4.093534	1.682940
N	-7.889147	-2.449415	-1.426767
H	-7.785164	-3.107127	-2.198898
N	-7.930182	-2.483767	1.324059
H	-8.126419	-3.418866	1.684794
C	-9.119601	-1.940905	0.606047
C	-9.200565	-2.630137	-0.738491
H	-7.678415	-1.894751	2.117889
H	-7.817259	-1.508556	-1.818282
C	-4.368143	-4.077632	-1.672202
C	-3.345289	-4.300219	2.547335
H	-4.897458	-4.922548	1.154454
H	-2.970645	-5.308742	2.689420
H	-4.819904	-4.909461	-1.147296
H	-2.862795	-5.283963	-2.640617

C	4.433185	-4.077611	1.659339
H	2.941914	-5.310295	2.617190
H	4.884966	-4.897869	1.116254
C	3.372907	-4.250357	-2.576310
H	4.917734	-4.862960	-1.173317
H	2.992672	-5.258665	-2.704385
N	7.958624	-2.396302	-1.349313
H	8.156833	-3.321634	-1.733545
N	7.925970	-2.438994	1.400570
H	7.823904	-3.120554	2.152080
C	9.235904	-2.596176	0.703391
C	9.148097	-1.868539	-0.620093
H	7.704128	-1.788992	-2.128460
H	7.853375	-1.510593	1.820950
H	9.398985	-3.664526	0.544951
H	10.046384	-2.202019	1.321289
H	10.054630	-2.011426	-1.213466
H	8.990895	-0.798972	-0.463864
H	-8.966585	-0.866654	0.480893
H	-10.027701	-2.104304	1.191719
H	-10.010831	-2.220840	-1.346776
H	-9.361030	-3.703075	-0.611344
C	2.488005	-0.625751	-0.029121
H	2.544432	0.013021	-0.908014
H	2.558808	0.041352	0.827436
H	3.335389	-1.299949	-0.024244
H	-0.380827	-4.362022	0.014823
